# Programmable Pore Environments in Multivariate ZIF Membranes for Ultra-Selective Helium Recovery from Natural Gas

**DOI:** 10.1021/jacs.5c22294

**Published:** 2026-02-26

**Authors:** Yang Liu, Teng Li, Ziwen Fan, Wenjing Lv, Yining Liao, Zhenggong Wang, Michael D. Guiver, Daniel Maspoch, Jian Jin

**Affiliations:** † State Key Laboratory of Bioinspired Interfacial Materials Science & College of Chemistry, Chemical Engineering and Materials Science & Jiangsu Key Laboratory of Advanced Functional Polymer Materials, 12582 Soochow University, Suzhou 215123, P. R. China; ‡ State Key Laboratory of Engines, School of Mechanical Engineering, 12605 Tianjin University, Tianjin 300072, China; ⊥ Catalan Institute of Nanoscience and Nanotechnology (ICN2), CSIC, and The Barcelona Institute of Science and Technology, Bellaterra 08193, Spain; § Departament de Química, Universitat Autònoma de Barcelona (UAB), Cerdanyola del Vallè, Bellaterra 08193, Spain; ∥ ICREA, Pg. Lluís Companys 23, Barcelona 08010, Spain

## Abstract

Helium is an essential yet finite resource with critical applications in medical imaging and semiconductor manufacturing, whose production currently relies almost exclusively on energy-intensive cryogenic separation of trace helium from natural gas. Membrane-based separations offer an attractive alternative, but existing materials lack the selectivity required for industrial deployment. Here, we introduce a strategy for pore microenvironment programming in multivariate zeolitic imidazolate framework (MTV-ZIF) membranes, enabling ultraselective helium recovery under realistic feed gas conditions. By precisely combining Zn^2+^, 2-methylimidazole, and halogen-substituted benzimidazole linkers, we create synergistic combinations of steric constraints and enhanced CH_4_–framework interactions, which collectively suppress CH_4_ transport while preserving rapid He permeation. Under simulated industrial feed conditions (0.6% He/99.4% CH_4_ by volume), the best-performing membrane delivered a record He/CH_4_ selectivity of 3174, with stable operation over 960 h. Process simulations further show that a two-stage membrane cascade can deliver >99.95% He purity with an 83% reduction in energy demand compared to cryogenic distillation. These results highlight multivariate pore programming in MOFs as a powerful platform for efficient, low-energy He recovery from natural gas.

## Graphical abstract

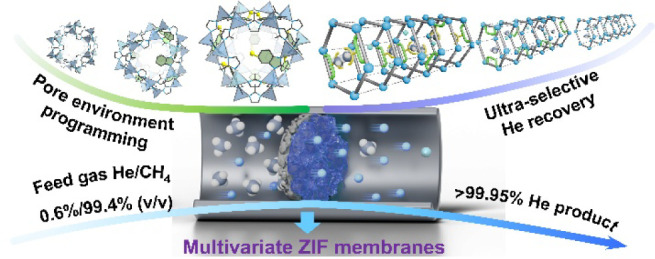

## Introduction

Helium (He) is a strategically important element with critical roles in medical imaging,^1^ semiconductor manufacturing, and defense.^2,3^ Its unique properties, including chemical inertness and an exceptionally low boiling point, underpin technologies ranging from MRI scanners to superconducting magnets and advanced chip fabrication. However, He is a finite resource: it is generated geologically over millions of years and occurs only as trace impurities in natural gas.^4–7^ Today, He is recovered almost exclusively through energy-intensive, capital-heavy cryogenic distillation, typically followed by pressure swing adsorption for final purification.^8–10^ This reliance on centralized infrastructure has led to recurrent supply shortages and price volatility, highlighting the need for more sustainable helium recovery methods.^11^

Membrane-based gas separations have emerged as an attractive alternative to cryogenic processing, offering a potentially lower energy demand, reduced carbon footprint, and operational simplicity.^12,13^ However, achieving high selectivity for He over CH_4_—the dominant component of natural gas—remains a challenge. The small kinetic diameter of He (2.6 Å) demands materials with subangstrom pore size precision, while its weak polarizability leads to minimal framework interactions. As a result, He/CH_4_ separation relies primarily on molecular sieving; however, nonrigid or defect-mediated transport pathways can permit CH_4_ leakage, leading to diminished selectivity. Conventional polymeric membranes exhibit insufficient selectivity and susceptibility to plasticization and swelling,^14–16^ and even advanced porous materials have struggled to deliver molecular sieving performance needed under realistic mixed-gas conditions.^17–20^

Metal–organic frameworks (MOFs) offer an unprecedented level of structural tunability^21–24^ and have widely explored in membrane technologies for diverse gas separations,^25–27^ including natural-gas upgrading,^28^ CO_2_ capture,^29^ H_2_ purification,^30,31^ and olefin/paraffin separation.^32,33^ However, their application to He recovery has remained limited by the lack of materials that simultaneously combine precise pore aperture control with tunable host–guest interactions (Figure 1a).^34–37^

**Figure 1:**
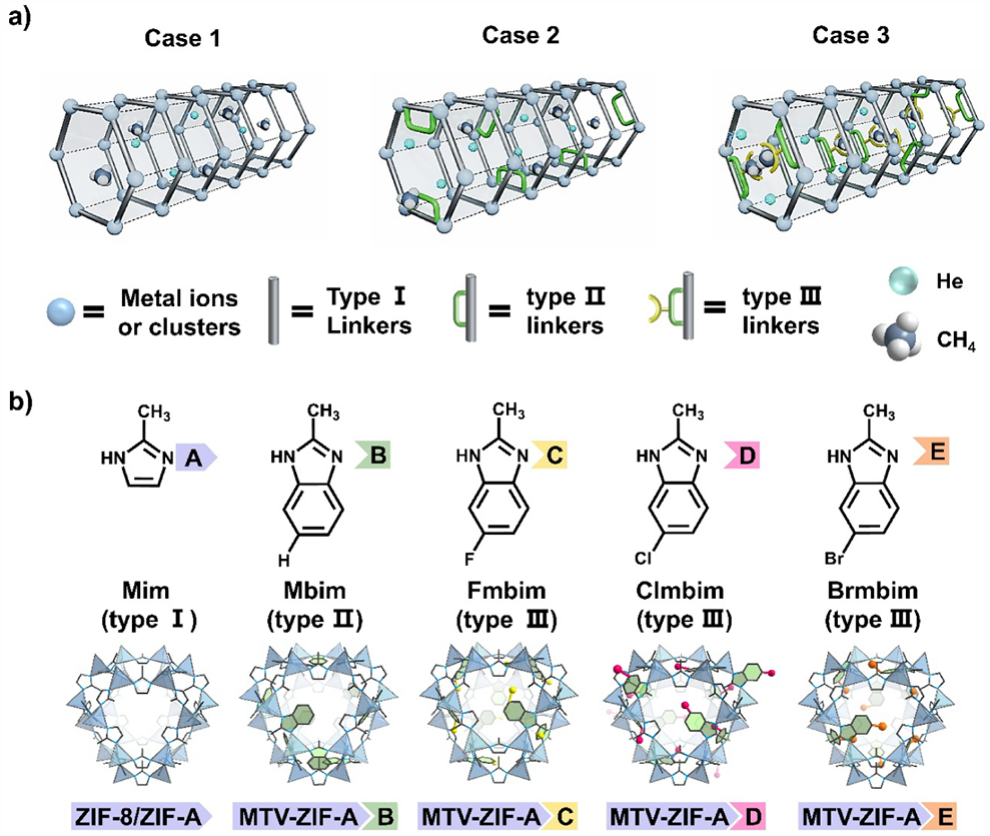
Schematic illustrations of programmable pore environment in MTV-ZIF membrane. a) Schematic illustration of pore-environment engineering for He separation. Case 1: a mismatched pore environment constructed by pristine type I linkers; Case 2: a narrowed pore size environment generated by sterically hindered type II linkers; Case 3: a finely tuned pore environment derived from type III linkers, combining pore-aperture contraction with host–guest interactions. b) Schematic illustration of the MTV-ZIFs constructed by mixing linker pairs: linker A: 2-methylimidazole (Mim); linker B: 2-methylbenzimidazole (Mbim); linker C: 5-fluoro-2-methyl-1*H*-benzoimidazole (Fmbim); linker D: 5-chloro-2-methyl-1*H*-benzimidazole (Clmbim); and linker E: 5-bromo-2-methyl-1*H*-benzimidazole (Brmbim).

In particular, multivariate MOFs (MTV-MOFs), in which different linkers are incorporated within the same framework, provide a versatile platform for tailoring pore environments at the molecular level.^38–40^ We hypothesized that strategic combinations of sterically bulky and chemically interactive linkers could create pore landscapes that simultaneously restrict CH_4_ diffusion and facilitate He permeation, together boosting selectivity.

Here, we report the design of multivariate ZIF membranes with programmable pore microenvironments, which deliver ultraselective He/CH_4_ separation under simulated natural gas conditions. By mixing Zn^2+^, 2-methylimidazole, and halogen-substituted benzimidazoles (Figure 1b), we create synergistic steric and interaction effects that elevate CH_4_ diffusion barriers while maintaining rapid He transport. These MTV-ZIF membranes achieve record He/CH_4_ selectivities and stable long-term operation, and process simulations demonstrate their potential to outperform cryogenic distillation in both energy efficiency and product purity. This work establishes pore programming in multivariate MOFs as a powerful strategy for designing next-generation molecular sieving membranes for critical gas separations including industrial helium recovery.

## Results and Discussion

### Programming Pore Environments in Multivariate ZIF Membranes: Design and Characterization

To precisely program the pore environment, we selected five imidazolate linkers, which are 2-methylimidazole (Mim, A), 2-methylbenzimidazole (Mbim, B), 5-fluoro-2-methylbenzimidazole (Fmbim, C), 5-chloro-2-methylbenzimidazole (Clmbim, D), and 5-bromo-2-methylbenzimidazole (Brmbim, E), as building blocks for MTV-ZIF membranes (Figure 1b). The halogen substituents introduce both steric hindrance and tunable host–guest,^41–43^ providing a versatile handle to tailor the pore environment. Four MTV-ZIF series were prepared by pairing linker A (Mim) with each functionalized benzimidazole at different molar ratios (*x* = 10–50% of total imidazolate content), denoted MTV-ZIF-A_(100–*x*)_B_*x*_, MTV-ZIF-A_(100–*x*)_C_*x*_, MTV-ZIF-A_(100–*x*)_D_*x*_ and MTV-ZIF-A_(100–*x*)_E_*x*_.

All membranes were grown on polyaniline-modified poly­(ether sulfone) flexible substrates using a low-temperature convection-diffusion method (Figure 2a and Figure S1). Lowering the synthesis temperature moderated metal-linker mobility and balanced the nucleation and growth of MTV-ZIFs incorporating the more strongly coordinating benzimidazole linkers, yielding highly ordered intergrown microstructures.^44,45^ Scanning electron microscopy (SEM) images revealed continuous, defect-free selective layers across all compositions (Figure 2b and Figures S2, S4, S6 and S8), and energy-dispersive X-ray (EDX) spectroscopy mapping confirmed uniform linker distributions within the membrane plane (Figures S3, S5, S7 and S9). Cross-sectional SEM images showed a systematic reduction in thickness with increasing benzimidazole-linker content; for example, raising the Fmbim fraction from 10% to 45% reduced the selective-layer thickness from ∼435 nm to ∼210 nm (Figure 2c and Figures S2–S8). This trend is attributed to low-temperature crystallization, which balances nucleation and growth between adjacent crystals, leading to thinner and more compact intergrown layers. Optical images displayed thin-film interference for most membranes, consistent with smooth, uniform layers (Figure 2a and Figures S10-S13). At higher benzimidazole-linker contents, however, thin-film interference disappeared, and SEM images revealed loosely deposited particles, indicating a failure to form continuous layers (Figures S2f, S4g, S6f, and S8e).

**Figure 2:**
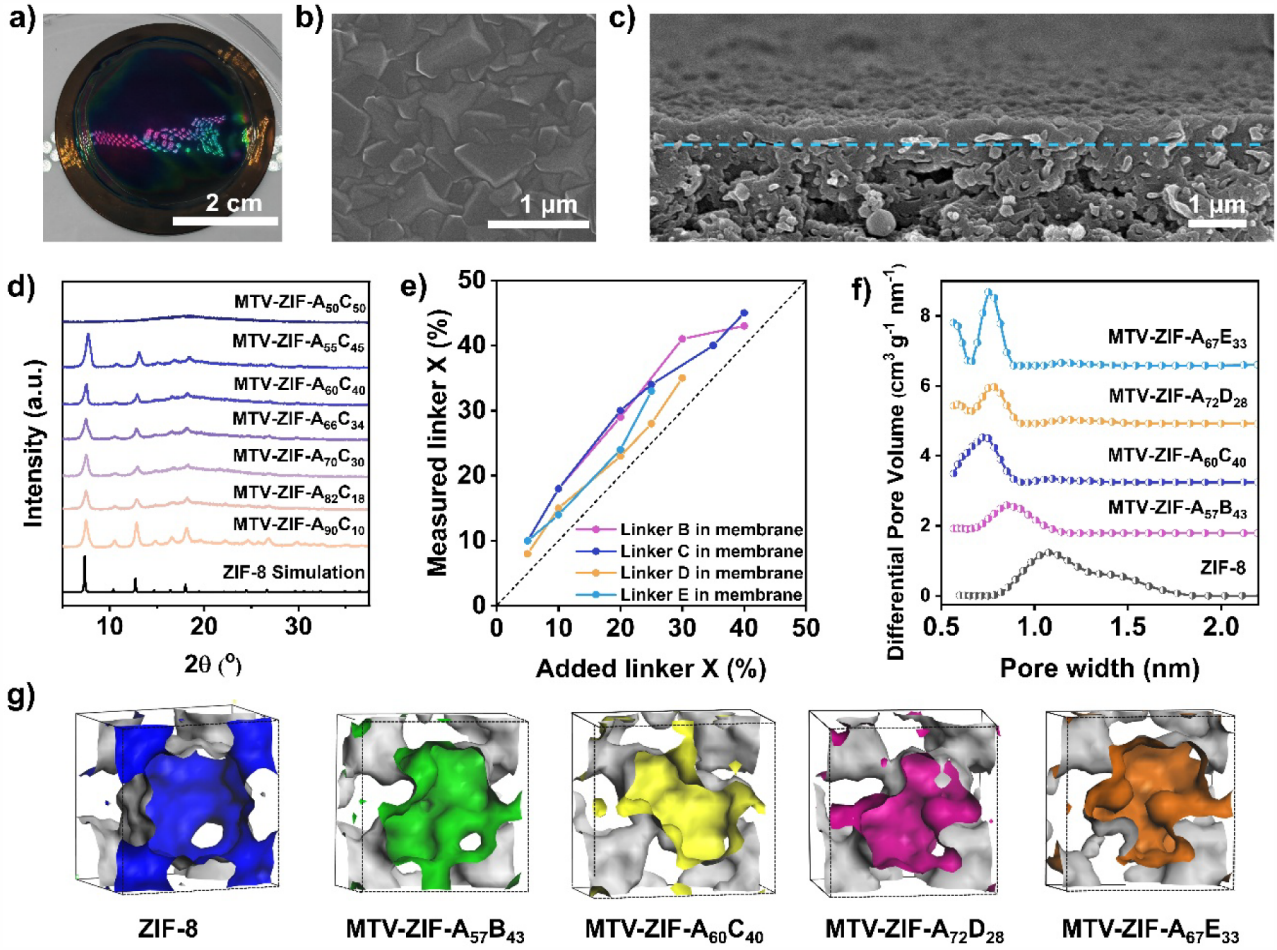
Structure characterization of MTV-ZIF membranes. a) Optical photo of MTV-ZIF-A_60_C_40_ membrane. b) Surface SEM image of MTV-ZIF-A_60_C_40_ membrane. c) Cross-sectional SEM images of MTV-ZIF-A_60_C_40_ membrane. d) PXRD patterns of MTV-ZIF-A_(100–*x*)_C*_x_* membranes. e) Comparison of added and real percentages of linkers in MTV-ZIF membranes. f) Pore size distributions for ZIF-8, MTV-ZIF-A_57_B_43_, MTV-ZIF-A_60_C_40_, MTV-ZIF-A_72_D_28_, and MTV-ZIF-A_67_E_33_. g) Simulated gas transport channels within the frameworks.

Structural analyses confirmed that all MTV-ZIF membranes retain the ZIF-8 topology across a broad range of linker ratios. Powder X-ray diffraction (PXRD) patterns matched those of the parent ZIF-8 phase (Figure 2d and Figures S14–S16). Even at high benzimidazole loadings, such as MTV-ZIF-A_57_B_43_, MTV-ZIF-A_60_C_40_, MTV-ZIF-A_72_D_28_, and MTV-ZIF-A_67_E_33_, membranes remained crystalline and randomly oriented, as evidenced by sharp, ordered diffraction rings in grazing-incidence wide-angle X-ray scattering (GIWAXS) patterns (Figures S17–S19). Beyond 50% Mbim or Fmbim, and 40% Clmbim or Brmbim, crystallinity declined sharply, and membrane formation failed, reflecting steric and coordination mismatches that disrupt framework nucleation and growth; consistent with SEM observations (Figures S2f, S4g, S6f and S8e).

Linker incorporation was quantified by ^1^H nuclear magnetic resonance (^1^H NMR) after acid digestion, showing proportional increases in aromatic proton signals with added benzimidazole content (Figures S20–S23 and Tables S1–S4). For example, increasing Fmbim in the precursor solution from 5% to 40% led to its fraction in the membrane increasing from 10% to 45% (Figure 2e), consistent with its strong coordination to Zn­(II) ions. Attenuated total reflectance Fourier transform infrared spectroscopy (ATR-FTIR) further revealed intensified aromatic asymmetric stretching (C=C, 1480 cm^–1^) and out-of-plane bending (C–H, 740–800 cm^–1^) bands with increasing benzimidazole-linker content (Figures S24–S27), while the Mim C–H stretching band (1300 cm^–1^) decreased.

To probe microporosity, MTV-ZIF nanoparticles were analyzed by N_2_ adsorption at 77 K. All samples displayed type I isotherms, with Brunauer–Emmett–Teller (BET) surface areas and pore volumes decreasing systematically with increasing benzimidazole fraction (Figure 2f and Figures S28–S31). For example, MTV-ZIF-A_57_B_43_, -A_55_C_45_, -A_65_D_35_, and -A_67_E_33_ showed BET surface areas of 740, 829, 874, and 778 m^2^ g^–1^, respectively, versus 1350 m^2^ g^–1^ for ZIF-8 (Figures S28b-S31b). The corresponding cumulative pore volumes dropped to 0.64, 0.82, 0.72, and 0.78 cm^3^ g^–1^, compared with 1.45 cm^3^ g^–1^ for ZIF-8 (Figures S28c–S31c). Pore size distributions (PSD) revealed narrower micropores (0.5–1.0 nm) for all MTV-ZIFs compared to the broader 0.8–1.8 nm distribution of ZIF-8 (Figure 2f and Figures S28d, S29d, S30d, and S31d). Interestingly, at higher benzimidazole contents, bimodal distributions emerged in MTV-ZIF-AD and -AE, likely reflecting steric effects of the bulkier substituents that create distinct local pore structures, whereas MTV-ZIF-AB and -AC remained narrowly distributed.

Finally, grand canonical Monte Carlo (GCMC) simulations corroborated the progressive reduction in pore size. For MTV-ZIF-AC, increasing the Fmbim fraction from 10% to 40% reduced the average pore size from 1.1 to 0.63 nm (Figure S32), in agreement with N_2_ physisorption. Similar trends were observed for MTV-ZIF-AB, -AD and -AE. To visualize transport pathways, structural models examined along (100) and (111) planes (Figure 2g and Figures S33–S38) showed regular, symmetric cavities in ZIF-8, but constricted, irregular and asymmetric pores in MTV-ZIFs, consistent with the designed pore programming strategy.

### Single-Gas Transport and Selectivity Tuning

Having established the structural tunability of the MTV-ZIF membranes, we next examined how pore-environment programming translates into gas transport behavior, using single-gas permeation measurements in a custom Wicke–Kallenbach setup (Figure S39). Binary He/CH_4_ separations were performed for the four MTV-ZIF-AB, -AC, -AD, and -AE membrane series to probe the impact of linker chemistry on molecular sieving.

In the MTV-ZIF-AC series, increasing the Fmbim content from 0 to 30 mol % led to a gradual decline in both He and CH_4_ permeances. At 30 mol % Fmbim, He permeance reached 983 GPU (GPU: gas permeation unit, 1 GPU = 3.35 × 10^–10^ mol m^–2^ s^–1^ Pa^–1^), while CH_4_ permeance decreased by nearly 90%, giving a sharp rise in He/CH_4_ selectivity from 13 to 103. Further raising Fmbim to 45 mol % caused only a modest drop in He permeance but further increased selectivity. The MTV-ZIF-A_60_C_40_ membrane (Mim:Fmbim = 3:2) exhibited the best performance, with a record He/CH_4_ selectivity of 418 and high He permeance of 1137 GPU (Figure 3a). At higher Fmbim loadings, excessive steric hindrance began to obstruct He transport pathways, leading to a decline in both permeance and selectivity. In contrast, the parent ZIF-8 membrane (MTV-ZIF-A_100_C_0_) exhibited poor separation performance, permeating both gases freely due to its larger pore size.

**Figure 3:**
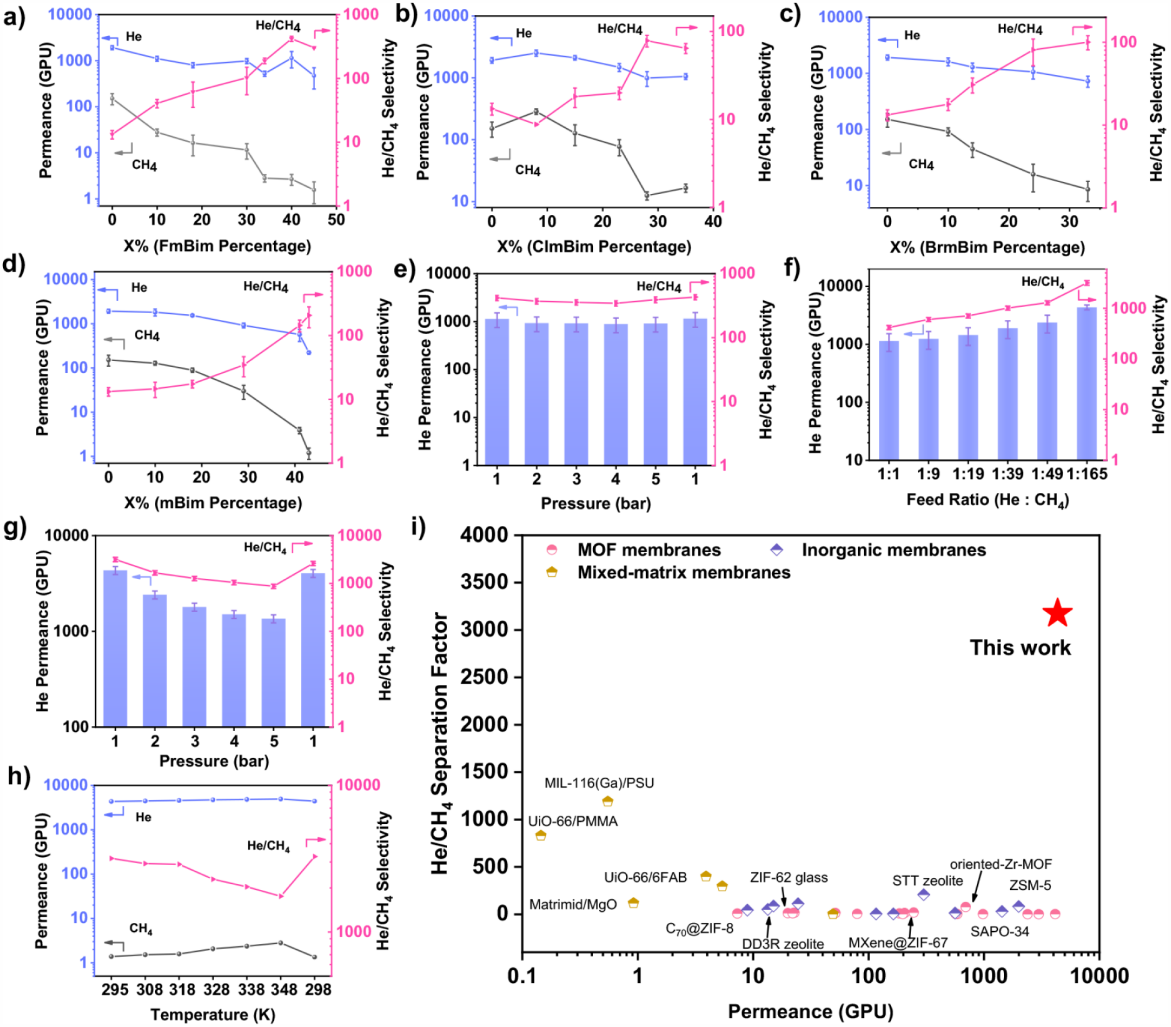
He/CH_4_ mixed-gas separation performances of MTV-ZIF membranes. He/CH_4_ mixed-gas separation performances of MTV-ZIF-A_(100–*x*)_C*_x_* (a), MTV-ZIF-A*_(_*_100–*x*)_D*_x_* (b), MTV-ZIF-A*_(100‑x)_* E*_x_* (c), and MTV-ZIF-A*_(100‑x)_* B*_x_* (d) membranes. e) Pressure dependence of the He/CH_4_ separation performance for the MTV-ZIF-A_60_C_40_ membrane. f) Feed composition dependence of the He/CH_4_ separation performance for the MTV-ZIF-A_60_C_40_ membrane. g) Pressure dependence of the He/CH_4_ separation performance for the MTV-ZIF-A_60_C_40_ membrane under 0.6% He/99.4% CH_4_ feed. h) Temperature dependence of the He/CH_4_ separation performance for the MTV-ZIF-A_60_C_40_ membrane under 0.6% He/99.4% CH_4_ feed. Each value represents the average of three different membranes; error bars denote standard deviations (Tables S5–S8). (i) Comparison of the He/CH_4_ separation performance for the MTV-ZIF-A_60_C_40_ membranes with other reported membranes. Note that our data was collected at 0.6% He/99.4% CH_4_ mixed feed gas under process-mimicking conditions, whereas most reported values correspond to feed compositions or single-gas tests. Comparative data are summarized in Table S9.

Similar behavior was observed for MTV-ZIF-AD and -AE membranes. MTV-ZIF-A_72_D_28_ and MTV-ZIF-A_67_E_33_ membranes achieved He/CH_4_ selectivities of 78 and 100, with He permeances of 991 and 727 GPU, respectively (Figure 3b and c). In the MTV-ZIF-AB series, raising Mbim content to 29% increased selectivity from 13 to 34 but reduced He permeance to 917 GPU. Above 30% Mbim, CH_4_ permeance decreased sharply, likely due to size exclusion. At 43% Mbim, He permeance fell by 61% (to 222 GPU), indicating that aperture contraction begins to hinder He diffusion (Figure 3d). Importantly, MTV-ZIF-AC, -AD, and -AE membranes sustained higher permeance and selectivity at elevated benzimidazole loadings compared to MTV-ZIF-AB membranes, demonstrating that performance gains arise not only from pore contraction but also from increased polarity and irregular pore environments introduced by halogenated linkers. This is consistent with pore size analyses (Figure 2f), where MTV-ZIF-A_60_C_40_, -A_72_D_28_, and -A_67_E_33_ exhibited smaller pores than MTV-ZIF-A_57_B_43_ yet sustained high He permeance; indicating a synergistic effect of polarity and pore irregularity in enhancing molecular sieving. Overall, MTV-ZIF-A_60_C_40_ represents the optimal balance, coupling high selectivity with high He permeance.

To assess the general molecular sieving behavior, we further measured single-gas permeation of H_2_, CO_2_, N_2_, CH_4_, C_2_H_4_, C_2_H_6_, C_3_H_6_, and C_3_H_8_ on the MTV-ZIF-A_60_C_40_ membrane. Permeance decreased systematically with kinetic diameter (Figure S40a), confirming size-sieving control. The ideal selectivities for CO_2_/CH_4_, H_2_/CH_4_, He/N_2_, He/CH_4_, He/C_3_H_6_ and He/C_2_H_4_ were 43.3, 158.7, 246.0, 434.0, 3246.3, and 3641.3, respectively (Figure S40b), all far exceeding the corresponding Knudsen selectivities and confirming a defect-free selective layer. Notably, the ideal selectivity for He/N_2_ and He/CH_4_ closely matched binary gas separation results, underscoring the effectiveness of pore-environment engineering in controlling molecular transport (Figure S41).

### Mixed-Gas Separation under Various Feed Conditions

To evaluate the operational suitability of MTV-ZIF membranes, we examined their performance under various feed gas pressure, operating temperature, and feed gas composition. Increasing the feed pressure from 1 to 5 bar led to a moderate decline in He permeance from 1137 to 916 GPU, while He/CH_4_ selectivity dropped only 17% (from 418 to 344), maintaining strong separation performance. At 5 bar, selectivity rebounded to levels similar to those at 1 bar, fully recovering those levels once pressure was reduced back to 1 bar (Figure 3e). Consistent behavior was observed during pressure-cycling experiments, confirming the reversibility of the pressure response and the membrane’s resistance to irreversible compaction (Figure S42). This reversibility highlights the membrane’s pressure resilience, likely due to framework rigidification induced by mixed linkers and enhanced adsorption in tailored pores.

Temperature-dependent permeation using an equimolar He/CH_4_ mixture showed thermally activated diffusion for both gases (Figure S43a). However, CH_4_ permeance increased more steeply than He due to its higher apparent activation energy (17.32 kJ mol^–1^ vs 8.25 kJ mol^–1^), reflecting its larger kinetic diameter and stronger framework interactions (Figure S43b). As a result, He/CH_4_ selectivity gradually decreased with temperature but remained above 200 even at 348 K. Importantly, performance fully recovered on cooling back to 303 K, confirming excellent thermal stability.

Since industrial natural gas typically contains less than 4% He, we evaluated the MTV-ZIF-A_60_C_40_ membrane under progressively dilute He feeds. As shown in Figure 3f, the membrane maintained excellent separation performance across the full range. As the He concentration decreased from 50% to 2%, the He/CH_4_ selectivity nearly doubled and He permeance rose to 2368 GPU, about twice the initial value. Under a realistic 0.6% He content, the membrane achieved a remarkable He permeance of 4341 GPU and a He/CH_4_ selectivity of 3174, confirming outstanding performance at trace concentrations (Figure 3f).

Further pressure tests under low He feeds (0.6%) showed that increasing pressure from 1 to 5 bar reduced He permeance from ∼4341 to ∼1355 GPU and He/CH_4_ selectivity from ∼3174 to ∼870 (Figure 3g), likely due to enhanced competitive CH_4_ adsorption at high pressure. Nonetheless, performance remained far superior to previously reported MOF membranes under comparable conditions (Table S9).

Upon returning to 1 bar, both permeance and selectivity fully recovered, indicating reversibility and robust pressure tolerance (Figure S44). Temperature variations showed consistent higher permeance for both gases upon heating. However, due to the higher apparent activation energy of CH_4_, the He/CH_4_ selectivity declined. At 348 K, He permeance reached 4926 GPU, with selectivity maintained around 1753. On cooling back to room temperature, the separation performance was fully restored (Figure 3h), confirming reversible, stable behavior under operational fluctuations.

Figure 3i benchmarks the MTV-ZIF-A_60_C_40_ membrane against state-of-the-art He-separation membranes (Figure S45 and Table S9). At 1 bar and equimolar feeds, its He/CH_4_ selectivity exceeds that of other MOF-based membranes by an order of magnitude. Under simulated low-He concentrations (0.6%), it outperforms all reported inorganic and MOF membranes by 2 orders of magnitude and even surpasses most polymer membranes. Notably, while most literature values are measured under equimolar feed conditions, our results at highly dilute He compositions underscore the practical industrial relevance of the MTV-ZIF-A_60_C_40_ membrane for helium recovery.

### Mechanistic Insights into Pore-Environment-Driven Sieving

To rationalize the high He/CH_4_ separation performance, we investigated how pore environment manipulation influences framework rigidity, adsorption behavior, and host–guest interactions. Rietveld refinements revealed that nearly 74% of MTV-ZIF-A_60_C_40_ adopts the rigid *I*4̅3*m* phase of ZIF-8, compared to only 24% in ZIF-8 (Figure 4a and Figure S46). This increased fraction of the rigid phase reflects steric restriction by mixed linkers, which stabilizes the framework and narrows apertures (∼3.4 Å).^46^ Such rigidification suppresses gate-opening flexibility and enhances molecular sieving precisions

**Figure 4:**
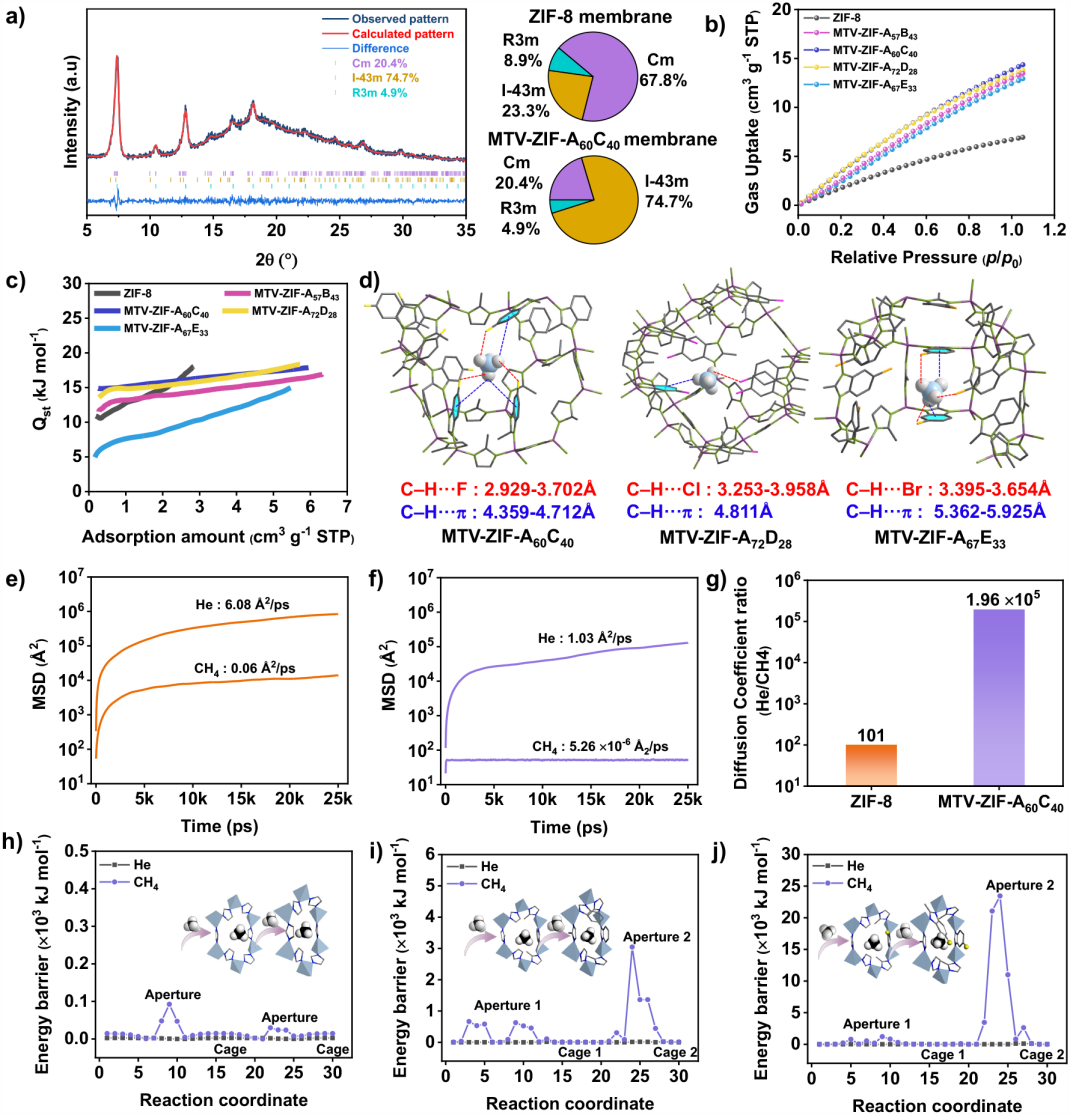
Investigation of He/CH_4_ separation mechanism in MTV-ZIF membranes. a) Rietveld refinement of PXRD results for the MTV-ZIF-A_60_C_40_ membrane (left). Pie chart of the content profile of different polymorphs in the ZIF-8 membrane and the MTV-ZIF-A_60_C_40_ membrane (right). b) CH_4_ adsorption isotherms of MTV-ZIFs at 273 K. c) Isosteric heats of CH_4_ adsorption in MTV-ZIFs. d) CH_4_ adsorption sites in MTV-ZIF-A_60_C_40_, MTV-ZIF-A_72_C_28_ and MTV-ZIF-A_67_C_33_ identified by GCMC simulation. The closest contacts between the framework atoms and the respective gas molecules are characterized by the distances, measured in Å. Mean square displacement (MSD) of He and CH_4_ diffusing through ZIF-8 (e) and MTV-ZIF-A_60_C_40_ (f) along with time. g) Ratios of He to CH_4_ diffusion coefficients in ZIF-8 and MTV-ZIF-A_60_C_40_. h–j) Energy profile for He and CH_4_ transport through ZIF-8 (h), MTV-ZIF-A_57_B_43_ (i) and MTV-ZIF-A_60_C_40_ (j). Two distinct six-membered pore-window configurations are possible in MTV-ZIF-A_57_B_43_ and MTV-ZIF-A_60_C_40_: (i) one Mim linker replaced by a benzimidazole linker (Aperture 1), and (ii) two Mim linkers replaced by benzimidazole linkers (Aperture 2).

As expected for an inert gas, He adsorption at 273 K was negligible for all samples (Figure S48). In contrast, CH_4_ adsorption isotherms (273 and 298 K) showed significantly higher uptakes in MTV-ZIF-A_60_C_40_ (14.4 and 10.2 cm^3^ g^–1^ at 273 and 298 K) than in ZIF-8 (6.9 and 6.6 cm^3^ g^–1^; Figure 4b and Figure S47). Other MTV-ZIFs (-A_57_B_43_, -A_72_D_28_, and -A_67_E_33_) also exhibited increased CH_4_ uptakes relative to ZIF-8, indicating that linker-induced pore environments strengthen CH_4_ adsorption while slowing its diffusion.^47^ The CH_4_ isosteric heat of adsorption (*Q*_st_) determined by Clausius–Clapeyron analysis of adsorption isotherms at 273, 298, and 313 K revealed a zero-coverage *Q*_st_ of 14.9 kJ mol^–1^ for MTV-ZIF-A_60_C_40_ (Figure 4c). This value exceeds those of other samples, reflecting stronger host–guest interactions,^48^ and is consistent with GCMC-calculated adsorption energies (Figure S49).

Structural optimization with CH_4_ revealed that halogen-substituted benzimidazole linkers generate multiple C–H···halogen and C–H···π interactions within the frameworks.^49^ MTV-ZIF-A_60_C_40_ exhibits the strongest synergistic effect, with simultaneous C–H···F (2.929–3.702 Å) and C–H···π (4.359–4.712 Å) interactions, surpassing those in the other MTV-ZIFs (Figure 4d and Figure S50). Electrostatic potential (ESP) maps further highlight that fluorine atoms in Fmbim introduce highly negative regions that preferentially interact with the positive ESP of CH_4_ (Figure S51). Together, these results confirm that synergistic steric and electronic effects introduced by mixed linkers is responsible for the exceptional He/CH_4_ separation performance observed in MTV-ZIF-A_60_C_40_.

Molecular dynamics (MD) simulations provided further insight into how pore-environment programming modulates molecular transport. In ZIF-8, both He and CH_4_ diffuse freely, whereas in MTV-ZIF-A_60_C_40_, motion is markedly slower, especially for CH_4_ (Figure 4e and f). The calculated He/CH_4_ self-diffusion coefficient ratio increased dramatically from 100 in ZIF-8 to 1.96 × 10^5^ in MTV-ZIF-A_60_C_40_, evidencing a strong suppression of CH_4_ mobility that goes beyond simple size sieving (Figure 4g). Energy profiles corroborated these trends. In pristine ZIF-8, both molecules cross pore windows with low energy barriers and diffuse rapidly inside the large cage (Figure 4h and Figure S52). In contrast, MTV-ZIF-A_60_C_40_ exhibits substantially increased CH_4_ diffusion energy barriers at the two different pore windows (1.2 × 10^3^ and 24 × 10^3^ kJ mol^–1^), whereas the He diffusion energy barrier remained low (Figure 4j). This disparity explains the pronounced reduction in CH_4_ permeance with only a modest change in He permeance, thereby enhancing He/CH_4_ selectivity. For comparison, MTV-ZIF-A_57_B_43_ (with similar mixed-linker content but lacking halogen functionality) exhibits a much lower CH_4_ diffusion energy barriers (0.6 × 10^3^ and 3 × 10^3^ kJ mol^–1^) (Figure 4i). This indicates that the high CH_4_ energy barrier in MTV-ZIF-A_60_C_40_ arises not only from window contraction by bulky linkers but also from specific CH_4_–Fmbim interactions that further hinder CH_4_ transport.

### Process-Level Performance and Industrial Prospects

Extended-term stability of the MTV-ZIF-A_60_C_40_ membrane under practical conditions was validated by a 960-h continuous separation test (Figure 5a). Performance remained stable at 1 bar for 480 h, with no observable degradation. Following the introduction of ethane (a typical natural-gas impurity), the selectivity showed a slight, transient decrease but quickly recovered to baseline and remained stable for 230 h. At 5 bar over 240 h, permeance and selectivity declined moderately yet maintained strong separation, demonstrating tolerance to sustained high-pressure operation. Once the pressure was switched back to 1 bar, He/CH_4_ separation performance fully recovered, confirming excellent pressure resilience and long-term stability. Mechanical bending tests further confirmed robustness: after 10 bending cycles at 333 m^–1^ curvature, no cracks, detachment, or fragmentation were observed, and separation performance remained unchanged (Figure S53). Furthermore, although the membrane elongation decreased, the membrane stress and Young’s modulus were significantly increased compared to the bare support due to the rigidity of the ZIF layer (Figure S54).

**Figure 5:**
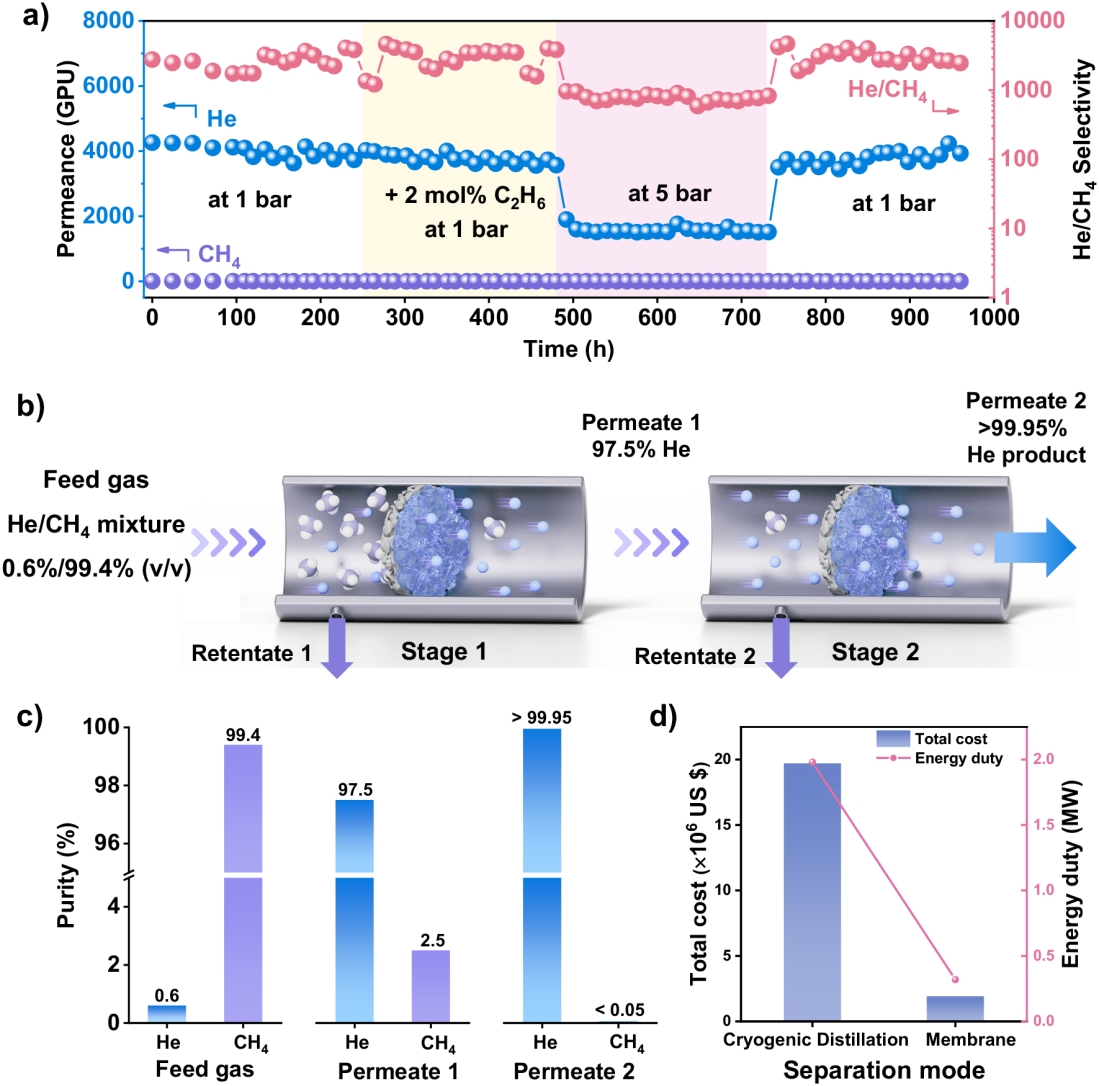
Separation stability of MTV-ZIF-A_60_C_40_ membranes and He extraction performance evaluations in a two-stage cascade membrane system. a) Extended-term stability test of the MTV-ZIF-A_60_C_40_ membranes under the mimicking real-world conditions (measured at 1 or 5 bar and 298 K, feed compositions: 0.6 He: 99.4 CH_4_ with or without 2 mol % C_2_H_6_). b) Schematic of the two-stage cascade membrane process for He enrichment from low-He natural gas. c) Simulated feed gas composition and corresponding product purities at each stage of the two-stage cascade membrane separation process. d) Energy consumption and total cost of membrane separation versus cryogenic distillation at 0.6% He/99.4% CH_4_ feed.

To assess the industrial potential of MTV-ZIF membranes, we modeled a two-stage membrane cascade for He recovery from low-He natural gas feeds (0.6% He/99.4% CH_4_; Figure 5b). In the first stage, the MTV-ZIF-A_60_C_40_ membrane enriched the He content of the permeate to 97.5%. A second cascade stage further reduced CH_4_ below GC detection limits, yielding He purity >99.95%, and a He recovery rate of 80.0%, fully meeting industrial-grade specifications (Figure 5c and Figure S55). Additionally, the CH_4_-rich retentate from each stage can be recyclable, improving process efficiency and resource utilization.

To evaluate the potential energy and cost savings of our two-stage membrane cascade separation process for He extraction from natural gas, process simulations were performed using Aspen Plus (Figures S56 and S57).^50–53^ For comparison, a conventional cryogenic distillation process was modeled using the same feed composition and a helium purity target of >99.95%. The conventional cryogenic process requires deep refrigeration, resulting in a total energy load of 1.98 MW and an estimated annualized cost (capital and utilities) of US$19 million for processing 100,000 m^3^ of gas (Figure 5d). By contrast, the two-stage cascade membrane process achieved an 83% reduction in energy consumption, requiring only 0.32 MW, and lowered total annual costs to approximately US$1.91 million; around one-tenth of the cryogenic benchmark. For low concentration He feeds, this membrane-based process can replace the cryogenic column, while the high He/CH_4_ selectivity enables direct recycling of purified CH_4_ retentate, eliminating column-related energy use altogether.

## Conclusion

In summary, we have developed a multivariate ZIF platform that enables precise programming of pore microenvironments through the incorporation of tunable fractions of halogenated linkers. The resulting MTV-ZIF membranes offer angstrom-level control over pore apertures and framework–guest interactions, enabling ultraselective He/CH_4_ separation. A combination of experimental and computational studies reveals that their exceptional performances stems from the synergistic interplay of molecular sieving and host–guest interactions: in fluorinated MTV-ZIFs, aperture contraction together with strengthened CH_4_-framework interactions raises CH_4_ diffusion barriers while preserving rapid He transport, yielding both high He/CH_4_ selectivity and high He permeance. Under process-relevant conditions with trace He feeds, these membranes allow continuous enrichment of He with concomitant excellent selectivity and stable permeance. A two-stage cascade produced high-purity He with remarkably low energy consumption and cost compared to cryogenic distillation. This work not only demonstrates the potential of MTV-ZIF membranes for energy-efficient He recovery from natural gas, but also establishes a versatile linker-engineering strategy for designing MOF membranes with programmable pore environments applicable to various molecular separations.

## Supplementary Material



## Notes

Detailed experimental section and characterization, including PXRD diffractograms, FTIR, NMR, N_2_ and CH_4_ adsorption, and gas separation tests (PDF)
