# Supplementary material for: Programmable Pore Environments in Multivariate ZIF Membranes for Ultra-Selective Helium Recovery from Natural Gas
Source: J Am Chem Soc. 2026 Feb 26;148(9):9910–9. doi: 10.1021/jacs.5c22294 (PMC12983314; doi:10.1021/jacs.5c22294)
Supplement: Supplementary file 1 [file ja5c22294_si_001.pdf]

Supporting Information

Programmable Pore Environments in Multivariate ZIF Membranes for Ultra-Selective Helium Recovery from Natural Gas

Yang Liu,[†] Teng Li,[†] Ziwen Fan,[†] Wenjing Lv,[†] Yining Liao,[†] Zhenggong Wang,[*,†] Michael D. Guiver,[‡] Daniel Maspoch,[*,§,⊥,#] Jian Jin[*,†]

[†]State Key Laboratory of Bioinspired Interfacial Materials Science & College of Chemistry, Chemical Engineering and Materials Science & Jiangsu Key Laboratory of Advanced Functional Polymer Materials, Soochow University, Suzhou, 215123, P. R. China.

[‡]State Key Laboratory of Engines, School of Mechanical Engineering, Tianjin University, Tianjin, 300072, China.

[§]Catalan Institute of Nanoscience and Nanotechnology (ICN2), CSIC, and The Barcelona Institute of Science and Technology, Bellaterra 08193, Spain.

[⊥]Departament de Química, Universitat Autònoma de Barcelona (UAB), Cerdanyola del Vallès, Bellaterra 08193, Spain.

[#]ICREA, Pg. Lluís Companys 23, Barcelona 08010, Spain.

To whom correspondence may be addressed. E-mail: zgwang2017@suda.edu.cn; daniel.maspoch@icn2.cat; jjin@suda.edu.cn

Table of Contents

Section 1. General methods and materials S3

Section 2. Supporting figures S10

Section 3. Supporting tables.. S67

Section 4. References S81

1. General methods and materials

Materials

Zinc nitrate hexahydrate (98.0%) was acquired from Sigma Aldrich. 2-Methylimidazole (Mim, 99%) and 2-methylbenzimidazole (Mbim, 98%) were obtained from TCI Chemical. 5-fluoro-2-methylbenzoimidazole (FmBim, 97%), 5-chloro-2-methylbenzoimidazole (ClmBim, ≥98%), 5-bromo-2-methylbenzoimidazole (BrmBim, 97%) were purchased from Bidepharm. Aniline (99.5%), anhydrous methanol (99.5%), and ammonium hydroxide (28-30% aqueous solution) were acquired from Sinopharm. Perchloric acid (70.0-72.0%) was acquired from Aladdin. Dimethyl sulfoxide-d_6 (DMSO-d_6, 99.9%) and sulfuric acid-d_2 (D_2SO_4-d_2, 99.5%) were purchased from Macklin. Polyethersulfone (PES) membranes (diameter of 47 mm, pore size of 0.1 μm, M_w=147,700 g mol^{-1}, M_n=67,500 g mol^{-1}) were obtained from Haining Yibo Filter Equipment Factory of Zhejiang Province, China. All chemical reagents and solvents were used as received without further purification.

Characterization

Powder X-ray diffraction (PXRD) patterns of the samples were detected under ambient conditions using a Shimadzu XRD-6100 instrument fitted with Cu Kα radiation of wavelength (λ) 1.54 Å. ^{1}H nuclear magnetic resonance (^{1}H NMR) spectra were recorded on a Bruker 400 MHz spectrometer using sulfuric acid-d_2 solution and dimethyl sulfoxide-d_6 as solvent. Field-emission scanning electron microscopy (SEM) images were collected on a scanning electron microscope (Hitachi SU8230). ATR-FTIR spectra of the samples were recorded with a Nicolet 6700 spectrometer. Grazing

incidence wide-angle X-ray scattering (GIWAXS) measurements were conducted at a Xeuss 3.0 at Vacuum Interconnected Nanotech Workstation (Nano-X) from Suzhou Institute of Nano-Tech and Nano-Bionics, Chinese Academy of Sciences. The incident angle was set to 0.2 degree. Mechanical properties of polymer membranes were carried out by using an electronic Universal Testing Machine (Instron 3365). Membranes were cut into strips with an effective length of 15 mm and a width of 5 mm. These membranes were stretched at the rate of 3 mm/min. Tensile strength at break, Young's modulus, and elongation at break were recorded.

N_2 sorption isotherms were collected at 77 K (N_2) using an Autosorb iQ2 (Quantachrome). Temperature was controlled by using a liquid nitrogen bath. Prior to measurement, the samples were regenerated at 120 °C under vacuum (~1 × 10^{-5} bar) for ~12 h. The specific surface area was calculated from the Brunauer-Emmett-Teller (BET) adsorption method in the range of 0.001 - 1.000 p/p_0. Total pore volume (Vt) was calculated at p/p_0 = 0.979. Pore size distribution was evaluated using a nonlocal density functional theory (NLDFT) model by assuming a carbon-slit pore geometry provided by the software package version 5.21 of Quantachrome Instruments. CH_4 adsorption isotherms at 273 K, 298 K and 313 K were obtained using a Micromeritics ASAP 2460. The isosteric heats of adsorption for all the gases were calculated using the isotherms at 273 K, 298K and 313 K, following the Clausius-Clapeyron equation. It was done with the calculation program embedded in the software of Micromeritics ASAP 2460. High accuracy of the Q_{st} was found in all the calculations as evidenced by the linearity in the isosters.

Gas permeation measurement

The obtained membranes were sealed in a Wicke-Kallenbach cell to measure their separation performance (Figure S38).[1-2] To avoid direct contact of silicone O-ring with

the measured membrane and thus damage the membrane, two aluminum tapes with 10 mm diameter holes were used to sandwich the membrane and then high vacuum grease was used to seal the aluminum tapes and the membrane. Single gas permeance experiment was performed using the steady-state gas of He, H_2, CO_2, CH_4, C_2H_4, C_2H_6, C_3H_6 and C_3H_8 at 25 °C. The feed flow rate was constant at 30 mL min^{-1}, regulated by mass flow controllers. Argon with flow rate of 10 mL min^{-1} was used as a sweep gas to minimize the influence of back diffusion of the sweeping gas to the feed side and simultaneously bring permeate into a gas chromatography (GC, Agilent Technologies 7820A) for composition analysis. There was no pressure drop between the sides of the membrane in order to prevent any damage on the membrane.

$$Q_i = \frac{N_i}{\Delta p_i \times \mathrm{A}} \qquad (1)$$

where N_i (mol·s^{-1}) is the molar flow rate of component i, Δp_i (Pa) is the transmembrane pressure difference of component i, and A (m^2) is the effective membrane area for testing. The ideal selectivity, $S_\mathrm{i/j}$, is calculated from the relation between the permeance of component i and component j.

$$S_{i/j} = \frac{Q_i}{Q_j} \qquad (2)$$

For the mixed gas separation measurement, the prepared MOF membrane was fixed in a module sealed with O-rings. A 1:1 mixture of gas was applied to the feed side of the membrane, and the permeate gas was removed from the permeate side with the sweeping gas (Ar)[3]. The feed flow rate was kept constant with a total volumetric flow rate of 100 mL·min^{-1} (each gas, 50 mL·min^{-1}). Pressures at both the feed side and permeate side were maintained at 1 bar. A calibrated gas chromatograph was used to measure the concentration of each gas on the permeate side. The selectivity $\alpha_\mathrm{i/j}$, of the gas pairs is defined as the molar ratios of the components (i, j) in the permeate side,

divided by the quotient of the molar of the components (i, j) in the feed side:

$$\alpha_{i/j}=\frac{x_{i,perm}/x_{j,perm}}{x_{i,feed}/x_{i,feed}} \qquad (3)$$

Methods

Preparation of the PANI/PES composite substrate. The composite substrate was prepared following a reported procedure.[3] Briefly, aniline (0.930 g) and ammonium persulfate (0.685 g) were dissolved in 50 mL of 1.0 M perchloric acid and mixed thoroughly, then placed in an ice–water bath to initiate polymerization. A polyethersulfone (PES) membrane was immersed in the reaction solution and gently stirred for 12 h to allow in situ growth of polyaniline (PANI). The resulting PANI/PES substrate was rinsed sequentially with deionized water, 0.1 M ammonium hydroxide, and deionized water, then dried at 60 °C for later use.

Preparation of MTV-ZIF-AB, -AC, -AD and -AE membranes. All membranes were fabricated by a low-temperature convection-diffusion method with minor modifications to a reported procedure.[2, 4] Briefly, the PANI/PES composite substrate was immersed in 30 mL of a 0.02 M methanolic solution of zinc nitrate hexahydrate for 1 h and then dried at room temperature. The substrate was mounted vertically at the center of a custom two-cell setup, with the tight side of the substrate facing the zinc-ion solution cell and the other side facing the linker solution cell. Equal volumes of the zinc-ion solution and the linker solution were added to the opposing cells, and diffusion growth proceeded in situ at 5 °C for ~24 h (Figure S1). The resulting membrane was dried at room temperature for another 24 h. The effective diameter of the resulting membranes was approximately 3.5 cm.

The reaction solutions were prepared as follows: the zinc-ion solution was obtained by dissolving zinc nitrate hexahydrate (1.3 mmol) in methanol (100 mL). Ligand solutions were prepared by dissolving the desired proportions of linkers A-E (Mim, Mbim, Fmbim, Clmbim, Brmbim) in methanol (100 mL). Detailed compositions and

fabrication parameters are listed in Tables S1-S4.

Molecular simulation. The 2×2×2 supercell (33.98 Å × 33.98 Å × 33.98 Å) of optimized bulk ZIF-8 with 192 methylimidazole (Mim) was used to explore the halogen-substituted Mbim (methylbenzimidazole) effect on the micropore size. The Mim was substituted by Mbim and the halogen-substituted Mbim was constructed by substituting one H atom of benzene of the Mbim with F, Cl, Br atom, respectively. Its corresponding name and concentration were Mbim (43%), Fmbim (40%), Clmbim (28%) and Brmbim (33%). The concentration is defined as the ratio of halogen-substituted Mbim to the total number of methylimidazoles in the system.

After the anneal dynamics at 800K of 50 ps and geometry optimization, all pore size distribution (PSD) calculations were simulated at 300 K using the Grand Canonical Monte Carlo (GCMC) method with the RASPA software,[5] in which the force field origins from the combination of the Universal force field (UFF)[6] and the Dreiding force field,[7] where the metal center (Zn) is treated by UFF and other atoms by Dreiding force field. The CH_4 molecule was placed at different sites on Mim, Mbim, and halogen-substituted Mbim. Through rigorous screening, the most stable adsorption energy structure was identified. The adsorption energy (Eads) was calculated using the formula: Eads = $E_{(site+CH4)}$ - $E_{(site)}$ - $E_{(CH4)}$. All simulated X-ray Diffraction (XRD) were calculated by LAMMPS software.[8] Interactions were modelled using a Lennard-Jones potential (truncated at 12 with long-range tail corrections) and point-charge electrostatics (treated with the Ewald summation) between composite and gas molecules (He). The precision of Ewald is 10^{-6}. Cross-atomic force field parameters were defined using Lorentz–Berthelot mixing rule. Each simulations had 10^4 steps. The visualization of pores was implemented by OVITO.[9] For electrostatic potential (ESP), the Hartree-Fock method with 6-311g basis set was conducted in Beijing Density Functional (BDF)[10] and the Multiwfn software[11-12] and the VMD[13] were used to color the van der Waals surface for all monomers (Figure S46). The diffusion coefficient of CH_4 was obtained by calculating the mean square displacement (MSD) when the system reached equilibrium. The diffusion coefficients reached a stable value when the

simulation time reached 50 ns. To characterize the inter-cage transport mechanism, the selected diffusion path for He and CH_4 hopping through the edited pore-apertures was simulated in the various MOFs via MD. The simulated pathway, extending approximately 20 Å in length, traverses multiple adjacent cages that are connected by six-membered pore-window.

Techno-economic analysis method. The membrane and cryogenic separation processes were modeled under steady-state conditions using Aspen Plus V14.[14-17] The NRTL model was employed for property calculations, and the RadFrac module was used to simulate cryogenic distillation columns. For the cryogenic distillation process, each column was operated at constant pressure with a fixed number of 10 theoretical trays. A four-stage distillation sequence was assessed to achieve ultra-high-purity helium (>99.999%) from a feed stream containing 0.6% He and 99.4% CH_4 at 25 °C, for a volume of 100,000 m^3 $year^{-1}$. The condenser and heat exchanger outlet temperatures were set to -157 °C and -88 °C, respectively. A cold-recovery unit (multi-stream heat exchanger) was installed upstream to precool the feed and reduce condenser and reboiler duties.

For the membrane-based separation process, the first membrane stage treated a feed containing ~0.6 mol% He and delivered a permeate with ~98% He. A second-stage membrane further purified the product stream to 99.999% He. The separation factor was derived from the experimental He/CH_4 mixed-gas selectivity of 418. In the main process flow, the feed gas was compressed to 0.5 MPa, cooled to ~25 °C, and introduced into the first membrane module. The permeate from the first stage was recompressed and recooled under similar conditions before entering the second-stage membrane.

Economic analysis was carried out using the Aspen Economic Evaluator tool with built-in templates and database factors. The total costs included equipment and utility costs. Equipment costs comprised the purchased and installed expenses of the distillation columns and heat exchangers, while utility costs were based on steam and refrigerant usage. Total energy duty was calculated by summing the condenser and reboiler duties.

Notably, the potential revenue from the sale of purified helium was excluded from the economic analysis, although such profits could substantially reduce the actual cost of helium recovery in industrial applications. Meanwhile, we therefore do not credit (or co-allocate) potential additional value from “cold energy” or liquid natural gas (LNG) co-production in the cryogenic case. Because effective cold-energy utilization often requires additional capital equipment and site-specific integration, we do not include these credits at this stage.

2. Supporting figures

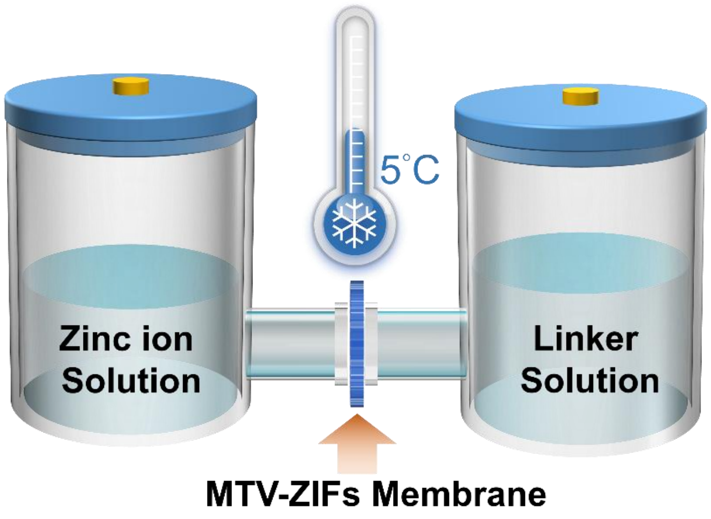

Figure S1. Schematic diagram of the device used for preparing MTV-ZIF membranes based on the low-temperature convection-diffusion method.

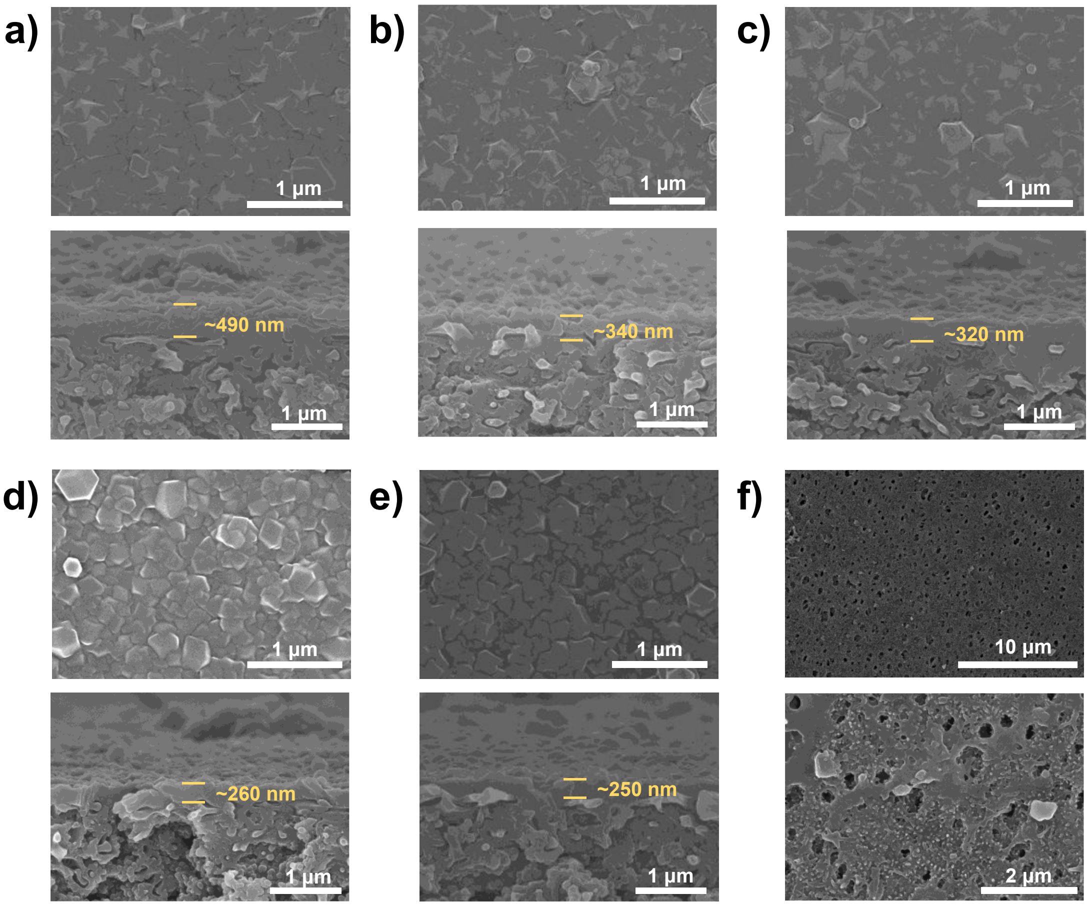

Figure S2. SEM images of MTV-ZIF-A$_{(100-x)}$B$_x$ membranes. a-e) Surface (top) and cross-sectional (bottom) SEM images of MTV-ZIF-A$_{90}$B$_{10}$ (a), MTV-ZIF-A$_{82}$B$_{18}$ (b), MTV-ZIF-A$_{71}$B$_{29}$ (c), MTV-ZIF-A$_{59}$B$_{41}$ (d), and MTV-ZIF-A$_{57}$B$_{43}$ (e) membranes. f) Top-view of MTV-ZIF-A$_{50}$B$_{50}$ membrane with low magnification (top) and high magnification (bottom).

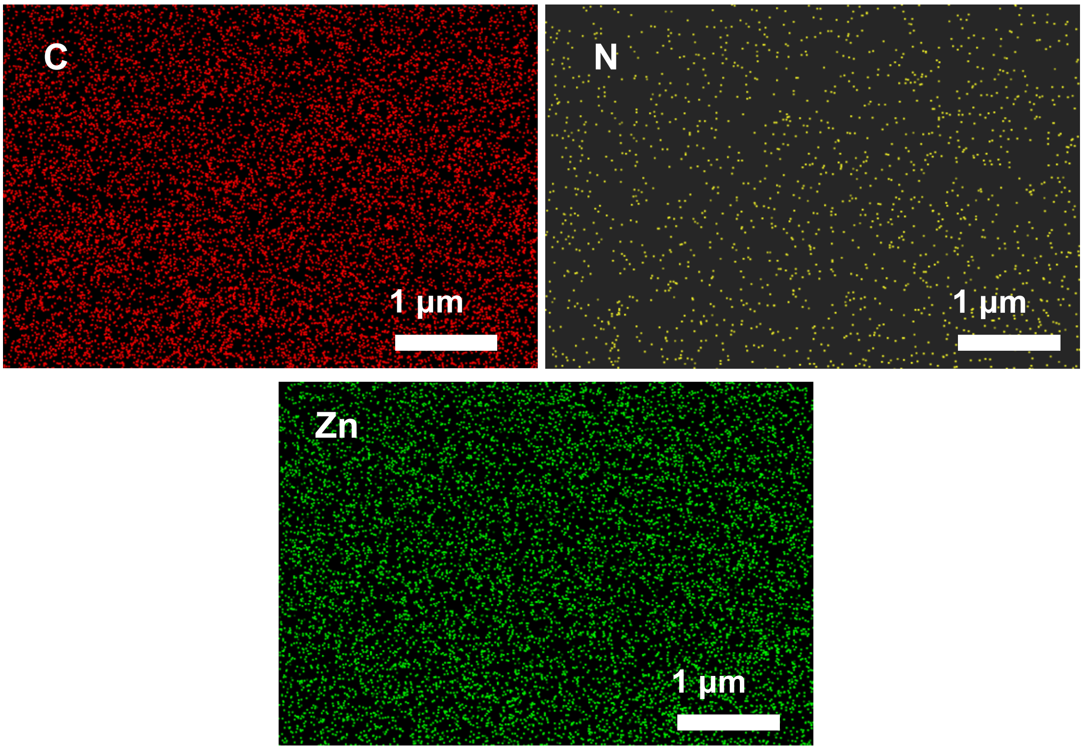

Figure S3. Surface EDS maps of MTV-ZIF-$A_{57}B_{43}$ membrane.

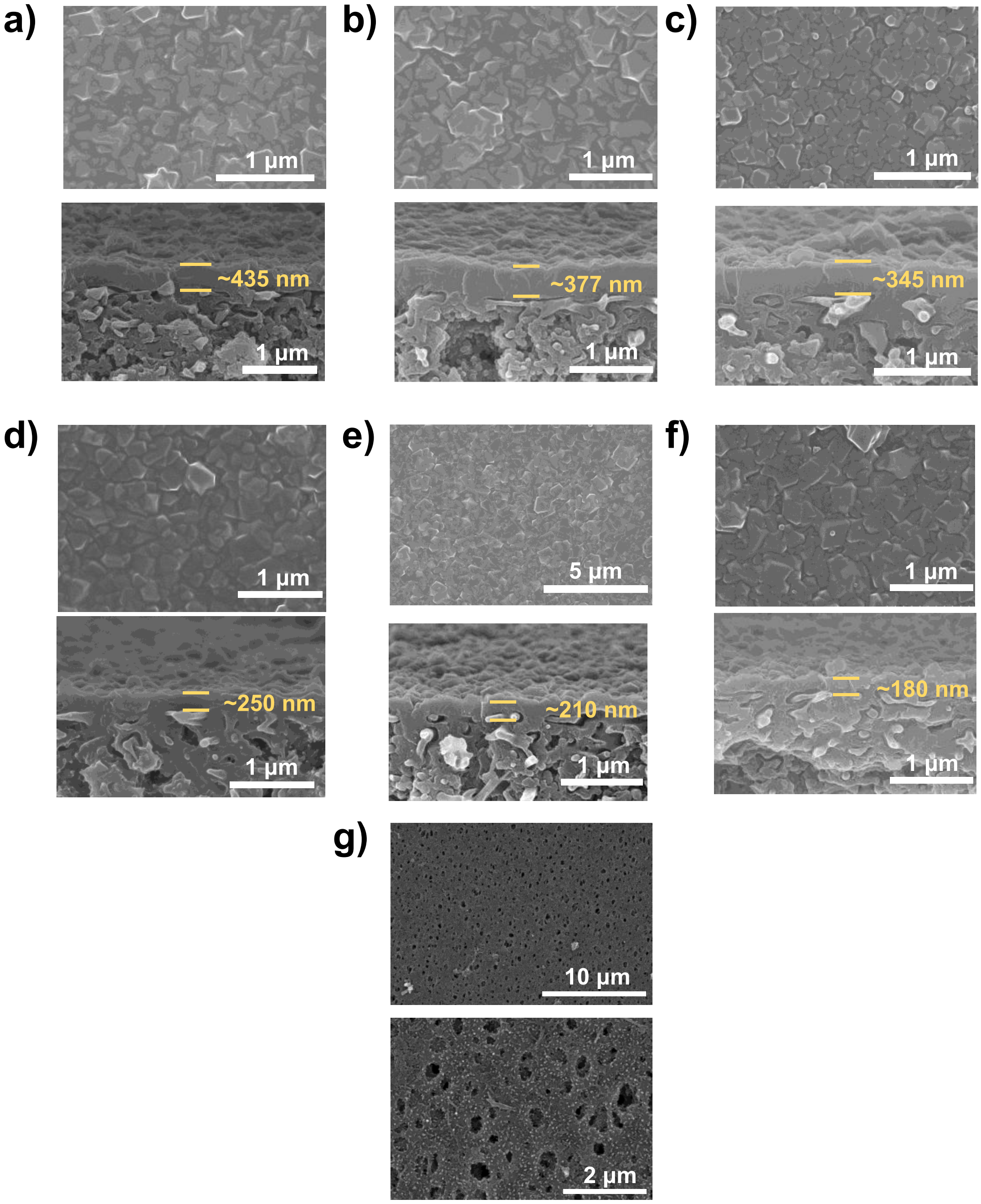

Figure S4. SEM images of MTV-ZIF-$A_{(100-x)}C_x$ membranes. a-c) Surface (top) and cross-sectional (bottom) SEM images of MTV-ZIF-$A_{90}C_{10}$ (a), MTV-ZIF-$A_{82}C_{18}$ (b), MTV-ZIF-$A_{70}C_{30}$ (c), MTV-ZIF-$A_{66}C_{34}$ (d), MTV-ZIF-$A_{60}C_{40}$ (e) and MTV-ZIF-$A_{55}C_{45}$ (f) membranes. g) Top-view of MTV-ZIF-$A_{50}C_{50}$ membrane with low magnification (top) and high magnification (bottom).

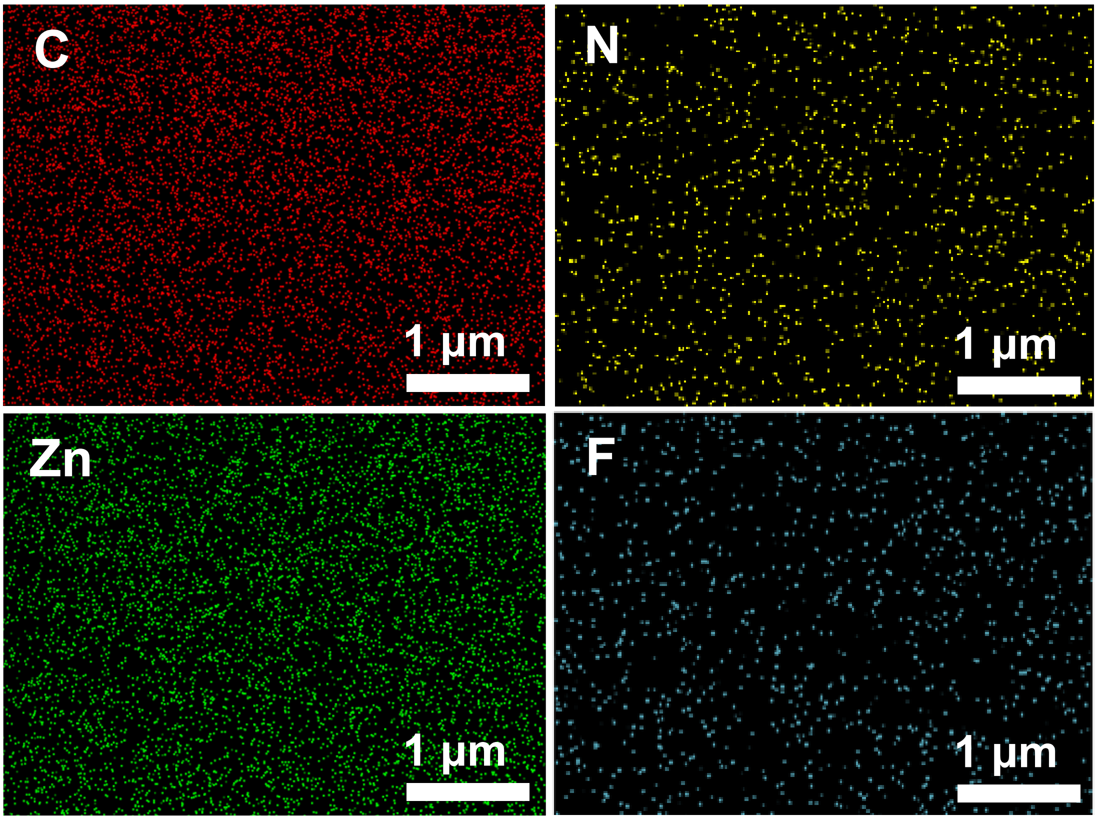

Figure S5. Surface EDS maps of MTV-ZIF-$A_{60}C_{40}$ membrane.

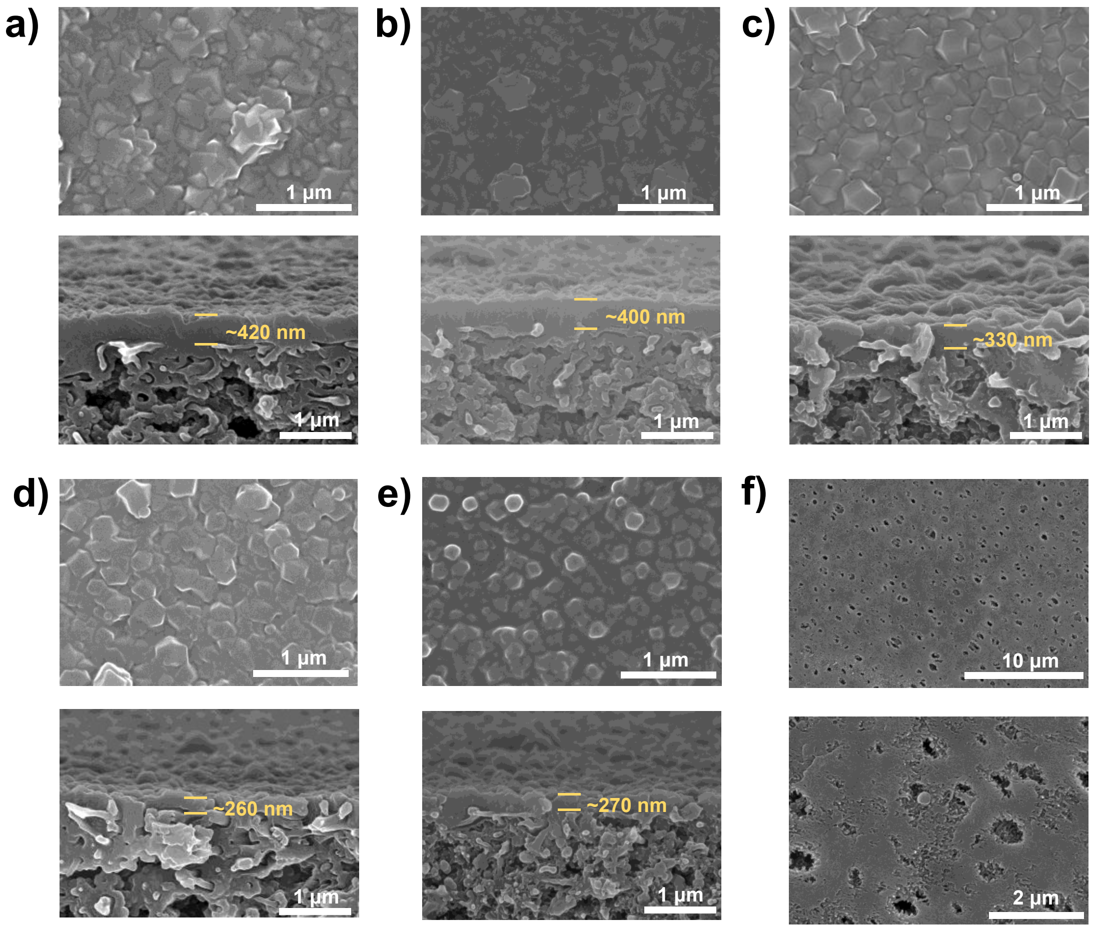

Figure S6. SEM images of MTV-ZIF-$A_{(100-x)}D_x$ membranes. a-e) Surface (top) and cross-sectional (bottom) SEM images of MTV-ZIF-$A_{92}D_8$ (a), MTV-ZIF-$A_{85}D_{15}$ (b), MTV-ZIF-$A_{77}D_{23}$ (c), MTV-ZIF-$A_{72}D_{28}$ (d) and MTV-ZIF-$A_{65}D_{35}$ (e) membranes. f) Top-view of MTV-ZIF-$A_{60}D_{40}$ membrane with low magnification (top) and high magnification (bottom).

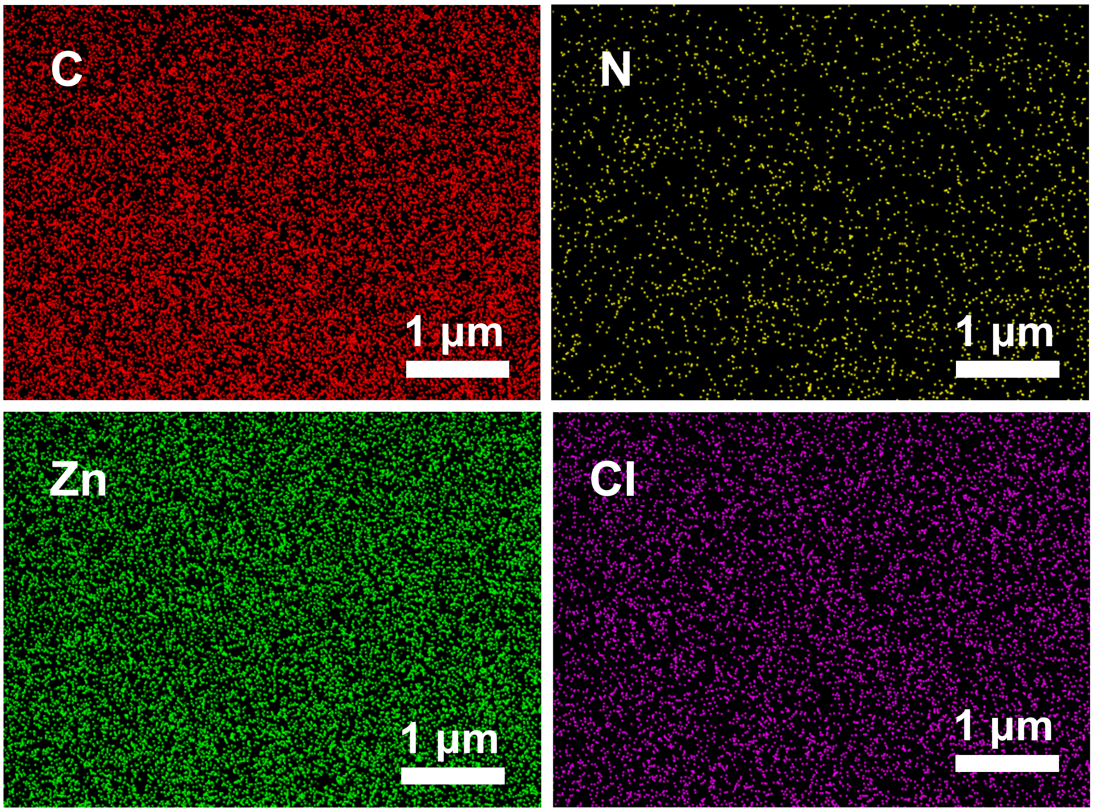

Figure S7. Surface EDS maps of MTV-ZIF-$A_{72}D_{28}$ membrane.

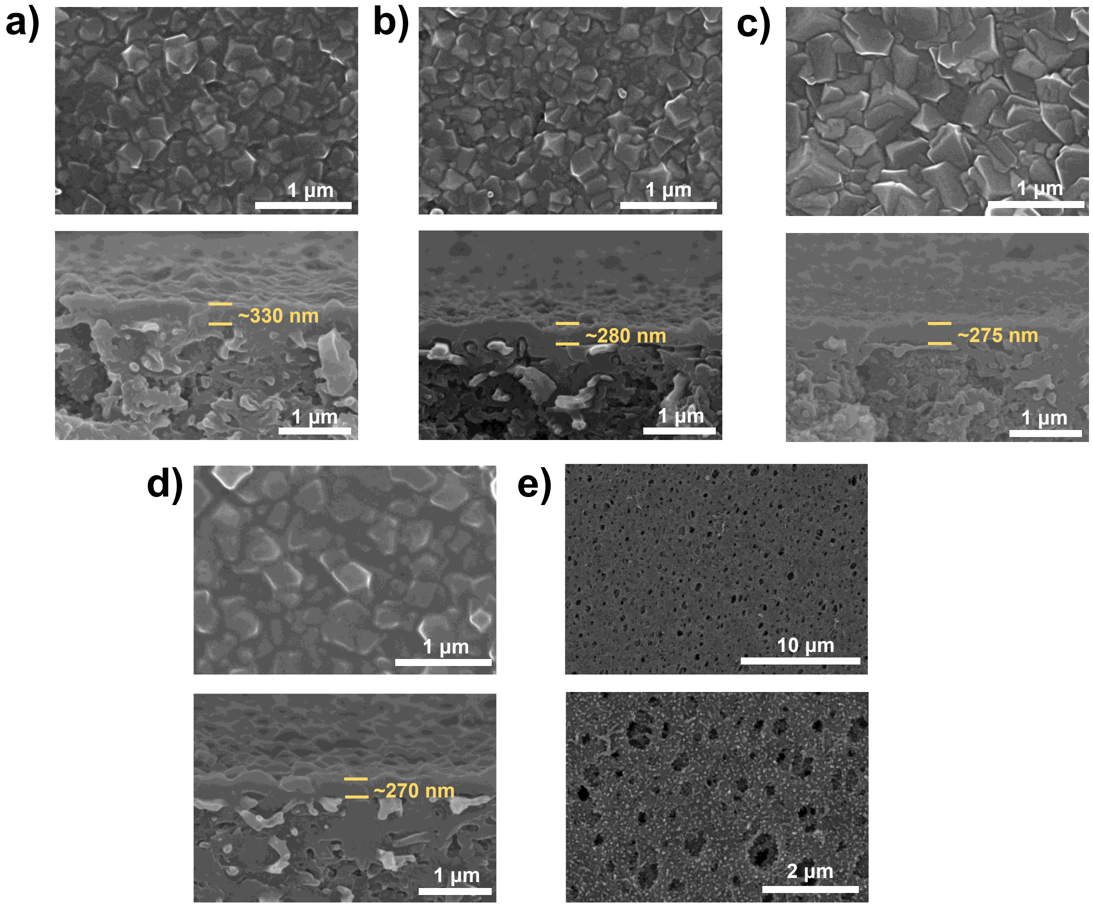

Figure S8. SEM images of MTV-ZIF-A$_{(100-x)}$E$_x$ membranes. a-d) Surface (top) and cross-sectional (bottom) SEM images of MTV-ZIF-A$_{90}$E$_{10}$ (a), MTV-ZIF-A$_{86}$E$_{14}$ (b), MTV-ZIF-A$_{76}$E$_{24}$ (c) and MTV-ZIF-A$_{67}$E$_{33}$ (d) membranes. e) Top-view of MTV-ZIF-A$_{60}$E$_{40}$ membrane with low magnification (top) and high magnification (bottom).

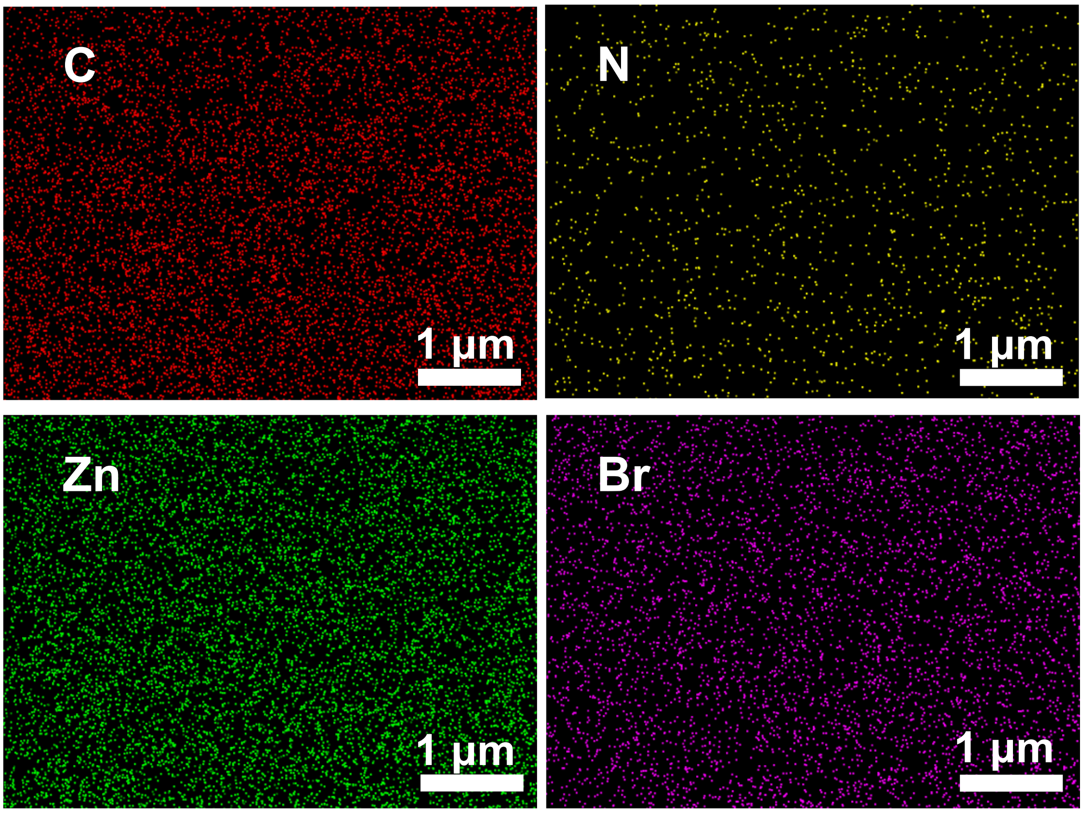

Figure S9. Surface EDS maps of MTV-ZIF-$A_{67}E_{33}$ membrane.

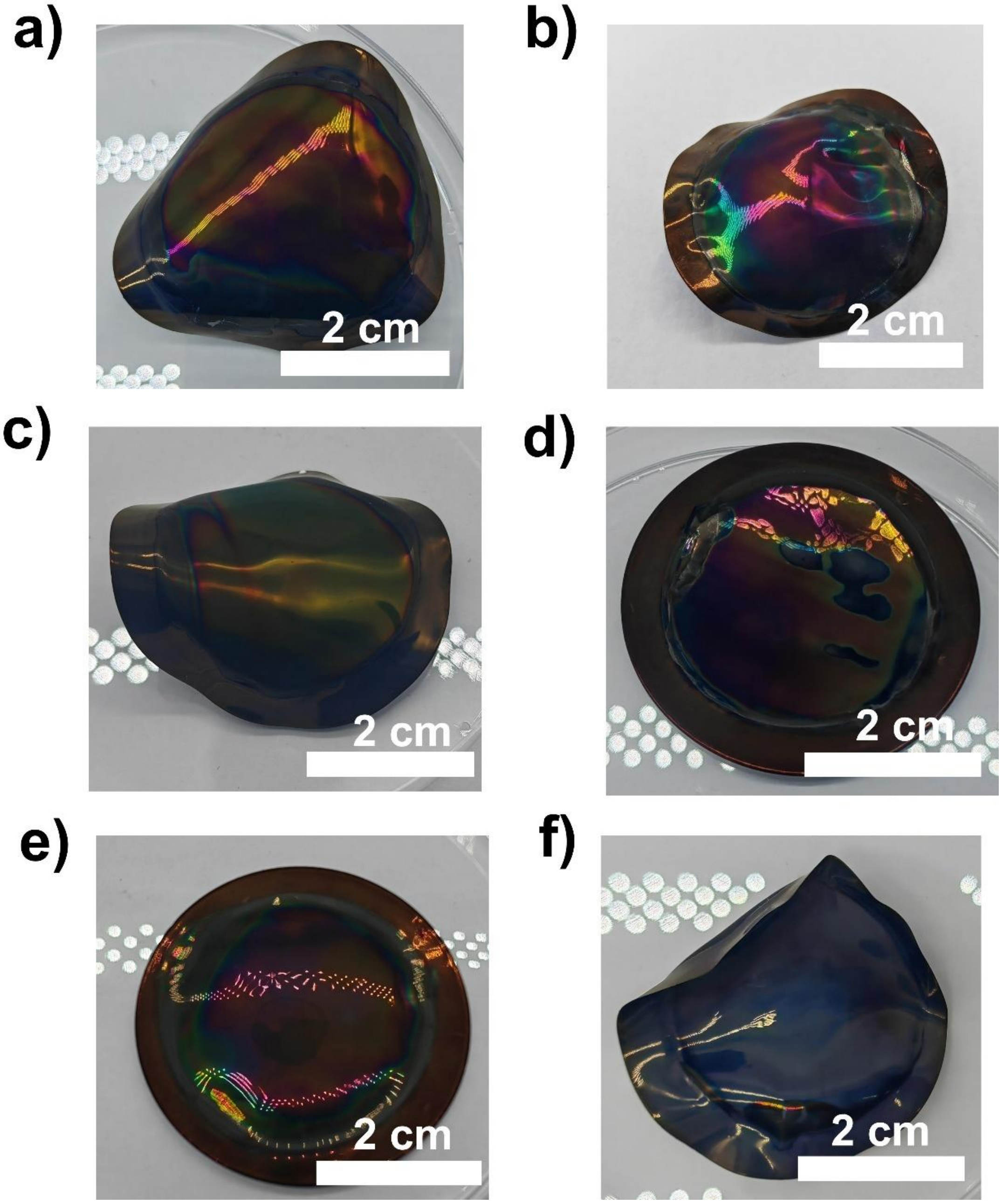

Figure S10. Optical photos of MTV-ZIF-A$_{(100\text{-}x)}$B$_x$ membranes. MTV-ZIF-$A_{90}B_{10}$ (a), MTV-ZIF-$A_{82}B_{18}$ (b), MTV-ZIF-$A_{71}B_{29}$ (c), MTV-ZIF-$A_{59}B_{41}$ (d), MTV-ZIF-$A_{57}B_{43}$ (e) and MTV-ZIF-$A_{50}B_{50}$ (f) membranes.

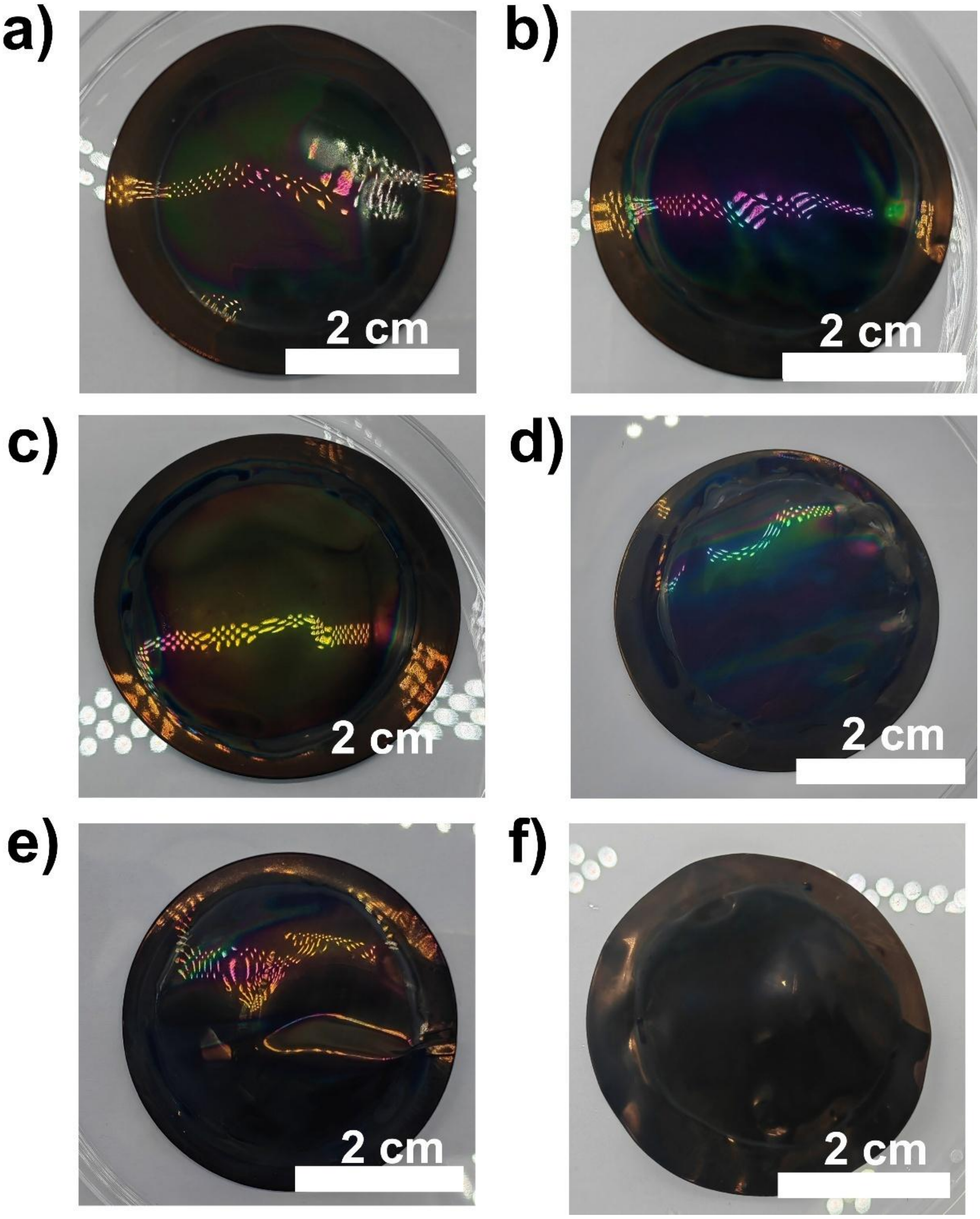

Figure S11. Optical photos of MTV-ZIF-A$_{(100-x)}$C$_x$ membranes. MTV-ZIF-A$_{90}$C$_{10}$ (a), MTV-ZIF-A$_{82}$C$_{18}$ (b), MTV-ZIF-A$_{70}$C$_{30}$ (c), MTV-ZIF-A$_{66}$C$_{34}$ (d), MTV-ZIF-A$_{55}$C$_{45}$ (e) and MTV-ZIF-A$_{50}$C$_{50}$ (f) membranes.

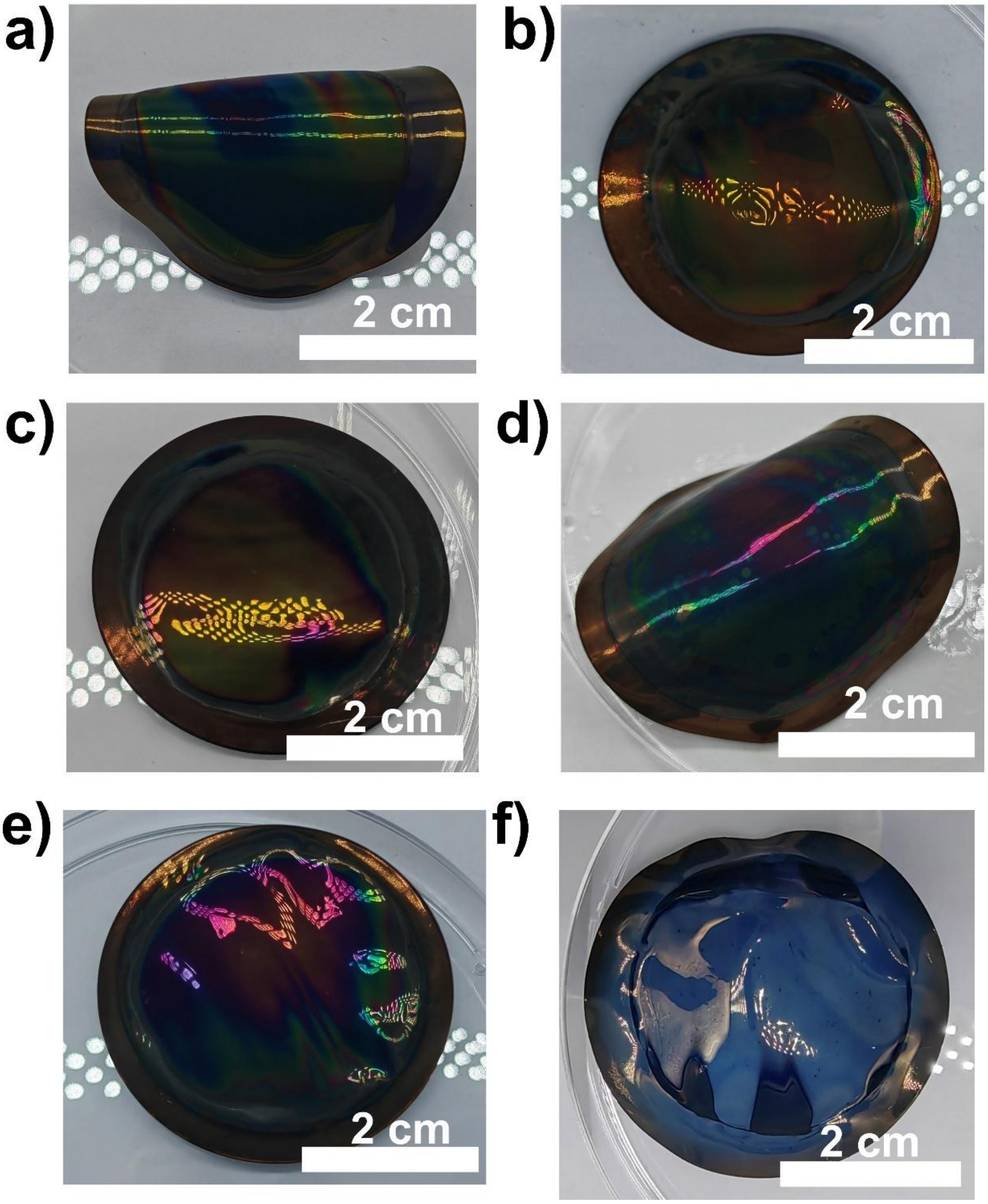

Figure S12. Optical photos of MTV-ZIF-A$_{(100-x)}$D$_x$ membranes. MTV-ZIF-A$_{92}$D$_8$ (a), MTV-ZIF-A$_{85}$D$_{15}$ (b), MTV-ZIF-A$_{77}$D$_{23}$ (c), MTV-ZIF-A$_{72}$D$_{28}$ (d), MTV-ZIF-A$_{65}$D$_{35}$ (e) and MTV-ZIF-A$_{60}$D$_{40}$ (f) membranes.

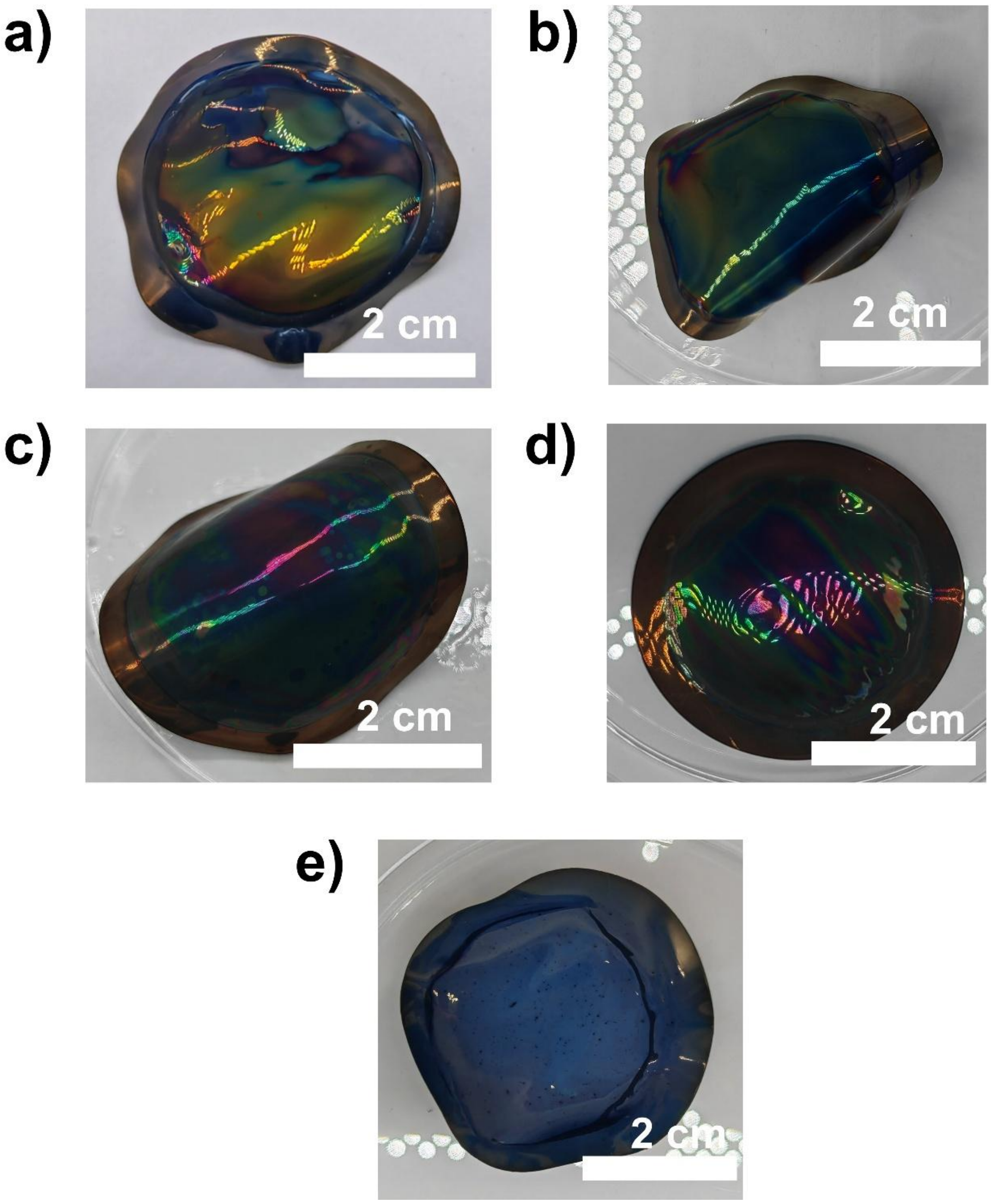

Figure S13. Optical photos of MTV-ZIF-$A_{(100-x)}E_x$ membranes. MTV-ZIF-$A_{90}E_{10}$ (a), MTV-ZIF-$A_{86}E_{14}$ (b), MTV-ZIF-$A_{76}E_{24}$ (c), MTV-ZIF-$A_{67}E_{33}$ (d) and MTV-ZIF-$A_{60}E_{40}$ (e) membranes.

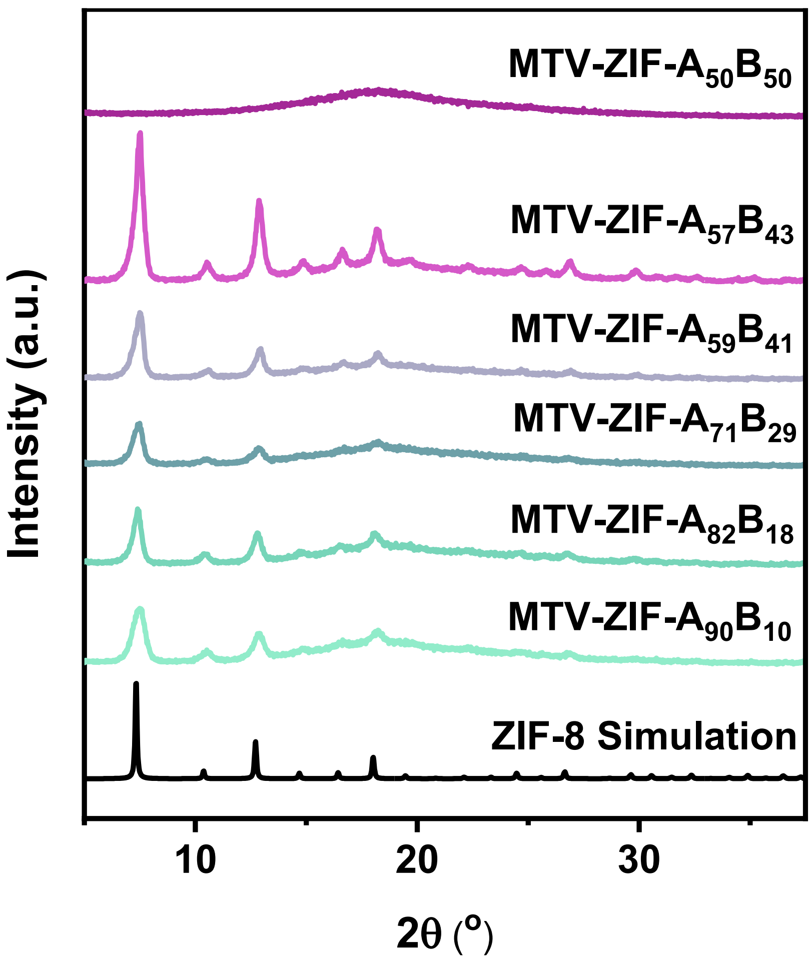

Figure S14. PXRD patterns of MTV-ZIF-$A_{(100-x)}B_x$ membranes.

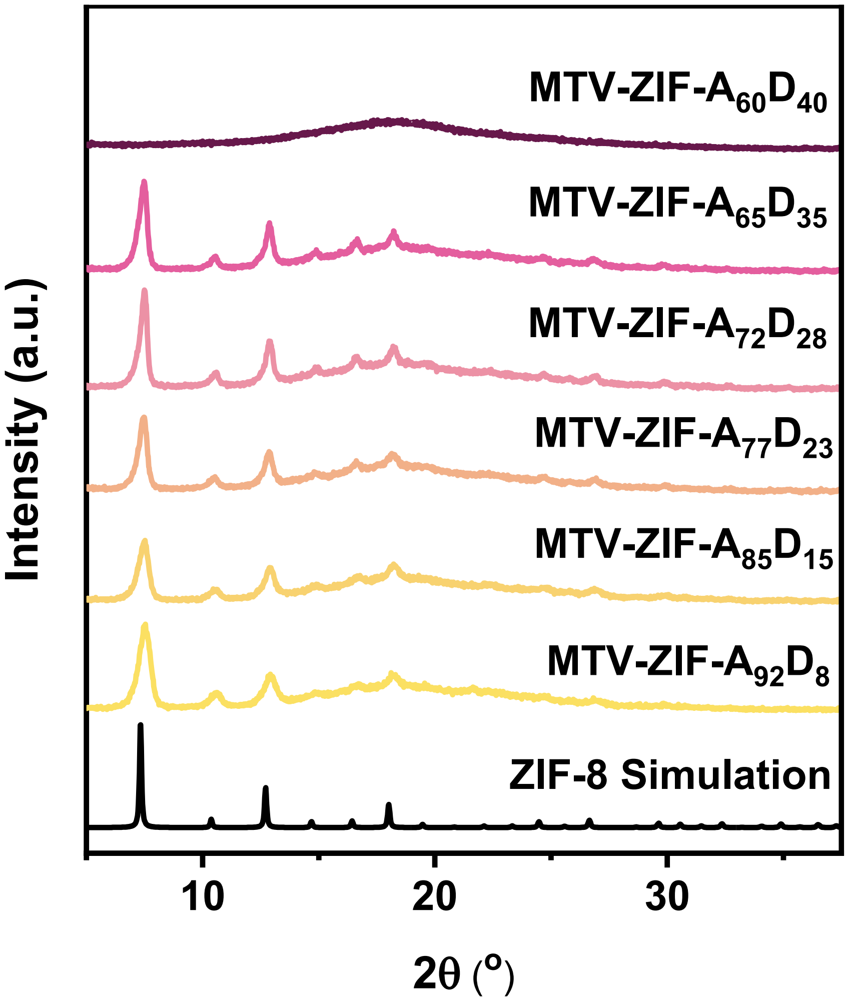

Figure S15. PXRD patterns of MTV-ZIF-$A_{(100-x)}D_x$ membranes.

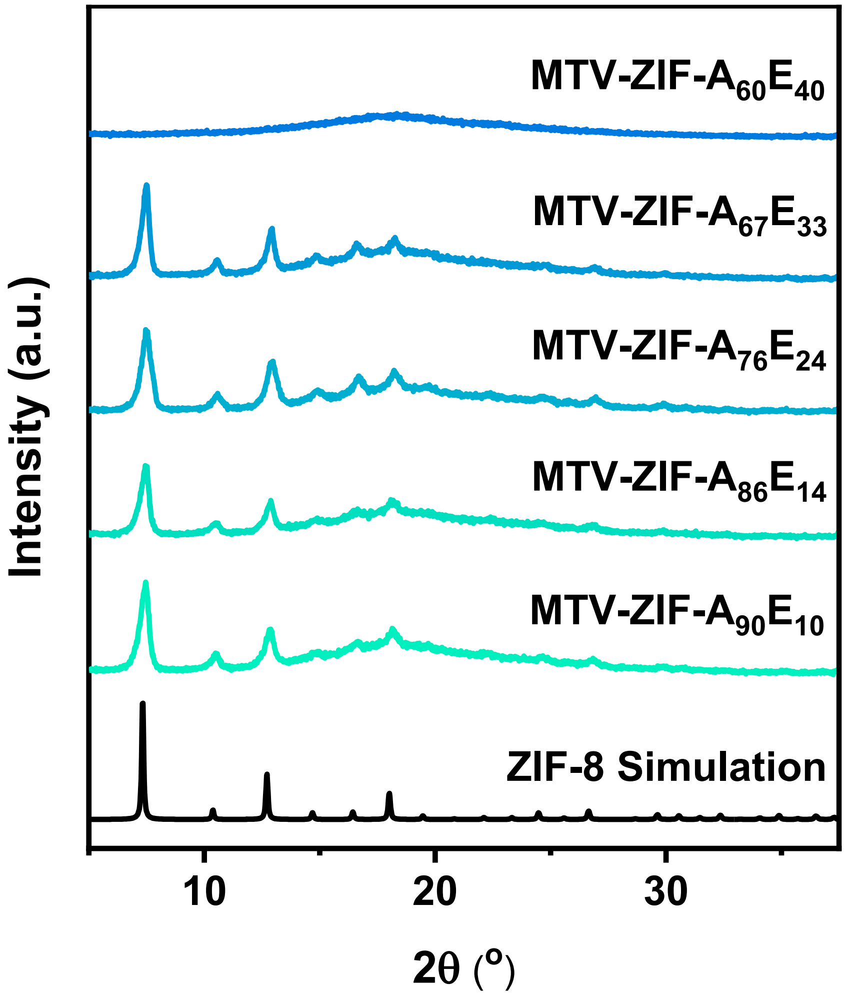

Figure S16. PXRD patterns of MTV-ZIF-A$_{(100\text{-}x)}$E$_x$ membranes.

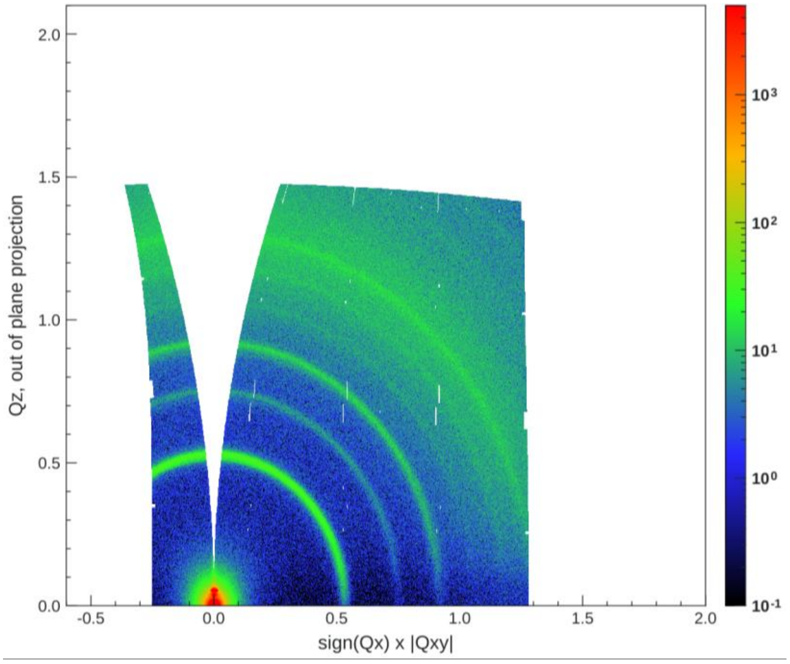

Figure S17. GIWAX pattern of ZIF-8 membrane.

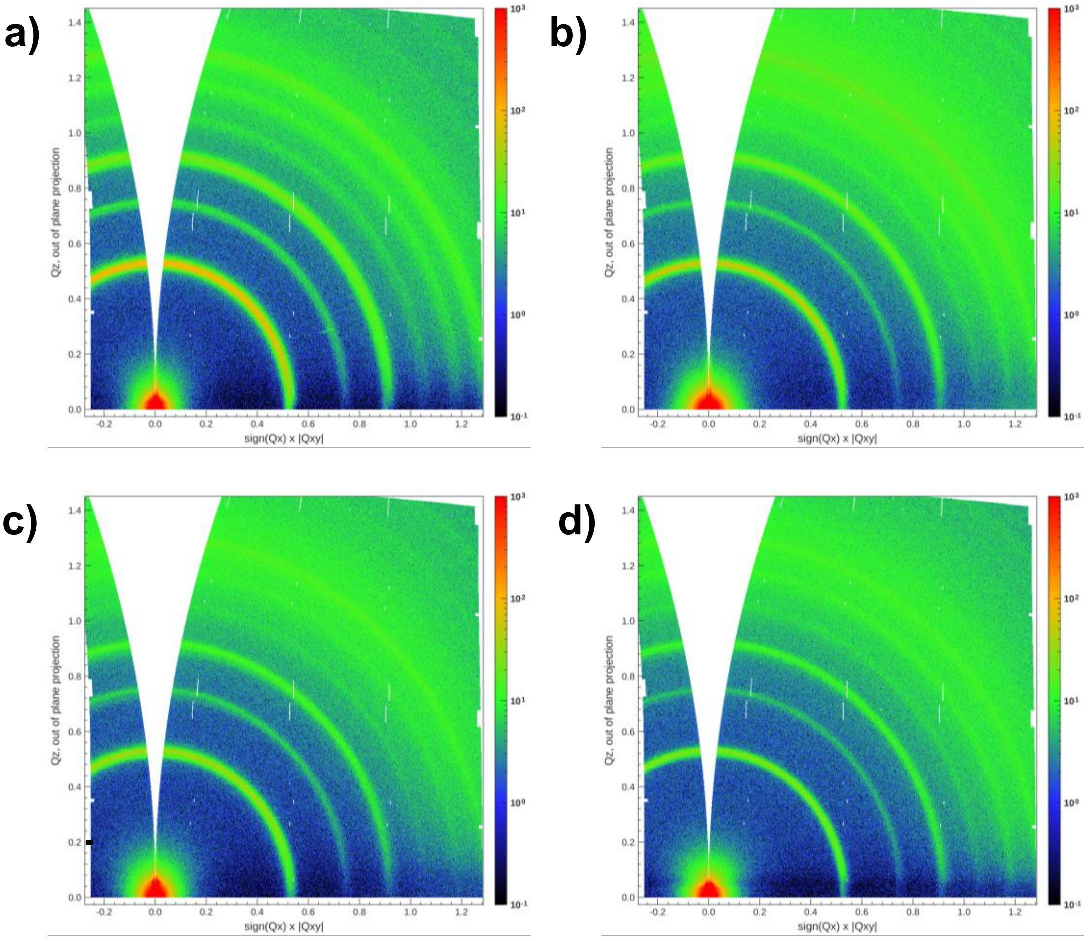

Figure S18. GIWAX patterns of a) MTV-ZIF-$A_{57}B_{43}$, b) MTV-ZIF-$A_{60}C_{40}$, c) MTV-ZIF-$A_{72}D_{28}$ and d) MTV-ZIF-$A_{67}E_{33}$ membranes.

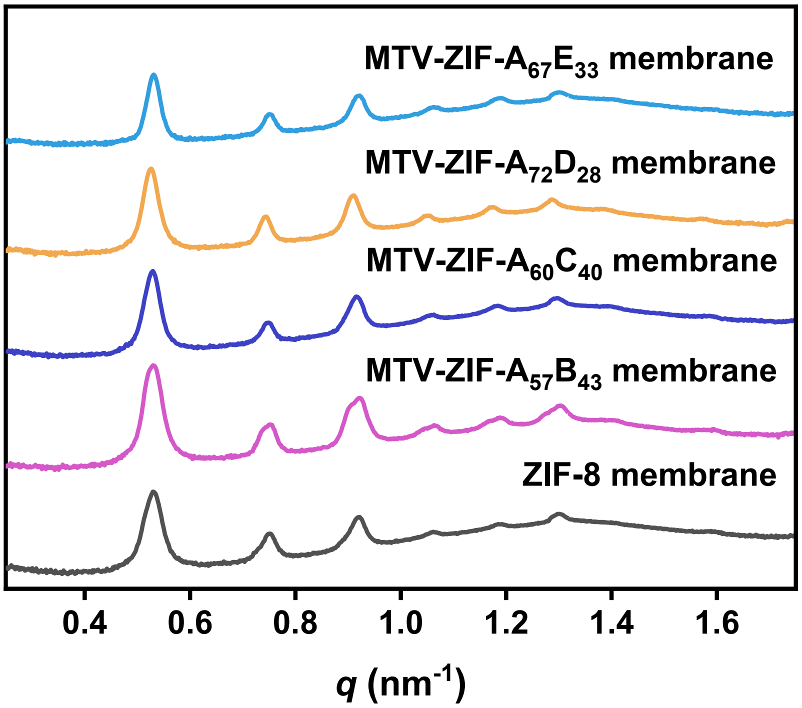

Figure S19. GIWAX diffraction intensity profiles of ZIF-8, MTV-ZIF-$A_{57}B_{43}$, MTV-ZIF-$A_{60}C_{40}$, MTV-ZIF-$A_{72}D_{28}$, and MTV-ZIF-$A_{67}E_{33}$ membranes.

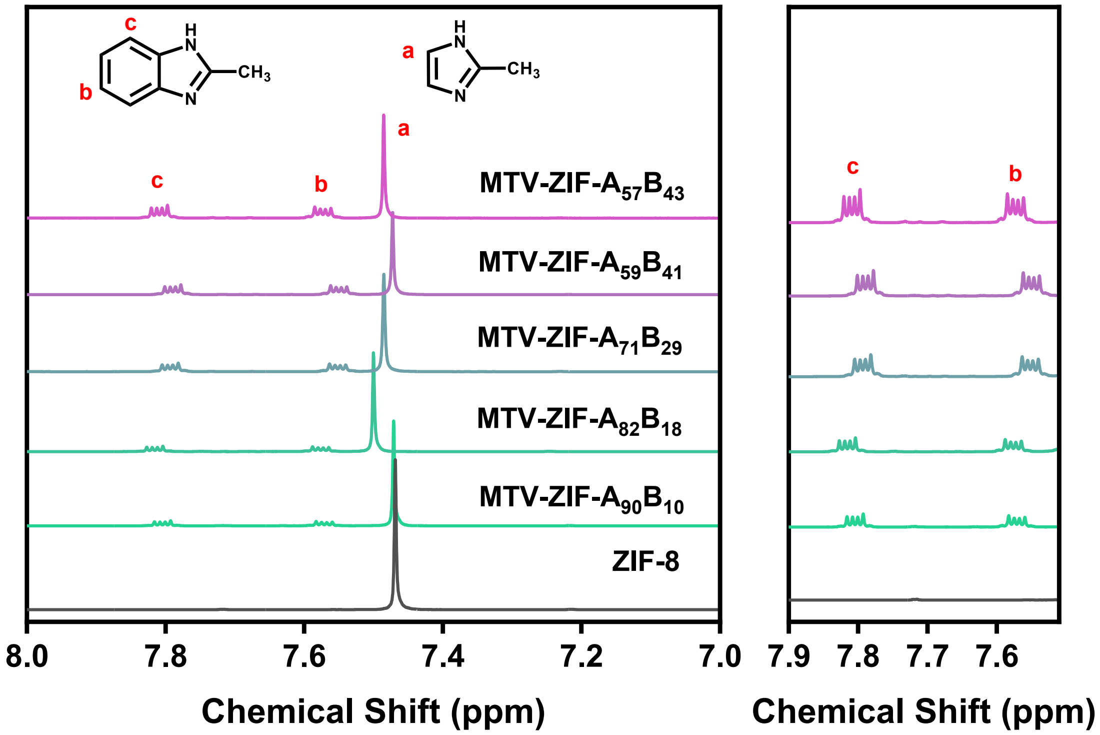

Figure S20. ^{1}H NMR spectra of acid-digested MTV-ZIF-$A_{(100-x)}B_x$ membranes.

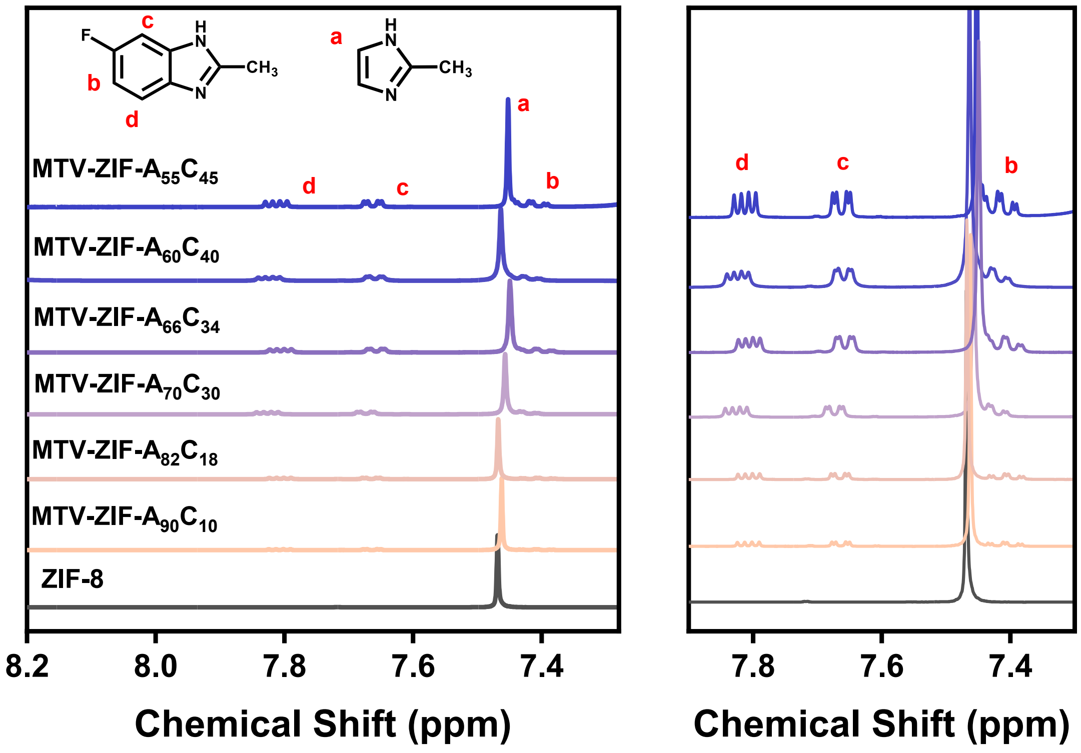

Figure S21. ^{1}H NMR spectra of acid-digested MTV-ZIF-A$_{(100-x)}$C$_x$ membranes.

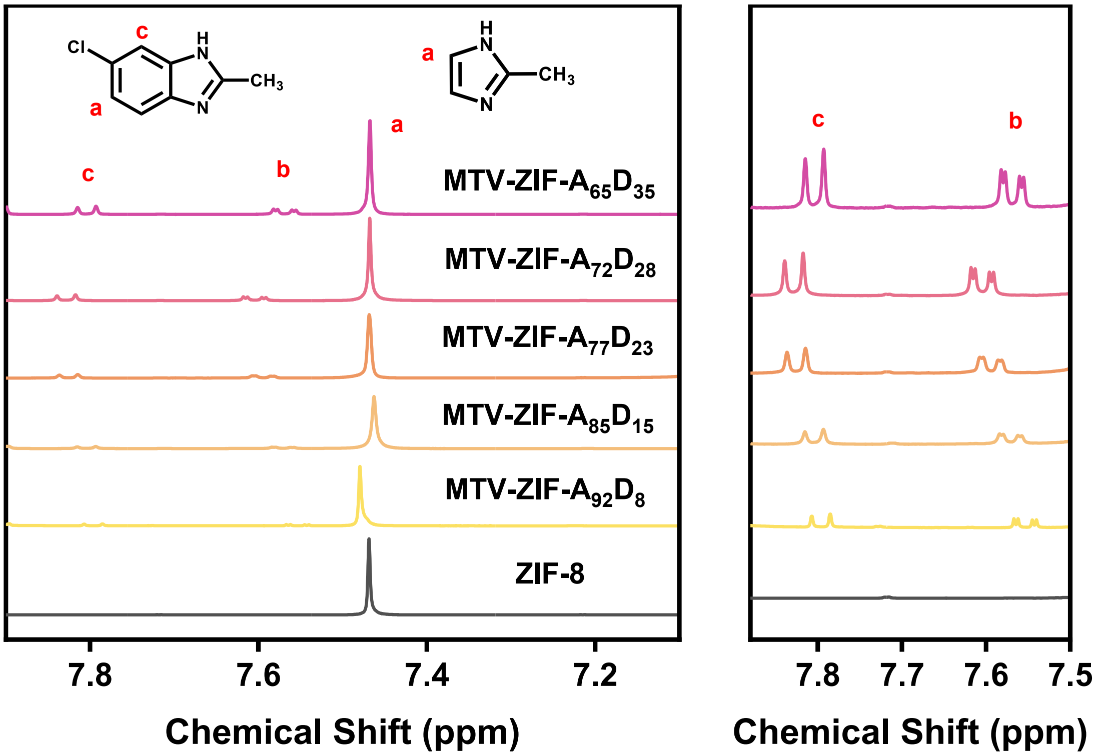

Figure S22. ^{1}H NMR spectra of acid-digested MTV-ZIF-A$_{(100\text{-}x)}$D$_x$ membranes.

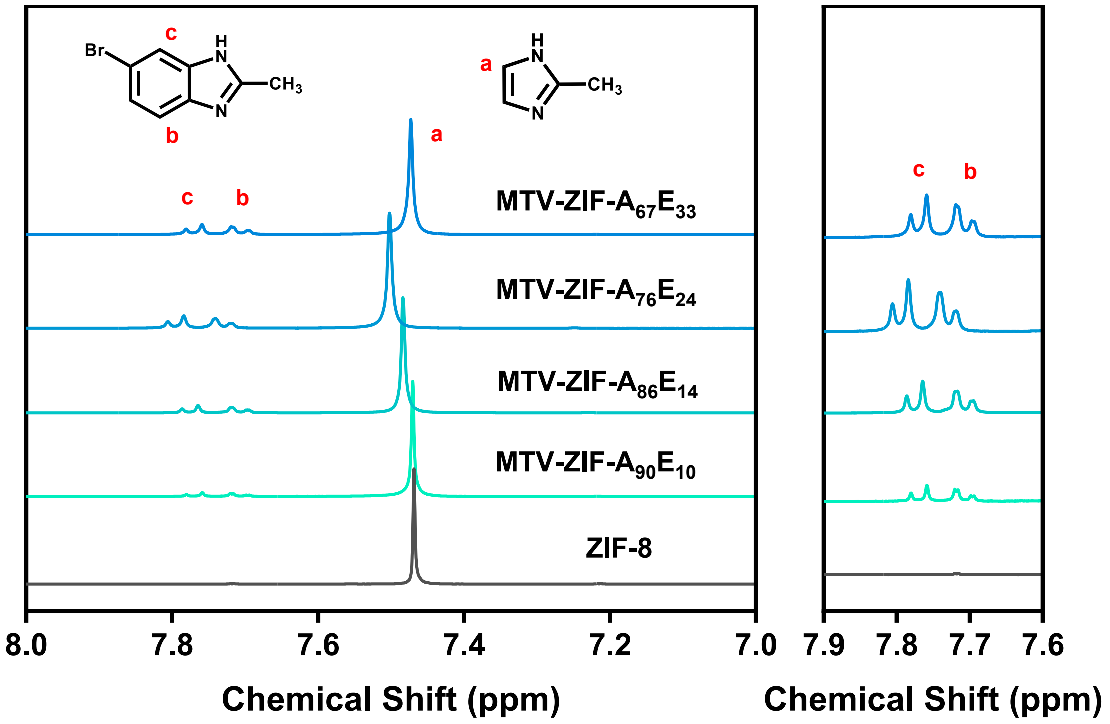

Figure S23. ^{1}H NMR spectra of acid-digested MTV-ZIF-A$_{(100\text{-}x)}$E$_x$ membranes.

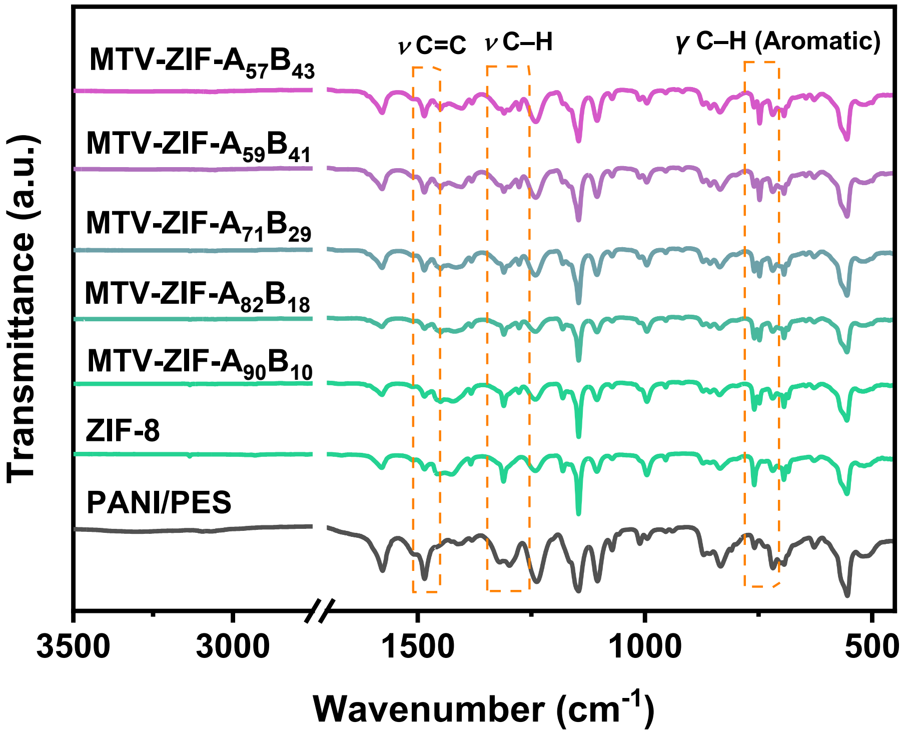

Figure S24. ATR-FTIR spectra of MTV-ZIF-$A_{(100-x)}B_x$ membranes and PANI/PES substrate.

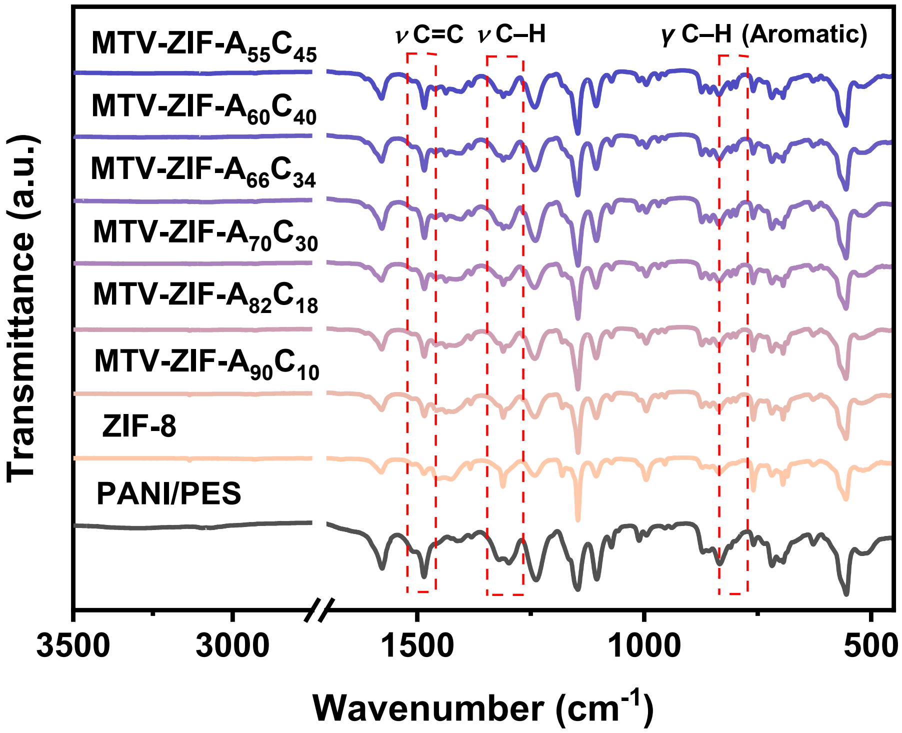

Figure S25. ATR-FTIR spectra of MTV-ZIF-$A_{(100-x)}C_x$ membranes and PANI/PES substrate.

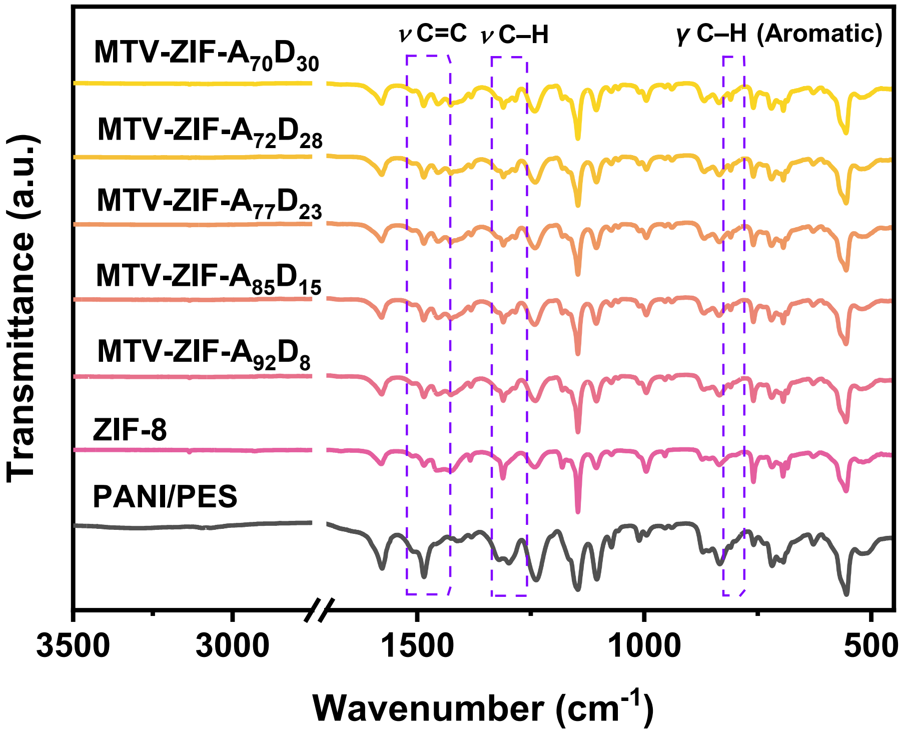

Figure S26. ATR-FTIR spectra of MTV-ZIF-$A_{(100\text{-}x)}D_x$ membranes and PANI/PES substrate.

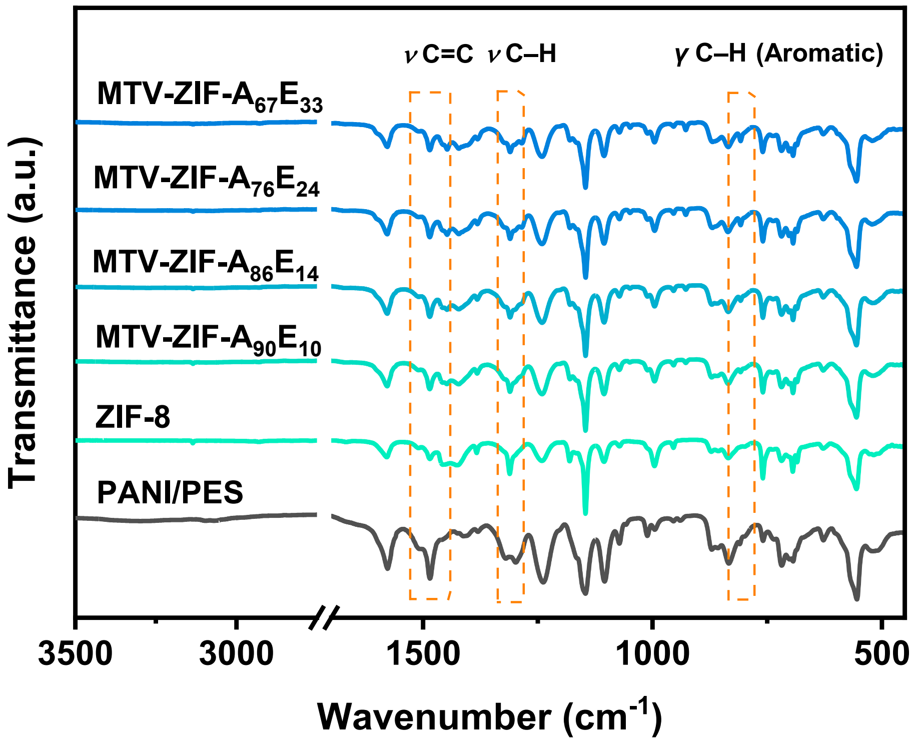

Figure S27. ATR-FTIR spectra of MTV-ZIF-$A_{(100\text{-}x)}E_x$ membranes and PANI/PES substrate.

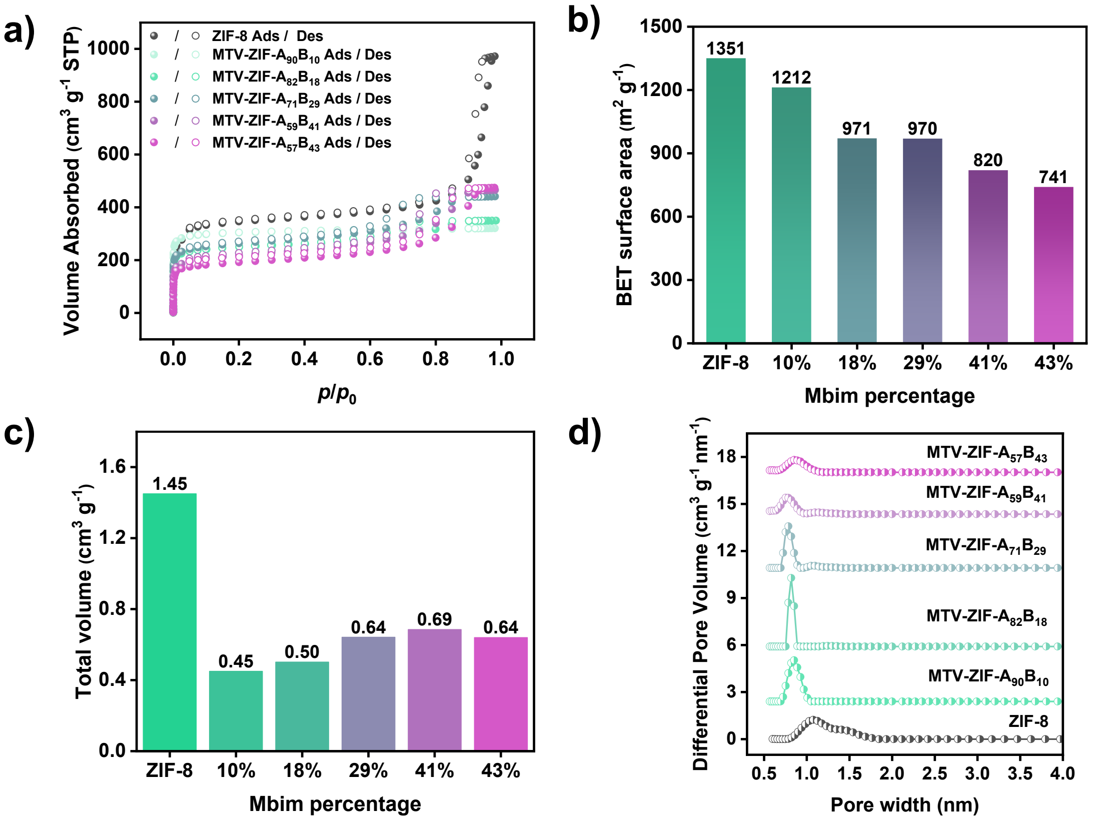

Figure S28. a) N_2 adsorption-desorption isotherms of ZIF-8 and MTV-ZIF-$A_{(100-x)}B_x$ measured at 77K; b) BET surface areas of ZIF-8 and MTV-ZIF-$A_{(100-x)}B_x$; c) Pore volume of ZIF-8 and MTV-ZIF-$A_{(100-x)}B_x$; d) Pore size distributions for ZIF-8 and MTV-ZIF-$A_{(100-x)}B_x$.

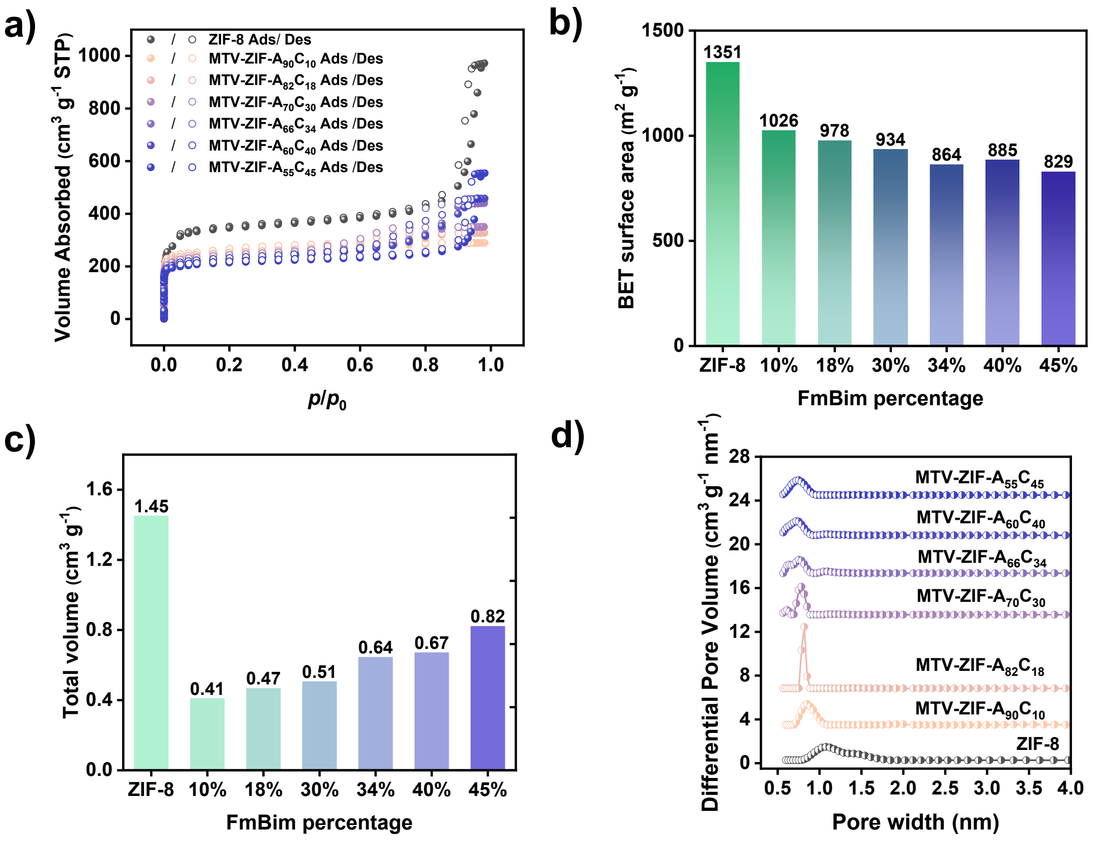

Figure S29. a) N_2 adsorption-desorption isotherms of ZIF-8 and MTV-ZIF-$A_{(100-x)}C_x$ measured at 77K; b) BET surface areas of ZIF-8 and MTV-ZIF-$A_{(100-x)}C_x$; c) Pore volume of ZIF-8 and MTV-ZIF-$A_{(100-x)}C_x$; d) Pore size distributions for ZIF-8 and MTV-ZIF-$A_{(100-x)}C_x$.

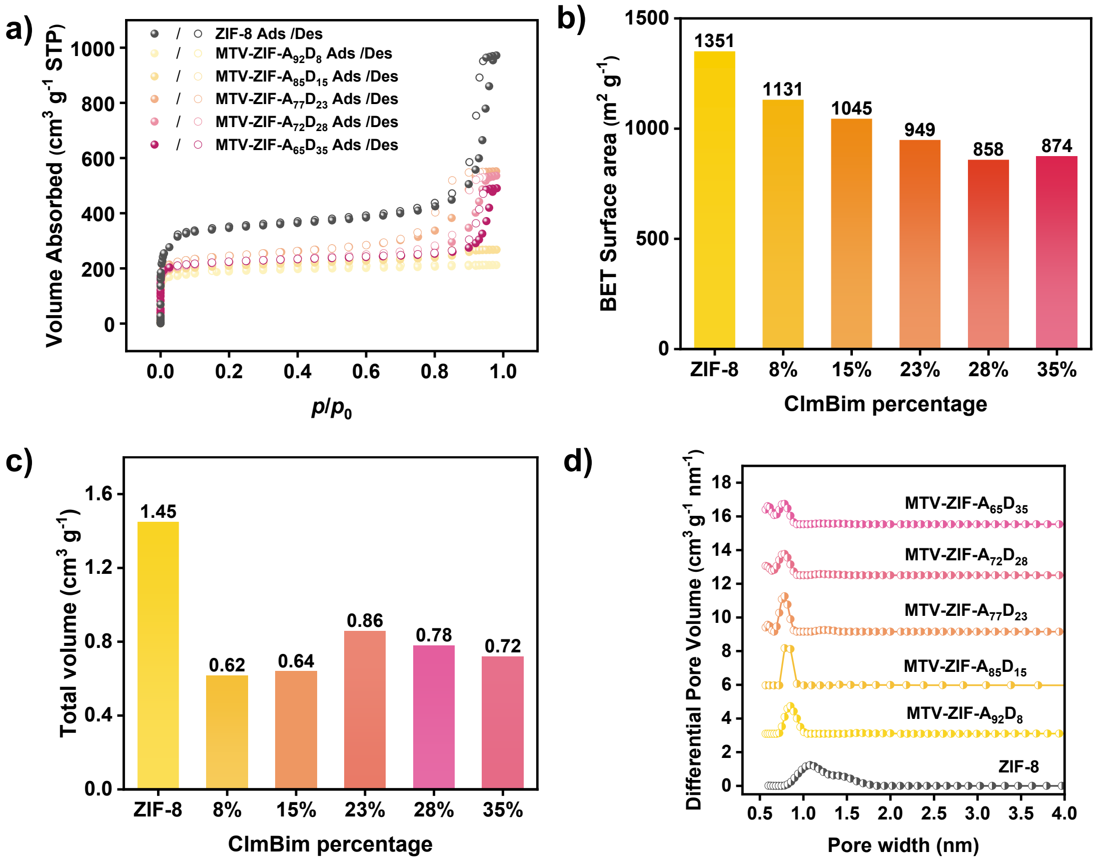

Figure S30. a) N_2 adsorption-desorption isotherms of ZIF-8 and MTV-ZIF-A$_{(100-x)}$D$_x$ measured at 77K; b) BET surface areas of ZIF-8 and MTV-ZIF-A$_{(100-x)}$D$_x$; c) Pore volume of ZIF-8 and MTV-ZIF-A$_{(100-x)}$D$_x$; d) Pore size distributions for ZIF-8 and MTV-ZIF-A$_{(100-x)}$D$_x$.

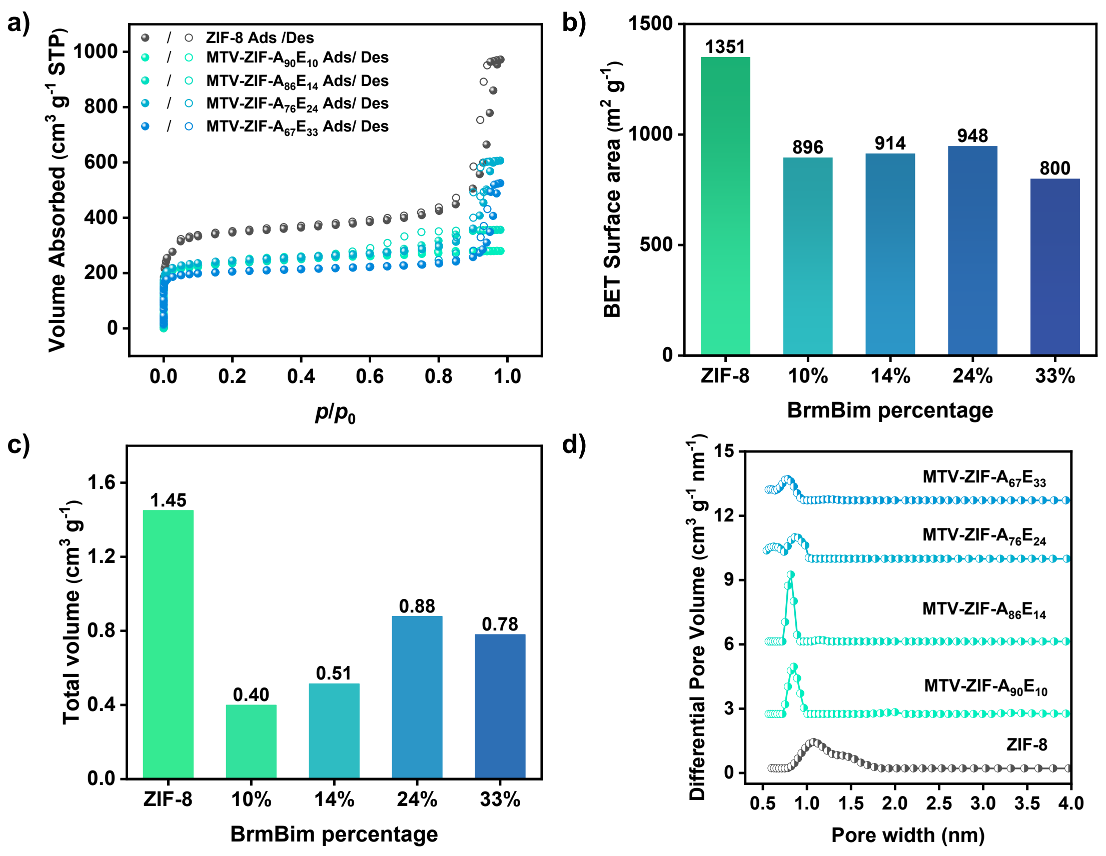

Figure S31. a) N_2 adsorption-desorption isotherms of ZIF-8 and MTV-ZIF-A$_{(100\text{-}x)}$E$_x$ measured at 77K; b) BET surface areas of ZIF-8 and MTV-ZIF-A$_{(100\text{-}x)}$E$_x$; c) Pore volume of ZIF-8 and MTV-ZIF-A$_{(100\text{-}x)}$E$_x$; d) Pore size distributions for ZIF-8 and MTV-ZIF-A$_{(100\text{-}x)}$E$_x$.

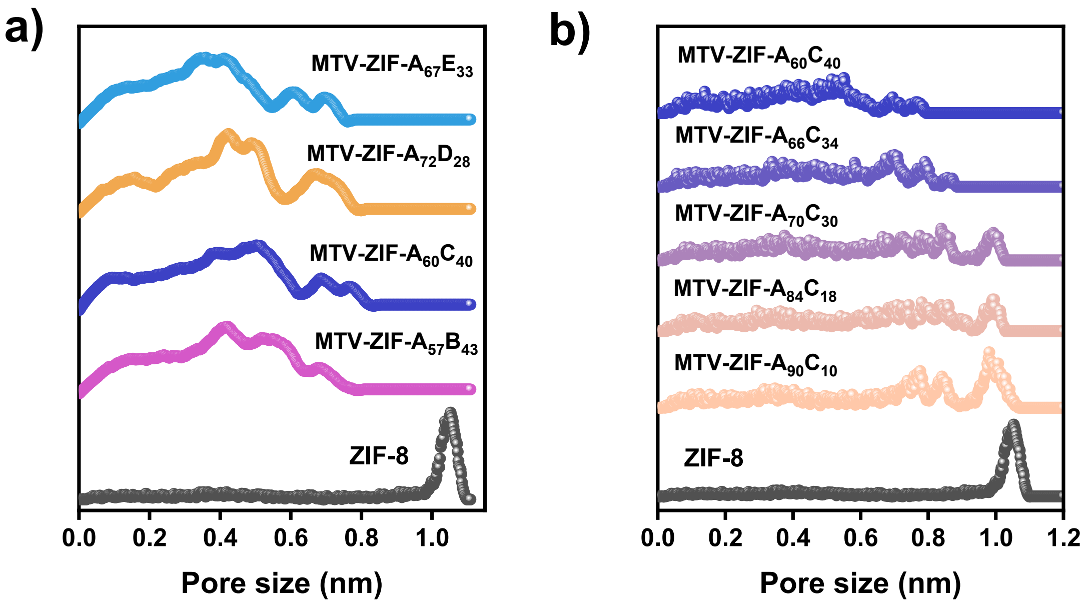

Figure S32. a) The simulated pore size distribution of MTV-ZIF-$A_{57}C_{43}$, MTV-ZIF-$A_{60}C_{40}$ MTV-ZIF-$A_{72}C_{28}$ and MTV-ZIF-$A_{67}C_{33}$; b) The simulated pore size distributions for ZIF-8 and MTV-ZIF-$A_{(100-x)}C_{x}$.

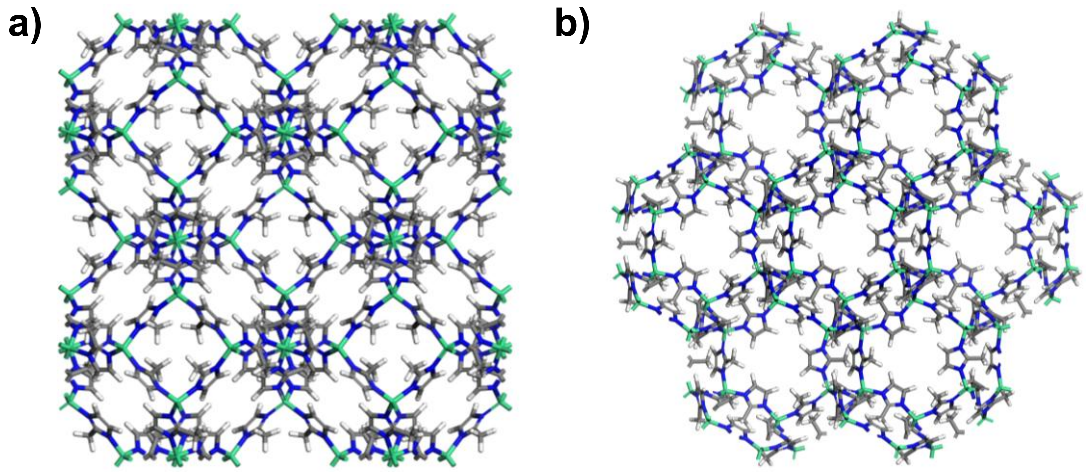

Figure S33. Vertical views of the ZIF-8 structure along a) (100) plane and b) (111) plane. N atoms are shown in dark blue, Zn atoms in bright green.

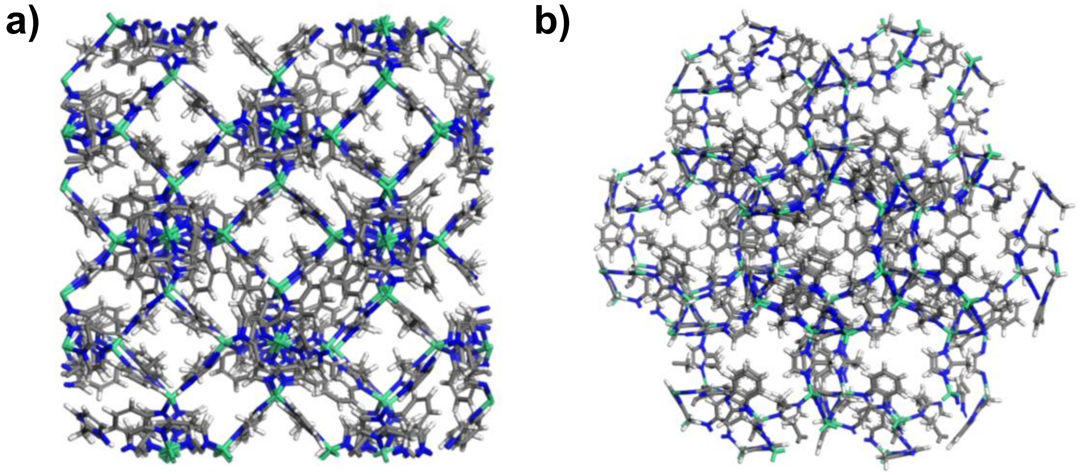

Figure S34. Vertical views of the simulated MTV-ZIF-$A_{57}B_{43}$ structure along a) (100) plane and b) (111) plane. N atoms are shown in dark blue, Zn atoms in bright green.

a) b)

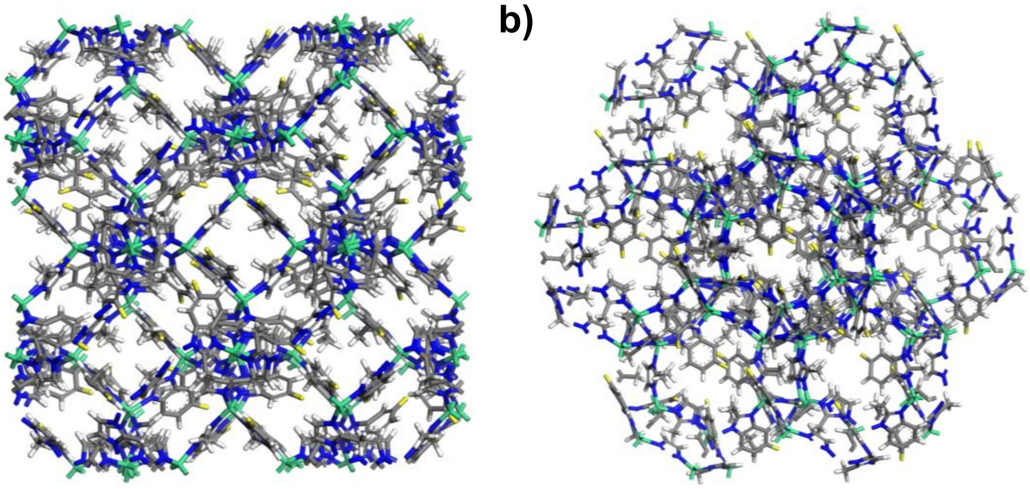

Figure S35. Vertical views of the simulated MTV-ZIF-$A_{60}C_{40}$ structure along a) (100) plane and b) (111) plane. N atoms are shown in dark blue, Zn atoms in bright green and F atoms in yellow.

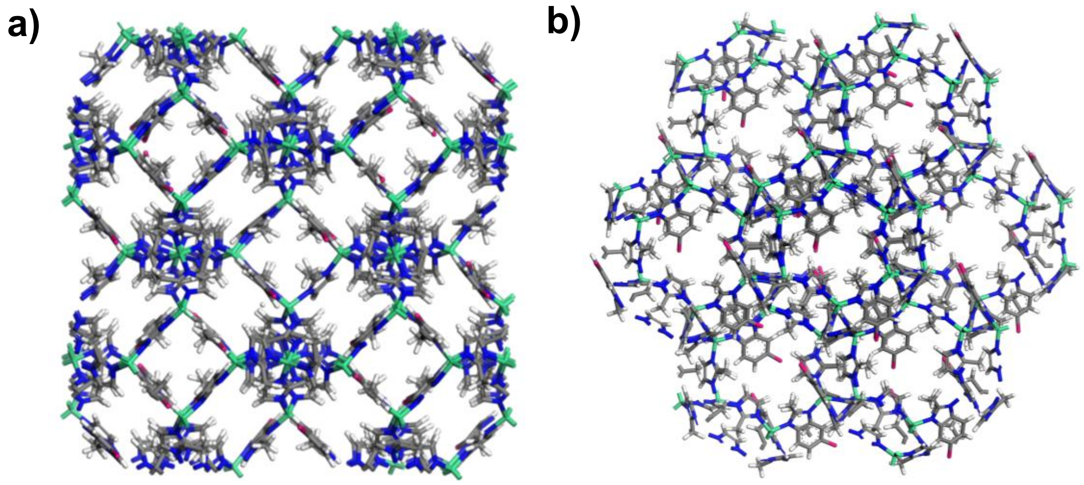

Figure S36. Vertical views of the simulated MTV-ZIF-$A_{72}D_{28}$ structure along a) (100) plane and b) (111) plane. N atoms are shown in dark blue, Zn atoms in bright green and Cl atoms in pink.

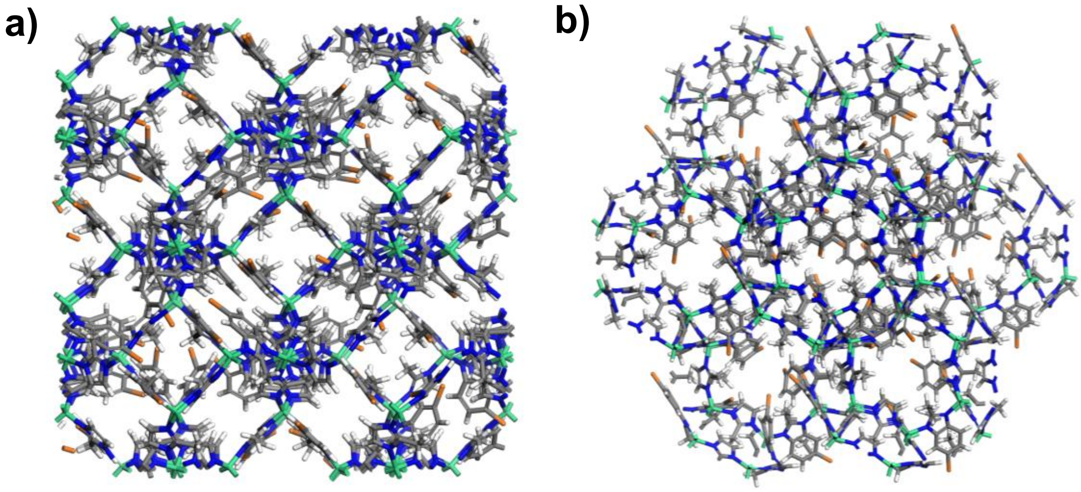

Figure S37. Vertical views of the simulated MTV-ZIF-$A_{67}E_{33}$ structure along a) (100) plane and b) (111) plane. N atoms are shown in dark blue, Zn atoms in bright green and Br atoms in orange.

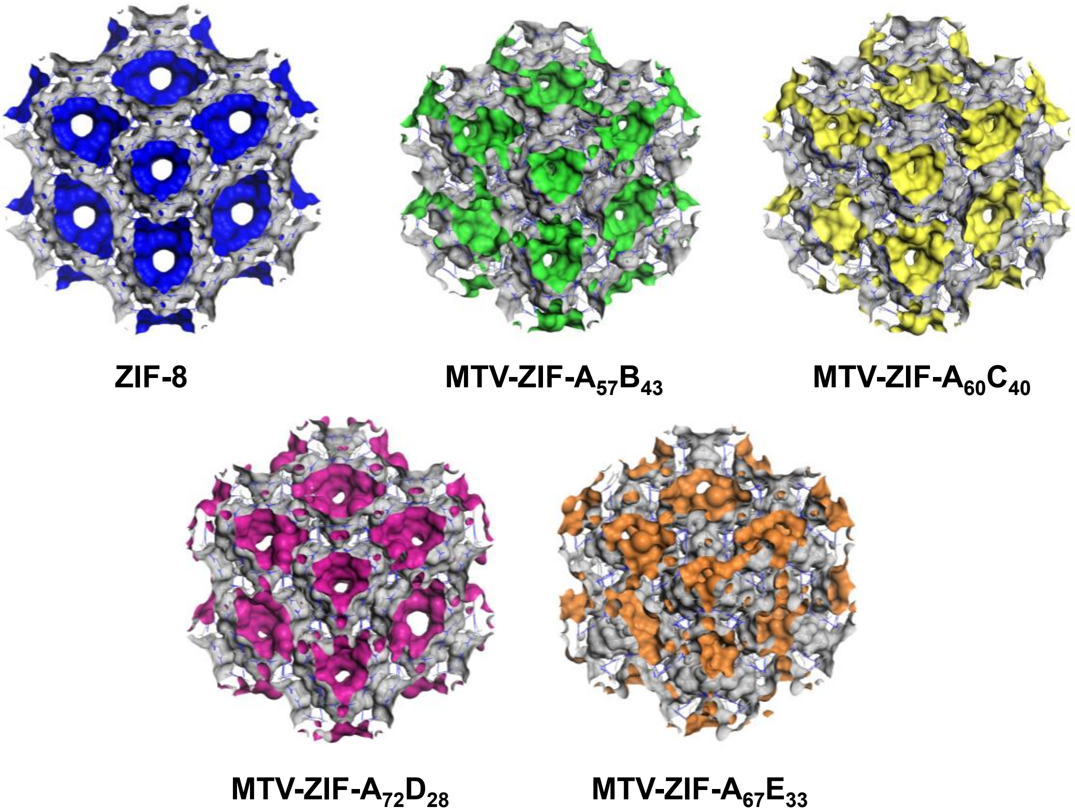

Figure S38. Comparison of simulated gas accessible channels of ZIF-8, MTV-ZIF-$A_{57}B_{43}$, MTV-ZIF-$A_{60}C_{40}$, MTV-ZIF-$A_{72}D_{28}$, and MTV-ZIF-$A_{67}E_{33}$.

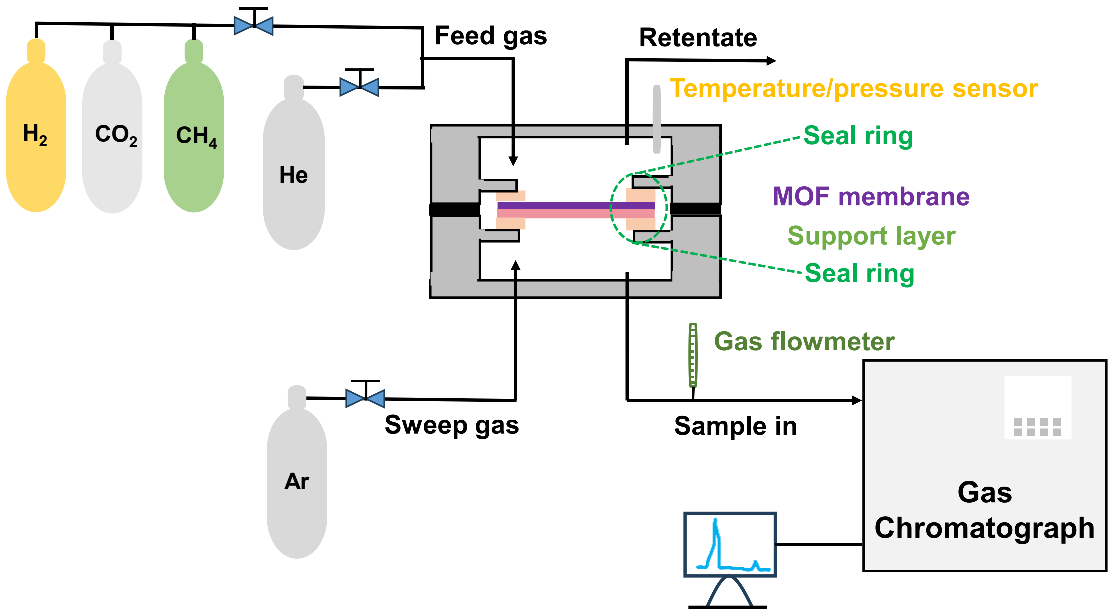

Figure S39. Schematic diagram of the gas permeation setup based on Wicke-Kallenbach method for single-gas and binary-gas tests.

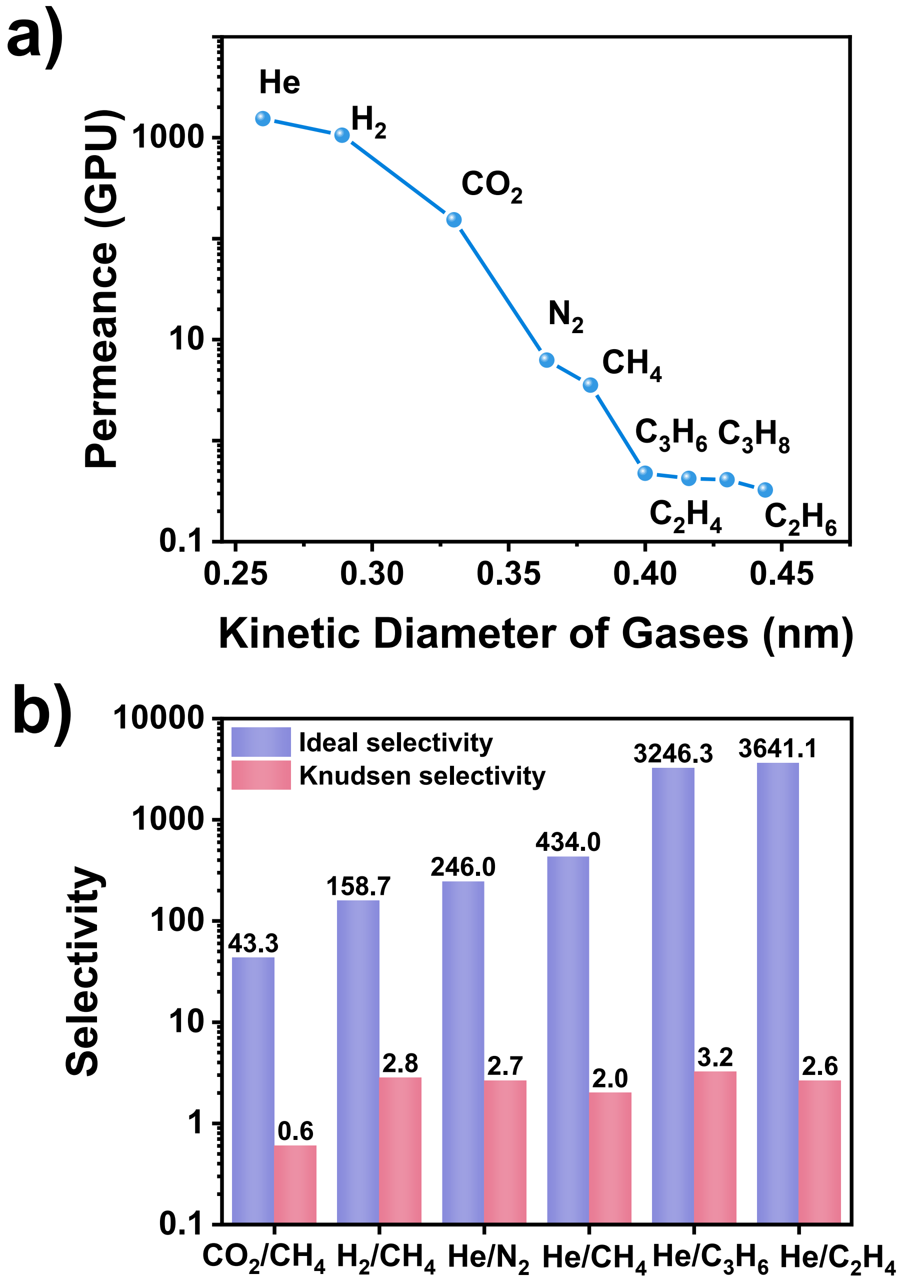

Figure S40. a) Single-gas permeance of the MTV-ZIF-$A_{60}C_{40}$ membrane as a function of gas kinetic diameter; b) Ideal selectivity and Knudsen selectivity of the MTV-ZIF-$A_{60}C_{40}$ membrane for various gas pairs.

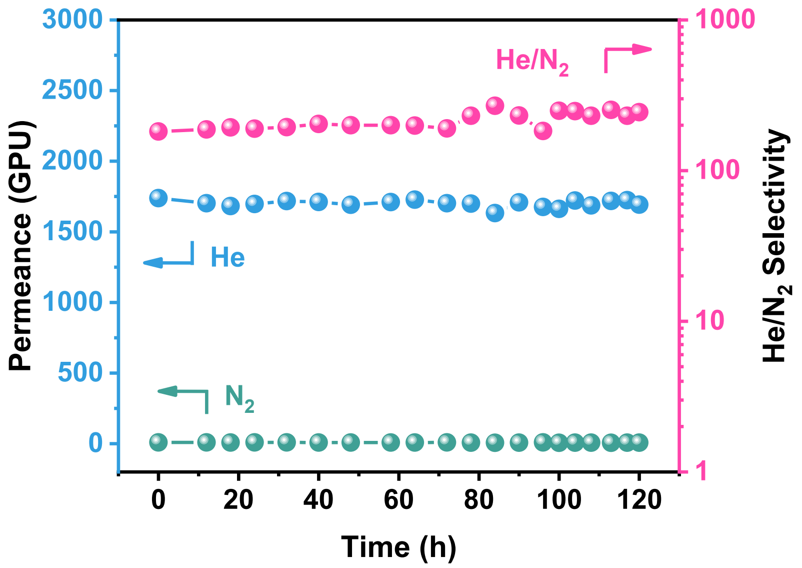

Figure S41. He/N_2 mixed-gas separation performance and extended-term stability test of the MTV-ZIF-$A_{60}C_{40}$ membranes under the 50% He/50% N_2 feed.

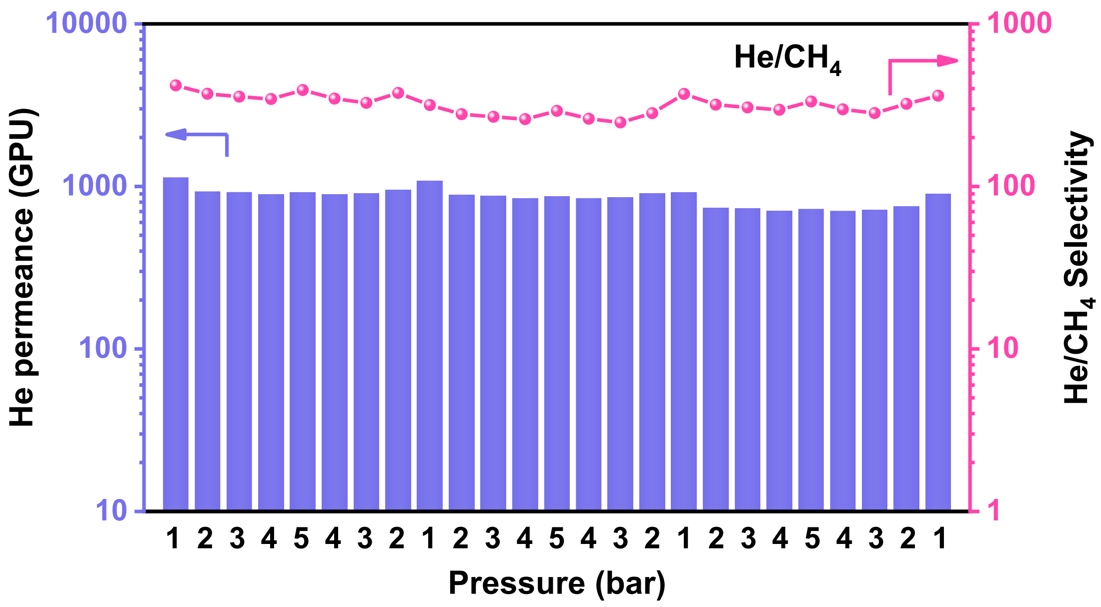

Figure S42. Pressure-cycling tests of the He/CH_4 separation performance for the MTV-ZIF-$A_{60}C_{40}$ membrane under 50% He/50% CH_4 feed.

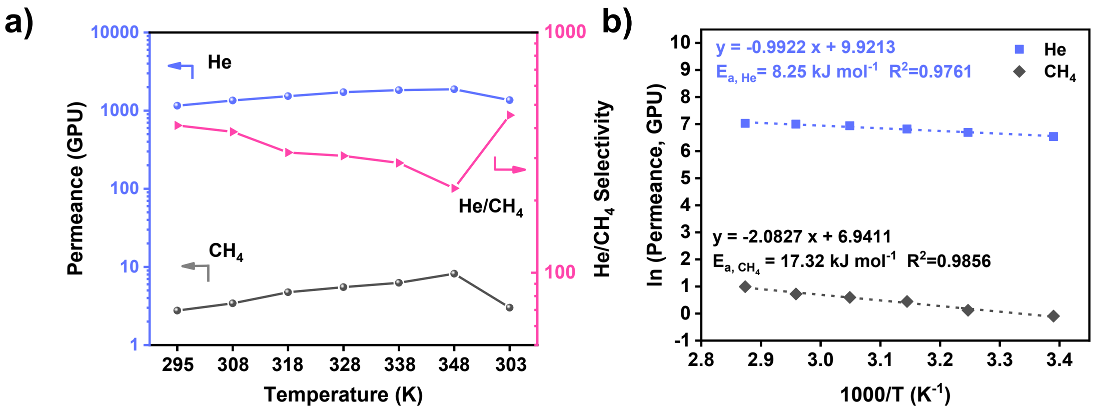

Figure S43. He/CH_4 equimolar mixed-gas separation behavior of MTV-ZIF-$A_{60}C_{40}$ membrane with varied temperature. a) Effect of temperature on the He/CH_4 separation performance of the MTV-ZIF-$A_{60}C_{40}$ membrane; b) Arrhenius plots of He and CH_4 permeances for the MTV-ZIF-$A_{60}C_{40}$ membrane.

Note: The temperature dependence of gas permeation can be stated by Arrhenius equation:

$$P_i = A_i exp\left(-\frac{E_{act,i}}{RT}\right)$$

$$\ln P_i = \ln(A_i) - \frac{E_{act,i}}{R} \cdot \frac{1}{T}$$

where P_i is the gas permeance of component i, A_i represents for the pre-exponential factor of component i, $E_{act,\ i}$ is the apparent activation energy of component i, R is the ideal gas constant (8.314 J mol^{-1} K^{-1}) and T is the absolute temperature (K). A plot of lnP_i versus $1/T$ gives a straight line, whose slope is used to calculate $E_{act,\ i}$. The $E_{act,\ He}$ and $E_{act,\ CH4}$ are 8.25 kJ mol^{-1} and 17.32 kJ mol^{-1}, respectively.

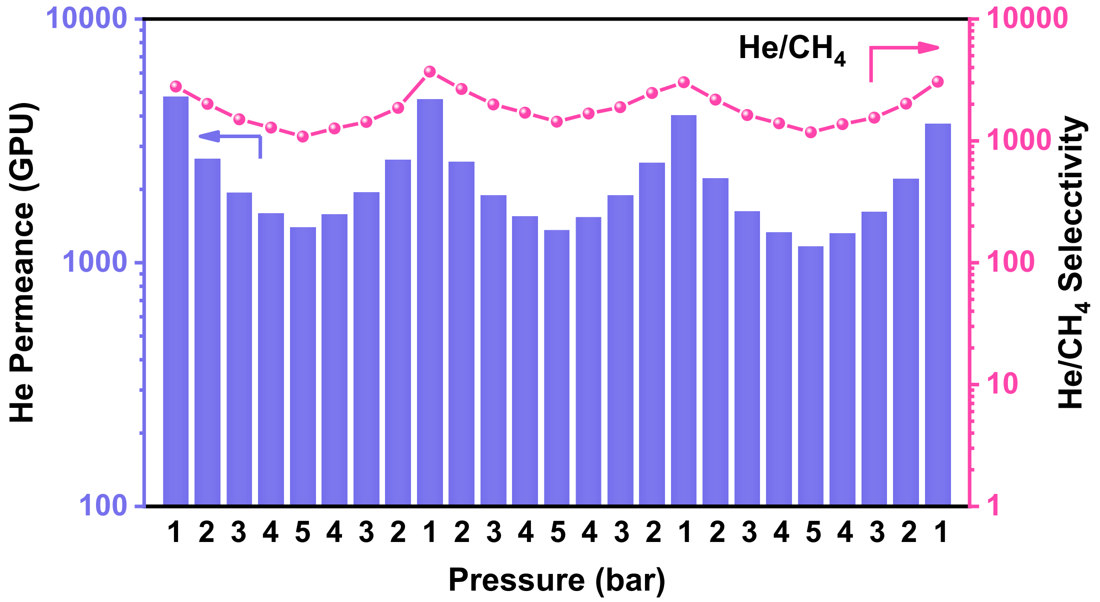

Figure S44. Pressure-cycling tests of the He/CH_4 separation performance for the MTV-ZIF-$A_{60}C_{40}$ membrane under 0.6% He/99.4% CH_4 feed.

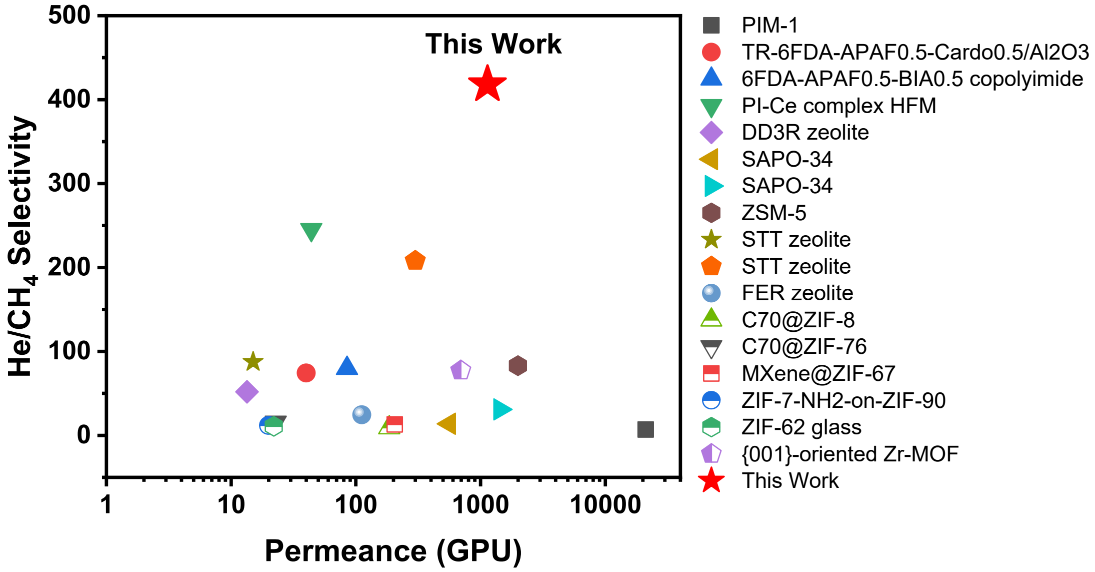

Figure S45. Comparison of the He/CH_4 separation performance for the MTV-ZIF-$A_{60}C_{40}$ membranes with other reported membranes. Note that all data were conducted using a 50:50 (v/v) He/CH_4 binary mixture at 1 bar. Comparative data are summarized in Table S9.

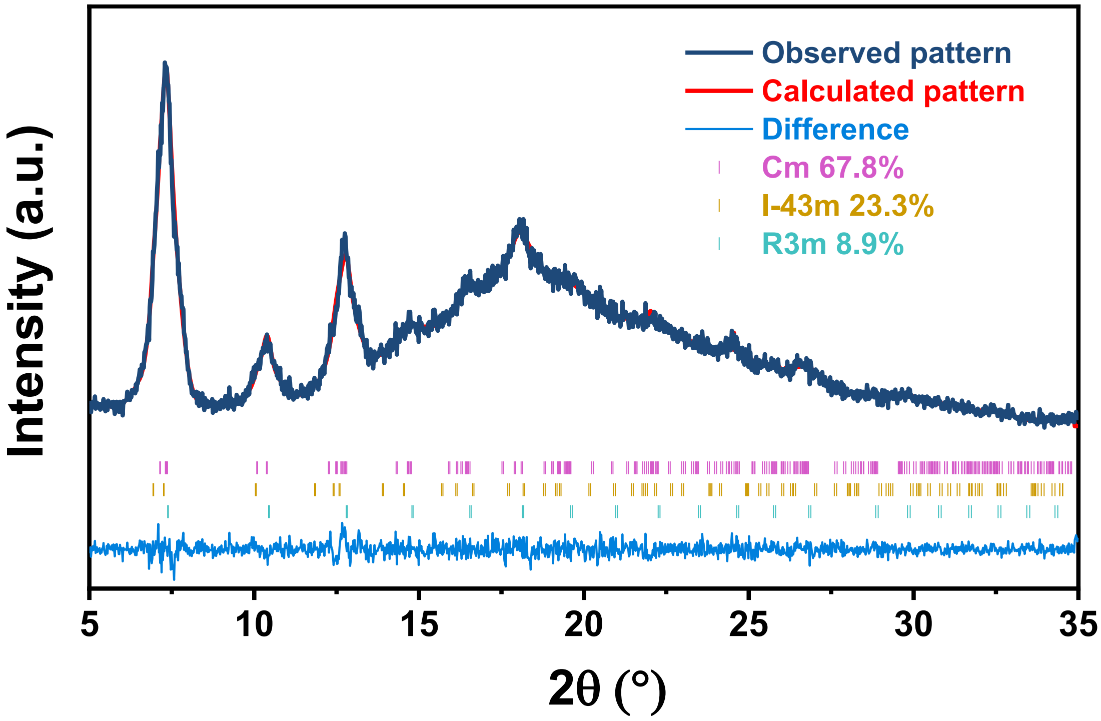

Figure S46. Rietveld refinement of the PXRD results for the ZIF-8 membrane.

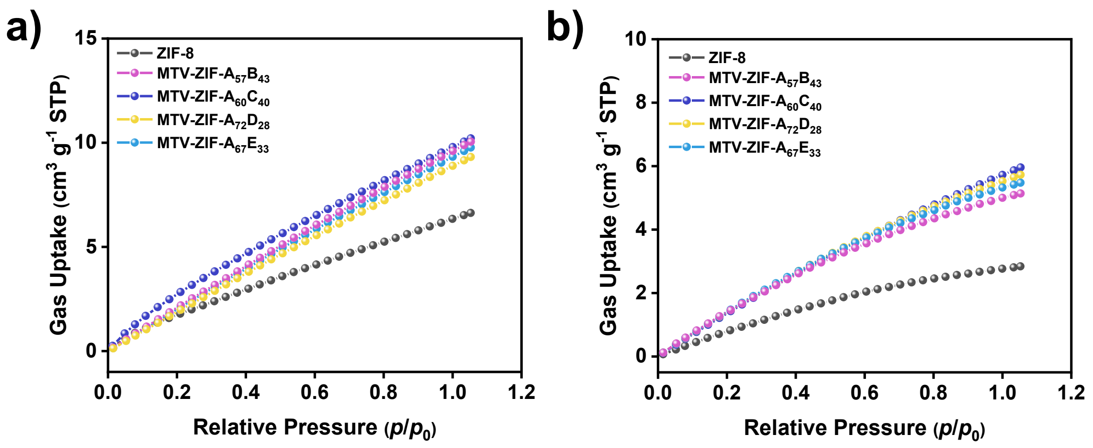

Figure S47. CH_4 adsorption isotherms of MTV-ZIFs at 298 K (a) and 313K (b).

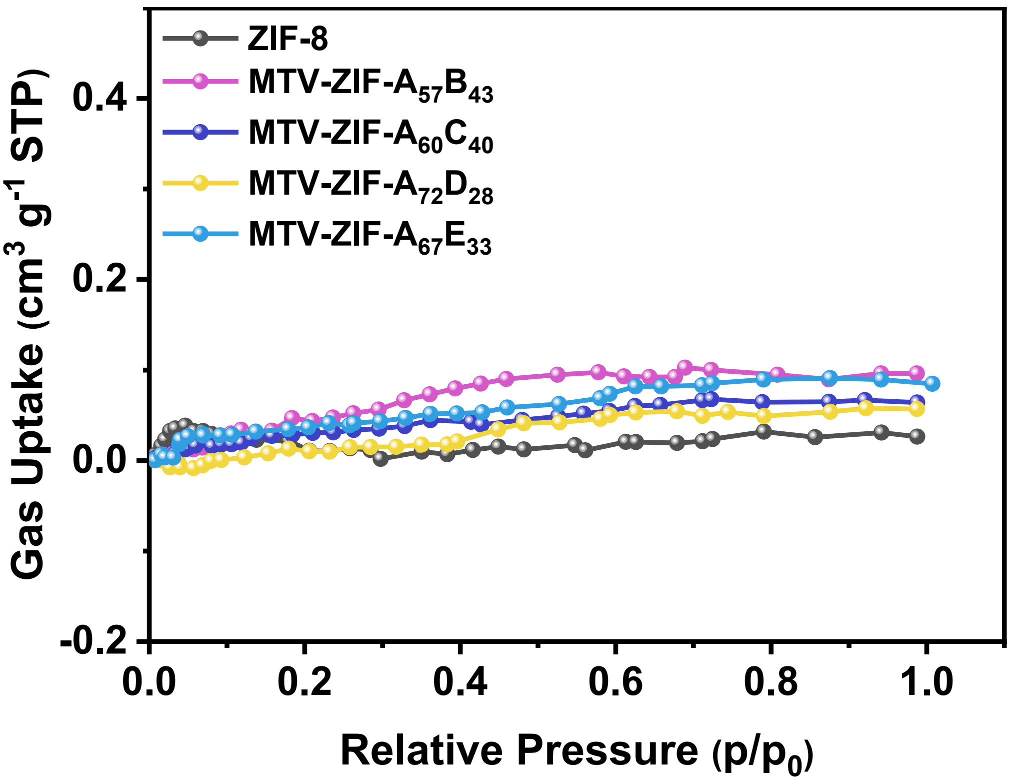

Figure S48. He adsorption isotherms of MTV-ZIFs at 273 K.

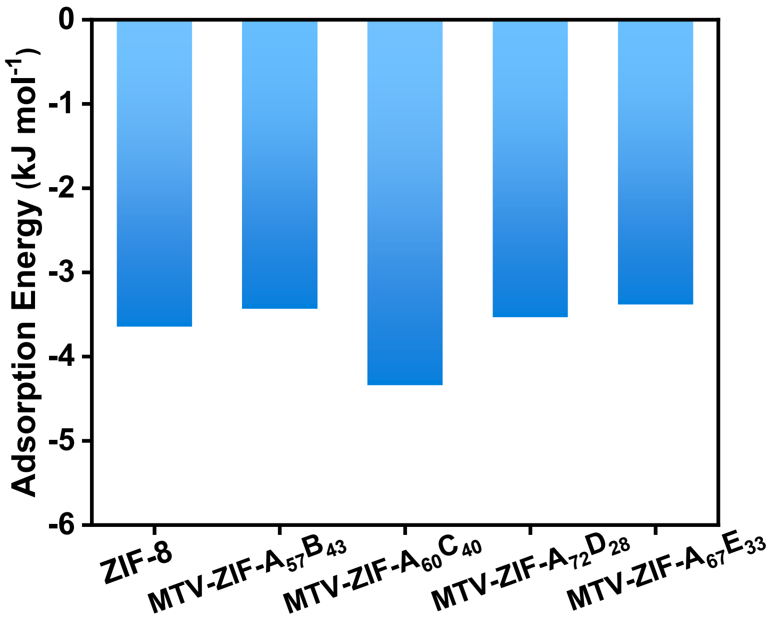

Figure S49. Comparison of calculated adsorption energy of MTV-ZIFs.

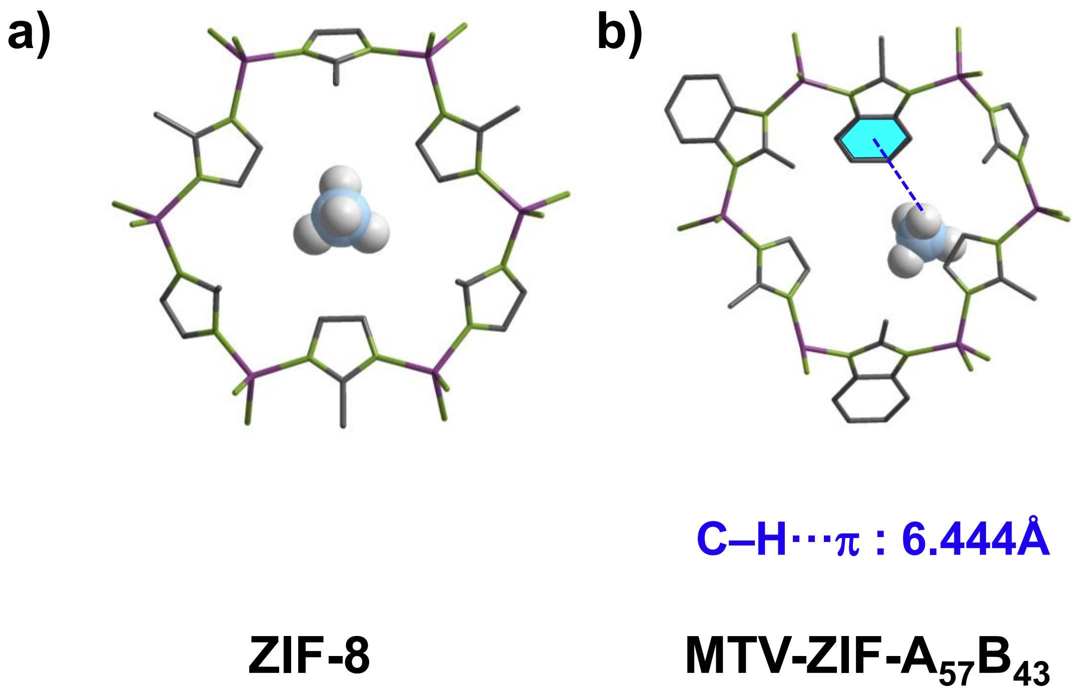

Figure S50. CH_4 adsorption sites in ZIF-8 and MTV-ZIF-$A_{57}B_{43}$ identified by GCMC simulation. The closest contacts between the framework atoms and the respective gas molecules are characterized by the distances, measured in Å.

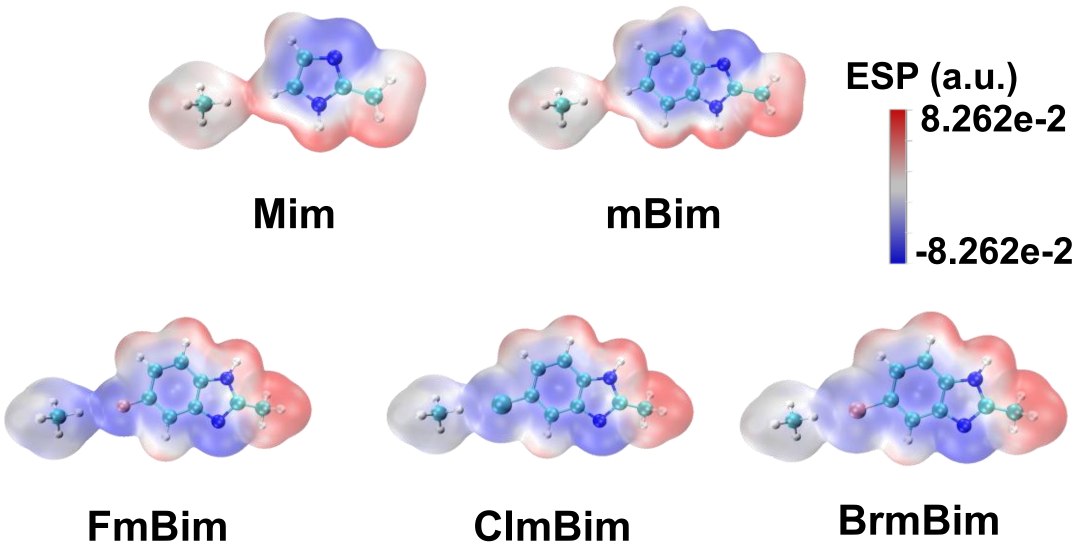

Figure S51. The color-filled electrostatic potential between CH_4 and different linkers.

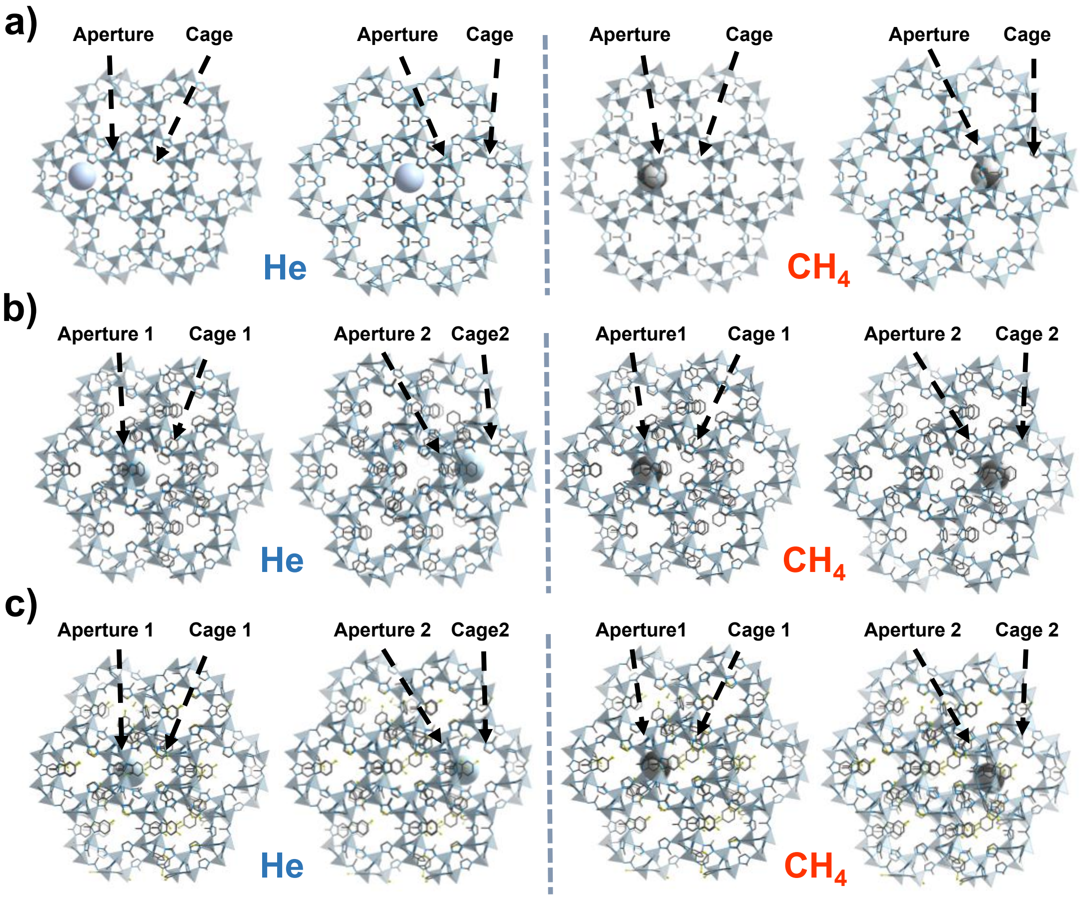

Figure S52. Snapshots of modeling corresponding to the diffusion pathways for both He and CH_4 through ZIF-8 (a), MTV-ZIF-$A_{57}B_{43}$ (b) and MTV-ZIF-$A_{60}C_{40}$ (c).

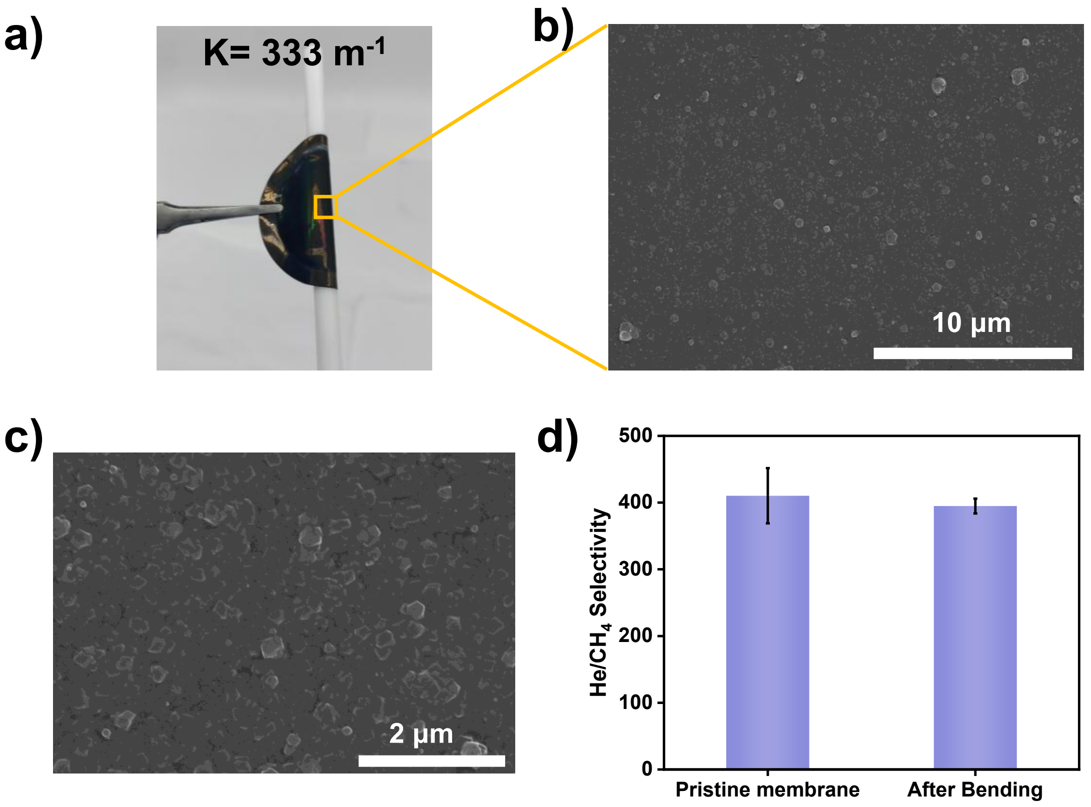

Figure S53. Membrane separation stability. a) Bending photograph of MTV-ZIF-$A_{60}C_{40}$ membrane. K is the curvature of bending; low magnification (b) and high magnification (c) top-view SEM images after 10-cycles of bending; d) He/CH_4 selectivities of MTV-ZIF-$A_{60}C_{40}$ membrane before and after bending.

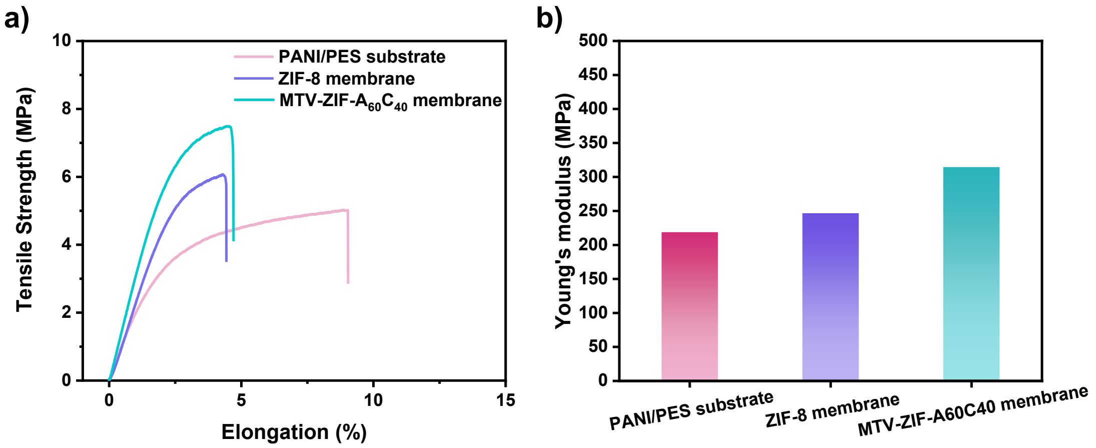

Figure S54. a) Stress-strain curves and (b) Young's modulus of PANI/PES substrate, ZIF-8 membrane and MTV-ZIF-$A_{60}C_{40}$ membrane.

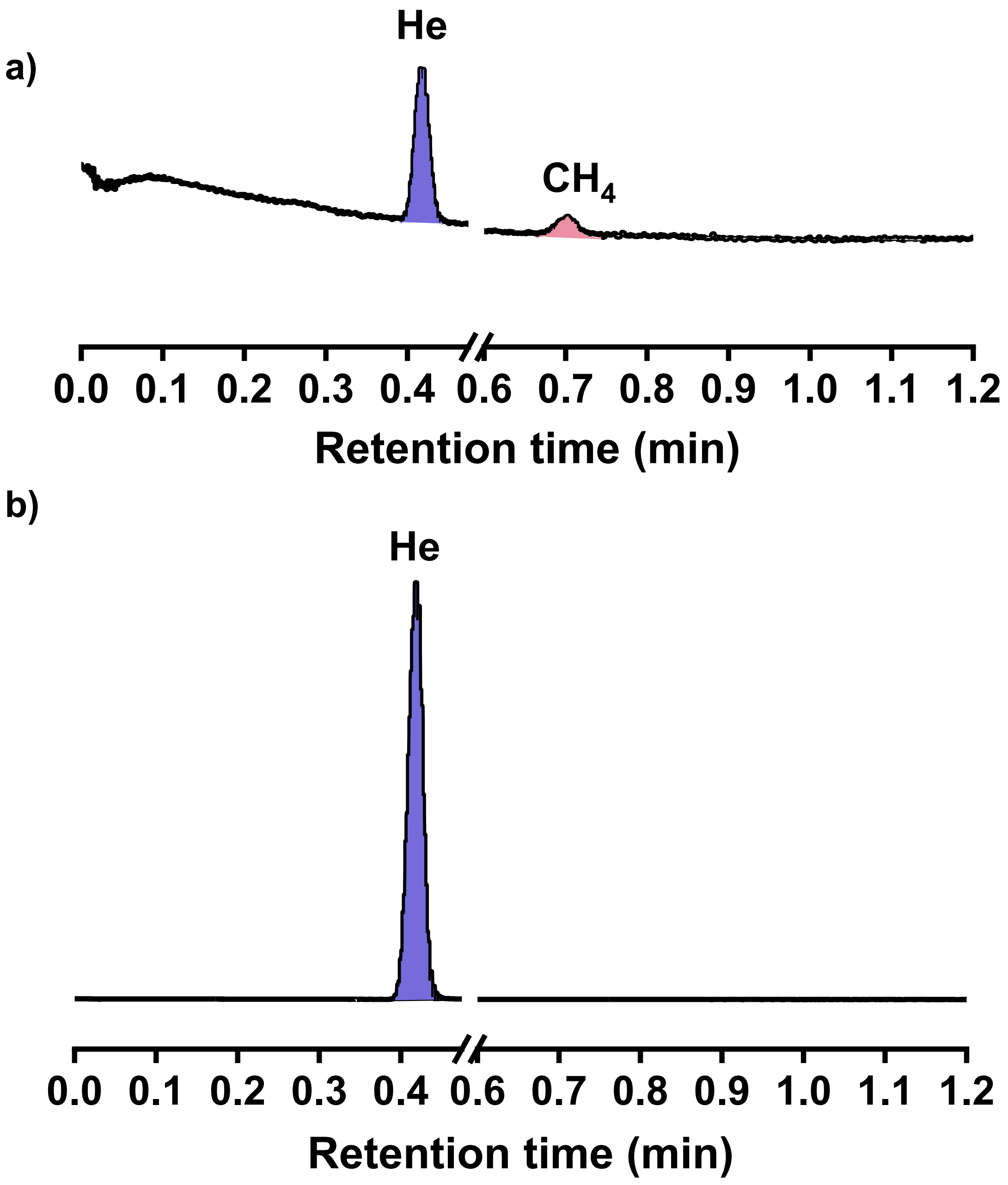

Figure S55. a) Gas chromatography (GC) chromatogram of low helium concentration (0.6% He/99.4% CH_4) feed gas permeating through the MTV-ZIF-$A_{60}C_{40}$ membrane in Stage 1; b) GC chromatogram of the permeate 1 gas permeating through the MTV-ZIF-$A_{60}C_{40}$ membrane in Stage 2.

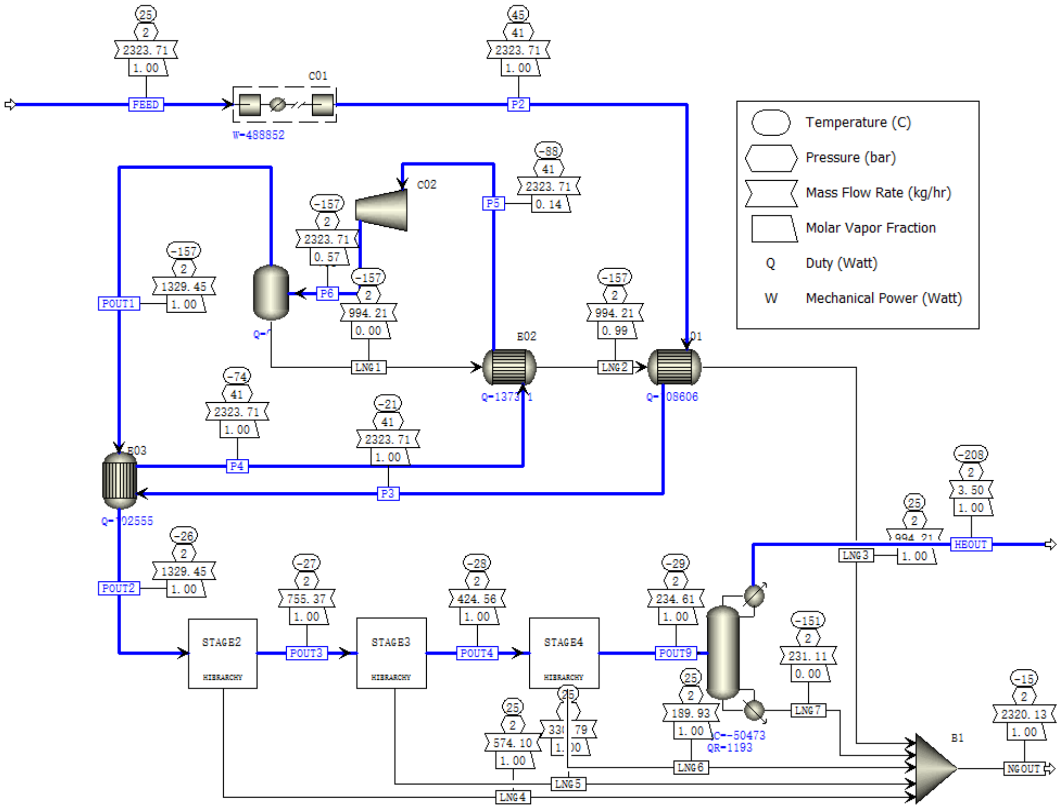

Figure S56. Process flow diagram of the conventional cryogenic distillation process for extracting He from the 0.6% He/99.4% CH_4 feed.

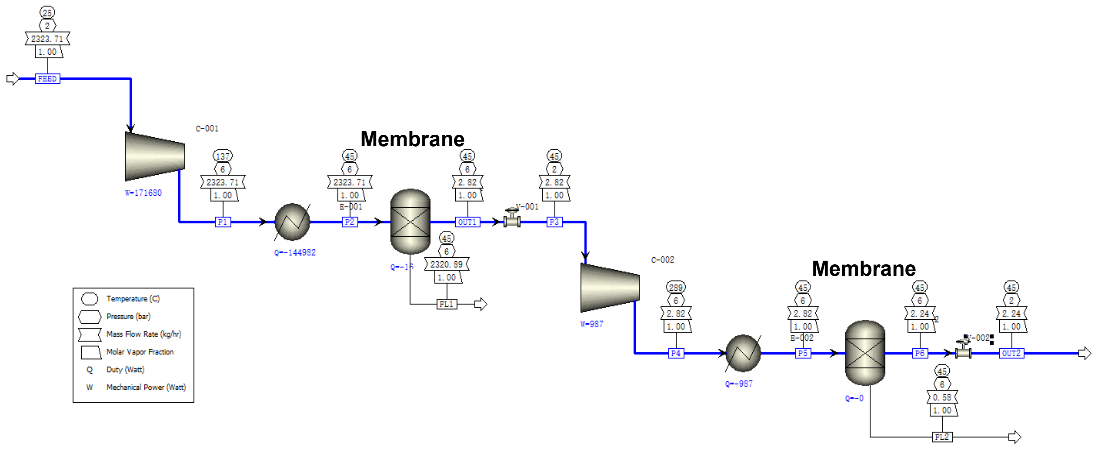

	Units	FEED	FL1	FL2	OUT1	OUT2	P1	P2	P3	P4	P5	P6
Description												
MIXED Substream												
Phase		Vapor Phase	Vapor Phase	Vapor Phase	Vapor Phase	Vapor Phase	Vapor Phase	Vapor Phase	Vapor Phase	Vapor Phase	Vapor Phase	Vapor Phase
Temperature	C	25	45	45	45	45.194703	137.010946	45	45.193113	288.706303	45	45
Pressure	MPag	0.1	0.488675	0.488675	0.488675	0.098675	0.498675	0.488675	0.098675	0.498675	0.488675	0.488675
Average MW		15.970519	16.028242	4.11131158	4.02452612	4.00263213	15.970519	15.970519	4.02452612	4.02452612	4.02452612	4.00263213
Mass Density	kg/cum	1.30276733	3.61246024	0.91453897	0.89521815	0.30216987	2.82041229	3.59903171	0.30382468	0.51604501	0.89521815	0.89034421
Molar Density	kmol/cum	0.08157326	0.22538094	0.22244458	0.22244064	0.07549279	0.17660117	0.22535471	0.07549328	0.12822504	0.22244064	0.22243968
Mass Vapor Fraction		1	1	1	1	1	1	1	1	1	1	1
Molar Vapor Fraction		1	1	1	1	1	1	1	1	1	1	1
Mass Flows	kg/hr	2323.71052	2320.89466	0.57950036	2.81585695	2.23635659	2323.71052	2323.71052	2.81585695	2.81585695	2.81585695	2.23635659
Mass Fractions												
Mole Flows	kmol/hr	145.5	144.800326	0.14095267	0.69967416	0.55872149	145.5	145.5	0.69967416	0.69967416	0.69967416	0.55872149
HE	kmol/hr	0.873	0.1746	0.13968	0.6984	0.55872	0.873	0.873	0.6984	0.6984	0.6984	0.55872
CH4	kmol/hr	144.627	144.625726	0.00127267	0.00127416	trace	144.627	144.627	0.00127416	0.00127416	0.00127416	trace
Mole Fractions												
HE		0.006	0.0012058	0.99097092	0.99817892	0.99999733	0.006	0.006	0.99817892	0.99817892	0.99817892	0.99999733
CH4		0.994	0.9987942	0.00902908	0.00182108	2.6682 PPM	0.994	0.994	0.00182108	0.00182108	0.00182108	2.6682 PPM

Figure S57. Process flow diagram of the two-stage cascade membrane process for extracting He from the 0.6% He/99.4% CH_4 feed and related table of material balance.

Note: Industrial helium recovery is typically closely linked to LNG production and/or nitrogen rejection (NRU) units. Before entering the cryogenic section of an LNG plant, the feed gas is generally pretreated to remove acid gases (e.g., CO_2 and H_2S), water, mercury, and heavy hydrocarbons[18-19]. The gas then undergoes NGL recovery/fractionation and/or an NRU prior to liquefaction/storage; in practice, the conventional cryogenic distillation process used for helium-related separations is closely aligned with the NRU-type cryogenic operation (Figure S56). Our membrane technology specifically targets methane-rich streams containing trace helium, which represents one of the most challenging yet most impactful scenarios for reducing downstream load. In this context, a two-stage cascaded membrane process can be considered as a membrane-based alternative to cryogenic separation, enabling helium recovery from natural gas through an entirely membrane-driven separation scheme.

3. Supporting tables

Table S1. Reactant ratios for MTV-ZIF-AB membranes.

Membranes	Reactants		
	$Zn(NO_3)_2 \cdot 6H_2O$ (mmol)	Mim (linker A, mmol)	Mbim (linker B, mmol)
ZIF-8/MTV-ZIF-$A_{100}B_{0}$	1.3	5.2	0
MTV-ZIF-$A_{90}B_{10}$	1.3	4.94	0.26
MTV-ZIF-$A_{82}B_{18}$	1.3	4.68	0.52
MTV-ZIF-$A_{71}B_{29}$	1.3	4.16	1.04
MTV-ZIF-$A_{59}B_{41}$	1.3	3.64	1.56
MTV-ZIF-$A_{57}B_{43}$	1.3	3.12	2.08

Table S2. Reactant ratios for MTV-ZIF-AC membranes.

Membranes	Reactants		
	$Zn(NO_3)_2 \cdot 6H_2O$ (mmol)	Mim (linker A, mmol)	Fmbim (linker C, mmol)
ZIF-8/MTV-ZIF-$A_{100}C_{0}$	1.3	5.2	0
MTV-ZIF-$A_{90}C_{10}$	1.3	4.94	0.26
MTV-ZIF-$A_{82}C_{18}$	1.3	4.68	0.52
MTV-ZIF-$A_{70}C_{30}$	1.3	4.16	1.04
MTV-ZIF-$A_{66}C_{34}$	1.3	3.90	1.30
MTV-ZIF-$A_{60}C_{40}$	1.3	3.38	1.82
MTV-ZIF-$A_{55}C_{45}$	1.3	3.12	2.08

Table S3. Reactant ratios for MTV-ZIF-AD membranes.

Membranes	Reactants		
	$Zn(NO_3)_2·6H_2O$ (mmol)	Mim (linker A, mmol)	Clmbim (linker D, mmol)
ZIF-8/MTV-ZIF-$A_{100}D_0$	1.3	5.2	0
MTV-ZIF-$A_{92}D_8$	1.3	4.94	0.26
MTV-ZIF-$A_{85}D_{15}$	1.3	4.68	0.52
MTV-ZIF-$A_{77}D_{23}$	1.3	4.16	1.04
MTV-ZIF-$A_{72}D_{28}$	1.3	3.90	1.30
MTV-ZIF-$A_{65}D_{35}$	1.3	3.64	1.56

Table S4. Reactant ratios for MTV-ZIF-AE membranes.

Membranes	Reactants		
	$Zn(NO_3)_2·6H_2O$ (mmol)	Mim (linker A, mmol)	Brmbim (linker E, mmol)
ZIF-8/MTV-ZIF-$A_{100}E_0$	1.3	5.2	0
MTV-ZIF-$A_{90}E_{10}$	1.3	4.94	0.26
MTV-ZIF-$A_{86}E_{14}$	1.3	4.68	0.52
MTV-ZIF-$A_{76}E_{24}$	1.3	4.16	1.04
MTV-ZIF-$A_{67}E_{33}$	1.3	3.90	1.30

Table S5. He/CH_4 mixed-gas separation performance of the MTV-ZIF-$A_{(100-x)}B_x$ membranes. At least three independent membranes for each percentage are prepared and tested.

Membrane	Number	He permeance (GPU)	CH_4 permeance (GPU)	Selectivity
ZIF-8/MTV-ZIF-$A_{100}B_0$	M1	2186	210	10.40
	M2	1703	118	14.46
	M3	1884	126	14.89
	Average	1925	151	13.25
	Standard deviation	199.25	41.62	2.021
MTV-ZIF-$A_{90}B_{10}$	M4	1501	137	10.96
	M5	1503	144	10.42
	M6	2121	115	18.39
	M7	2174	116	18.76
	Average	1825	128	14.63
	Standard deviation	323.60	12.72	3.938
MTV-ZIF-$A_{82}B_{18}$	M8	1436	102	14.11
	M9	1524	78	19.57
	M10	1660	88	18.78
	Average	1540	89	17.49
	Standard deviation	91.99	9.762	2.408
MTV-ZIF-$A_{71}B_{29}$	M11	854	33	26.20
	M12	1075	41	25.95
	M13	824	16	51.68
	Average	918	30	34.61
	Standard deviation	112.04	10.57	12.07

MTV-ZIF-$A_{59}B_{41}$	M14	673	4.597	146.43
	M15	324	3.065	105.77
	M16	713	3.960	180.15
	Average	570	3.874	144.12
	Standard deviation	174.73	0.6279	30.41
MTV-ZIF-$A_{57}B_{43}$	M17	210	0.6298	333.36
	M18	245	1.542	158.97
	M19	205	1.393	147.53
	M20	228	1.219	187.20
	Average	222	1.196	206.77
	Standard deviation	15.73	0.346	74.50

Table S6. He/CH_4 mixed-gas separation performance of the MTV-ZIF-$A_{(100-x)}C_x$ membranes. At least three independent membranes for each percentage are prepared and tested.

Membrane	Number	He permeance (GPU)	CH_4 permeance (GPU)	Selectivity
MTV-ZIF-$A_{90}C_{10}$	M1	1300	31.18	41.69
	M2	997	20.97	47.53
	M3	1033	31.62	32.67
	Average	1110	27.92	40.63
	Standard deviation	135.07	4.921	6.11
MTV-ZIF-$A_{82}C_{18}$	M4	661	6.892	95.92
	M5	846	26.04	32.48
	M6	906	16.228	55.82
	Average	804	16.386	61.41
	Standard deviation	104.14	7.818	26.20
MTV-ZIF-$A_{70}C_{30}$	M7	811	16.722	48.53
	M8	1032	6.327	163.11
	M9	1107	11.564	95.74
	Average	983	11.538	102.46
	Standard deviation	125.47	4.244	47.02
MTV-ZIF-$A_{66}C_{34}$	M10	546	2.849	191.79
	M11	636	3.466	183.46
	M12	430	2.075	207.34
	M13	503	2.456	204.70

	M14	505	3.271	154.48
	Average	524	2.824	188.35
	Standard deviation	67.28	0.5117	19.03
MTV-ZIF-$A_{60}C_{40}$	M15	1781	3.851	462.35
	M16	789	1.937	407.12
	M17	902	2.497	361.18
	M18	1080	2.443	442.05
	Average	1137	2.682	418.18
	Standard deviation	385.42	0.7094	38.38
MTV-ZIF-$A_{55}C_{45}$	M19	795	2.664	298.46
	M20	284	0.9430	301.34
	M21	337	1.088	309.76
	Average	472	1.565	303.18
	Standard deviation	229.38	0.7792	4.80

Table S7. He/CH_4 mixed-gas separation performance of the MTV-ZIF-$A_{(100-x)}D_x$ membranes. At least three independent membranes for each percentage are prepared and tested.

Membrane	Number	He permeance (GPU)	CH_4 permeance (GPU)	Selectivity
MTV-ZIF-$A_{92}D_8$	M1	2132	245	8.70
	M2	2603	300	8.67
	M3	2815	312	9.03
	Average	2517	286	8.799
	Standard deviation	285.29	29.17	0.1615
MTV-ZIF-$A_{85}D_{15}$	M4	2351	193	12.16
	M5	2055	89	23.12
	M6	1929	100	19.36
	Average	2112	127	18.21
	Standard deviation	176.89	46.93	4.548
MTV-ZIF-$A_{77}D_{23}$	M7	1771	97	18.20
	M8	1309	59	22.24
	M9	1237	51	24.11
	M10	1611	102	15.83
	Average	1482	77	20.10
	Standard deviation	218.00	22.44	3.261
MTV-ZIF-$A_{72}D_{28}$	M11	819	11	74.85
	M12	724	12	60.74
	M13	1007	11	88.13
	M14	1417	16	90.47

	Average	991.5	12	78.55
	Standard deviation	265.94	1.867	11.88
MTV-ZIF-$A_{65}D_{35}$	M15	1214	19	63.32
	M16	993	13	75.24
	M17	958	17	54.79
	Average	1055	16	64.45
	Standard deviation	113.34	2.513	8.385

Table S8. He/CH_4 mixed-gas separation performance of the MTV-ZIF-$A_{(100-x)}E_x$ membranes. At least three independent membranes for each percentage are prepared and tested.

Membrane	Number	He permeance (GPU)	CH_4 permeance (GPU)	Selectivity
MTV-ZIF-$A_{90}E_{10}$	M1	1261	82.9	15.21
	M2	1856	112.5	16.49
	M3	1751	80.1	21.86
	Average	1623	91.8	17.85
	Standard deviation	259.05	14.668	2.88
MTV-ZIF-$A_{86}E_{14}$	M4	1225	49.88	24.56
	M5	1607	57.34	28.02
	M6	1050	26.7	39.33
	Average	1294	44.64	30.64
	Standard deviation	232.44	13.045	6.309
MTV-ZIF-$A_{76}E_{24}$	M7	1277	26.51	48.17
	M8	1372	20.53	66.84
	M9	740	5.88	125.8
	M10	854	10.41	82.02
	Average	1061	15.83	80.71
	Standard deviation	268.89	8.130	28.67
MTV-ZIF-$A_{67}E_{33}$	M11	913	12.77	71.51
	M12	668	5.53	120.96
	M13	836	10.89	76.81
	M14	492	5.04	97.67
	Average	727	8.55	91.74
	Standard deviation	162.29	3.345	19.50

Table S9. Comparative overview of He/CH_4 separation performance of various types of membranes.

Membranes		He permeance (GPU)	He/CH_4 Selectivity	Note[a]	Ref.
Polymer	80 nm PIM-1	21087	7		20
	TB-DM	0.86-1.3	238	0.3% He/99.7% CH_4 at 4bar	21
	ITB-DM	0.62-0.92	268	0.3% He/99.7% CH_4 at 4bar	21
	TB-OT	2-3	88	0.3% He/99.7% CH_4 at 4bar	21
	ITB-OT	1.5-2.25	194	0.3% He/99.7% CH_4 at 4bar	21
	TB-Trip	68-101	5.5	0.3% He/99.7% CH_4 at 4bar	21
	ITB-Trip	61-91	17.2	0.3% He/99.7% CH_4 at 4bar	21
	PI-0.25	3.9	32	single gas	22
	IPI-0.25	3.1	30	single gas	22
	PI-0.50	2.7	44	single gas	22
	PI-0.75	2.1	70	single gas	20
	IPI-0.50	1.5	76	single gas	22
	FPIM-5	16.6	3770	single gas	23
	TR-6FDA-APAF0.5-Cardo0.5/Al_2O_3	40	74.3		24
	TR-6FDA-APAF0.5-Cardo0.5/Al_2O_3	45.6	83	0.2% He/99.8% CH_4	24
	Poly(PFMD)	7	1650	single gas	25

	Poly(PFMMD-co-PFMD)-3	2970	405	single gas	26
	Poly(PFMMD-co-CTFE)-3	804	900	single gas	26
	PBDI	46	1000	single gas	27
Hollow fiber	6FDA-APAF$_{0.5}$-BIA$_{0.5}$ copolyimide	85	80		28
	TR-X1-F1(TR-PBOI)	404	4795	single gas	29
	PIM-1-F5	202	61	single gas	29
	Ultem1000(PEI)	55	98	single gas, at 3.5 bar	30
	Cellulose acetate	106	31	single gas	31
	Aomatic polyamide	6~8	36~40	single gas	32
	Torlon®	7.4	370	single gas, at 13 bar	33
	P84	33.4	4.99	single gas	34
	P84	1.17	16.7	single gas	35
	6FDA-DAM-DABA	340	20	single gas, at 1.3 bar	36
	crosslinked 6FDA-mPDA$_{0.65}$-DABA$_{0.3}$-TFMB$_{0.05}$ copolyimide	18	210	single gas, at 5 bar	37
	PI-Ce complex HFM	44	245	at 5 bar and 308K	38
	6FDA-mPDA0.9-TFMB0.1 copolyimide	72.1	178	single gas, at 5 bar	39
	HFM-800-F-1min	32.4	2895	single gas, at 10 bar	40
	PI-TFMB-HF@400	25	269	single gas, at 2 bar and 308K	41

	PI-TFMB-HF@400	15	229	He/CO_2/CH_4 (0.3/49.7/50, v/v/v) at 4 bar	41
	PI-TFMB-HF@400	19	312	He/CO_2/CH_4 (0.3/49.7/50, v/v/v) at 40 bar	41
	HFM-50W-65Pa-500s	147	697	He/CO_2/CH_4 (0.3/5/94.7, v/v/v) at 4 bar	42
	HFM-50W-65Pa-500s	163	1005	He/CO_2/CH_4 (0.3/5/94.7, v/v/v) at 40 bar	42
CMS	Organosolve-lignin, and a phenol resin	20.03	21.01	single gas	43
	PIM-PI/alumina precursor	40.01	11.23	single gas	44
	PVDC-PVC	38.01	27.81	single gas	45
	PPO on ceramic support	716.11	326.14	single gas	46
	metal oxide/PIM−PI	316.23	11.29	single gas	47
	Kapton	283.52	37.08	single gas	48
	TFC-10%-600	1248.48	137.07	single gas	49
	CA-3-600	31.9	375	single gas, at 2 bar	50
	FCMS550-60s	26	5329	single gas	51
	FCMS550-60s	13.4	4114	1% He/99% CH_4, at 20 bar	51
Inorganic	DD3R zeolite	13.4	52	at 3 bar	52
	DD3R zeolite	8.9	44	0.22% He/99.78% CH_4, at 7 bar	52
	SAPO-34	561.5	13.8		52
	SAPO-34	1429.4	31		53
	SAPO-34	116.1	4.8	single gas	54
	SSZ-13	163.8	5	single gas	55

	ZSM-5/PTFE	1995	83		56
	STT zeolite	15	87.6		57
	STT zeolite	300	208		58
	FER zeolite	111.7	24.5		59
MMMs	Matrimid/40% MgO nanocomposite	0.92	118.1	single gas	60
	PMMA/21% UiO-66	0.0725-0.145	830	single gas	61
	PSU-CNT	49.15	1.8	single gas	62
	6FAB/20% UiO-66-NH_2	3.2-3.9	399	single gas	63
	6FAB/30% UiO-66-NH_2	4.5-5.4	296.5	single gas	63
	PSU/20% MIL-116(Ga)-formate	0.55	1190	4% He/96% CH_4 at 353K	64
MOFs	3.50% C70@ZIF-8	185.4	9.2		65
	ZIF-76	79.8	4.6		64
	3.79% C70@ZIF-76	22.8	15.7		64
	ZIF-76-mbIm	980	2		64
	3.54% C70@ZIF-76-mbIm	587	2.1		64
	Cu-BTC	4140	2.1	single gas	66
	IRMOF-3	2986	1.6	single gas	67
	IRMOF-3-AM6	2389	1.3	single gas	67
	$[Cu_2(bza)_4(pyz)]n$	7.3	8	single gas	68
	ZIF-8	570	4.6	single gas	69
	MXene@ZIF-67	205	13		70
	MXene@ZIF-67	246	17.9	at 393k	70
	ZIF-7-NH_2-on-ZIF-90	19.95	11.73		71

	ZIF-62 polycrystal	198.2	2.5	at 303k	72
	ZIF-62 glass	22	10.8	at 303k	72
	ZIF-62 glass	51.6	13.9	at 423k	72
	(001)-oriented-Zr-MOF	695.1	77.3		73
**MTV-ZIFs**	MTV-ZIF-$A_{60}C_{40}$	1137	418	50% He/50% CH_4 at 1 bar	**This work**
	MTV-ZIF-$A_{60}C_{40}$	916	391	50% He/50% CH_4 at 5 bar	
	MTV-ZIF-$A_{60}C_{40}$	4341	3174	0.6% He/99.4% CH_4 at 1 bar	
	MTV-ZIF-$A_{60}C_{40}$	1355	876	0.6% He/99.4% CH_4 at 5 bar	

a: Unless otherwise specified, all gas permeation tests were conducted using a 50:50 (v/v) He/CH_4 binary mixture at 1 bar and room temperature. Specific variations in feed composition, pressure, temperature, or single-gas/mixed-gas conditions are explicitly indicated where applicable.

4. References

(1) Wang, Z.; Wang, D.; Zhang, S.; Hu, L.; Jin, J., Interfacial Design of Mixed Matrix Membranes for Improved Gas Separation Performance. *Adv. Mater.* **2016,** *28* (17), 3399-3405.

(2) Shi, Y.; Liu, Y.; Wang, Z.; Lai, W.; Liao, Y.; Lu, K.; Niu, Z.; Jin, J., Dual-Wing Ligand Constructed Metal–Organic Framework Membranes with Finely Tuned Apertures for Natural Gas Separation. *Adv. Funct. Mater.* **2024,** *34* (46), 2404681.

(3) Peng, Y.; Wang, Y.; Li, W.; Jin, J., Bio-inspired vertically aligned polyaniline nanofiber layers enabling extremely high-efficiency solar membrane distillation for water purification. *J. Mater. Chem. A* **2021,** *9* (17), 10678-10684.

(4) Mateo, E.; Lahoz, R.; de la Fuente, G. F.; Paniagua, A.; Coronas, J.; Santamaría, J., Growth of Silicalite-1 by a Method Involving Separation of Reactants. *Chem. Mater.* **2007,** *19* (3), 594-599.

(5) Dubbeldam, D.; Calero, S.; Ellis, D. E.; Snurr, R. Q., RASPA: molecular simulation software for adsorption and diffusion in flexible nanoporous materials. *Mol. Simul.* **2016,** *42* (2), 81-101.

(6) Rappe, A. K.; Casewit, C. J.; Colwell, K. S.; Goddard, W. A., III; Skiff, W. M., UFF, a full periodic table force field for molecular mechanics and molecular dynamics simulations. *J. Am. Chem. Soc.* **1992,** *114* (25), 10024-10035.

(7) Mayo, S. L.; Olafson, B. D.; Goddard, W. A., DREIDING: a generic force field for molecular simulations. *J. Phys. Chem.* **1990,** *94* (26), 8897-8909.

(8) Plimpton, S., Fast Parallel Algorithms for Short-Range Molecular Dynamics. *J. Comput. Phys.* **1995,** *117* (1), 1-19.

(9) Stukowski, A., Visualization and analysis of atomistic simulation data with OVITO–the Open Visualization Tool. *Modell. Simul. Mater. Sci. Eng.* **2010,** *18* (1), 015012.

(10) Zhang, Y.; Suo, B.; Wang, Z.; Zhang, N.; Li, Z.; Lei, Y.; Zou, W.; Gao, J.; Peng, D.; Pu, Z.; Xiao, Y.; Sun, Q.; Wang, F.; Ma, Y.; Wang, X.; Guo, Y.; Liu, W., BDF: A relativistic electronic structure program package. *J. Chem. Phys.* **2020,** *152* (6), 064113.

(11) Zhang, J.; Lu, T., Efficient evaluation of electrostatic potential with computerized optimized code. *PCCP* **2021,** *23* (36), 20323-20328.

(12) Lu, T.; Chen, F., Multiwfn: A multifunctional wavefunction analyzer. *J. Comput. Chem.* **2012,** *33* (5), 580-592.

(13) Humphrey, W.; Dalke, A.; Schulten, K., VMD: Visual molecular dynamics. *J. Mol. Graphics* **1996,** *14* (1), 33-38.

(14) Zheng, P.; Xie, W.; Liu, H.; Han, T.; Wang, H.; Cui, S.; Peng, N.; Fan, Q.; Luo, S., Process Design and Techno-Economic Analysis of Membrane-Mediated Helium Recovery from Low-Grade Natural Gas. *Ind. Eng. Chem. Res.* **2023,** *62* (50), 21770-21776.

(15) Quader, M. A.; Rufford, T. E.; Smart, S., Modeling and cost analysis of helium recovery using combined-membrane process configurations. *Sep. Purif. Technol.* **2020,** *236*, 116269.
(16) Scholes, C. A.; Gosh, U. K.; Ho, M. T., The Economics of Helium Separation and Purification by Gas Separation Membranes. *Ind. Eng. Chem. Res.* **2017,** *56* (17), 5014-5020.
(17) Quader, M. A.; Rufford, T. E.; Smart, S., Integration of hybrid membrane-distillation processes to recover helium from pre-treated natural gas in liquefied natural gas plants. *Sep. Purif. Technol.* **2021,** *263*, 118355.
(18) Rufford, T. E.; Chan, K. I.; Huang, S. H.; May, E. F., A Review of Conventional and Emerging Process Technologies for the Recovery of Helium from Natural Gas. *Adsorpt. Sci. Technol.* **2014,** *32* (1), 49-72.
(19) Jin, D.; Xiao, G.; Lu, Z.; Weh, R.; McElroy, L.; Hu, G.; May, E. F., Industrial advances in helium recovery and purification technologies: a review. *Sep. Purif. Technol.* **2026,** *380*, 135136.
(20) Wang, J.; Ding, Y.; He, M.; Ding, X.; Liu, X.; Shi, W., Direct Preparation of Ultrathin Polymer Membranes on Porous Substrates for the Separation of Helium From Methane. *Small* **2025,** *21* (4), 2406440.
(21) Zheng, P.; Xie, W.; Cai, Z.; Jiao, Y.; Sun, Y.; Han, T.; Ma, X.; Li, N.; Luo, S., Ionization of Tröger's base polymer of intrinsic microporosity for high-performance membrane-mediated helium recovery. *J. Membr. Sci.* **2023,** *672*, 121425.
(22) Xie, W.; Jiao, Y.; Cai, Z.; Liu, H.; Gong, L.; Lai, W.; Shan, L.; Luo, S., Highly Selective Benzimidazole-Based Polyimide/Ionic Polyimide Membranes for Pure- and Mixed-Gas CO2/CH4 Separation. *Sep. Purif. Technol.* **2022,** *282*, 120091.
(23) Ma, X.; Li, K.; Zhu, Z.; Dong, H.; Lv, J.; Wang, Y.; Pinnau, I.; Li, J.; Chen, B.; Han, Y., High-performance polymer molecular sieve membranes prepared by direct fluorination for efficient helium enrichment. *J. Mater. Chem. A* **2021,** *9* (34), 18313-18322.
(24) Wang, L.; Li, Y.; Zhang, P.; Chen, X.; Nian, P.; Wei, Y.; Lu, H.; Gu, X.; Wang, X., Thermally rearranged poly(benzoxazole-co-imide) composite membranes on α-Al2O3 support for helium extraction from natural gas. *J. Membr. Sci.* **2022,** *657*, 120614.
(25) Yavari, M.; Fang, M.; Nguyen, H.; Merkel, T. C.; Lin, H.; Okamoto, Y., Dioxolane-Based Perfluoropolymers with Superior Membrane Gas Separation Properties. *Macromolecules* **2018,** *51* (7), 2489-2497.
(26) Fang, M.; He, Z.; Merkel, T. C.; Okamoto, Y., High-performance perfluorodioxolane copolymer membranes for gas separation with tailored selectivity enhancement. *J. Mater. Chem. A* **2018,** *6* (2), 652-658.
(27) Wang, X.; Shan, M.; Liu, X.; Wang, M.; Doherty, C. M.; Osadchii, D.; Kapteijn, F., High-Performance Polybenzimidazole Membranes for Helium Extraction from Natural Gas. *ACS Appl. Mater. Interfaces* **2019,** *11* (22), 20098-20103.
(28) Li, Y.; Wang, L.; Xie, J.; Dai, Y.; Gu, X.; Wang, X., Asymmetric copolyimide membranes fabricated by nonsolvent-induced phase separation for He/CH4 and He/N2 separation. *Front. Chem. Sci. Eng.* **2024,** *18* (4), 44.
(29) Seong, J. G.; Lee, W. H.; Lee, J.; Lee, S. Y.; Do, Y. S.; Bae, J. Y.; Moon, S. J.; Park,

C. H.; Jo, H. J.; Kim, J. S.; Lee, K.-R.; Hung, W.-S.; Lai, J.-Y.; Ren, Y.; Roos, C. J.; Lively, R. P.; Lee, Y. M., Microporous polymers with cascaded cavities for controlled transport of small gas molecules. *Sci. Adv.* **2021,** *7* (40), eabi9062.
(30) Peng, N.; Chung, T.-S.; Chng, M. L.; Aw, W., Evolution of ultra-thin dense-selective layer from single-layer to dual-layer hollow fibers using novel Extem® polyetherimide for gas separation. *J. Membr. Sci.* **2010,** *360* (1), 48-57.
(31) Gantzel, P. K.; Merten, U., Gas Separations with High-Flux Cellulose Acetate Membranes. *Ind. Eng. Chem. Process Des. Dev.* **1970,** *9* (2), 331-332.
(32) Choi, S.-H.; Sultan, M. M. B.; Alsuwailem, A. A.; Zuabi, S. M., Preparation and characterization of multilayer thin-film composite hollow fiber membranes for helium extraction from its mixtures. *Sep. Purif. Technol.* **2019,** *222*, 152-161.
(33) Kosuri, M. R.; Koros, W. J., Defect-free asymmetric hollow fiber membranes from Torlon®, a polyamide–imide polymer, for high-pressure CO2 separations. *J. Membr. Sci.* **2008,** *320* (1), 65-72.
(34) Favvas, E. P.; Heliopoulos, N. S.; Papageorgiou, S. K.; Mitropoulos, A. C.; Kapantaidakis, G. C.; Kanellopoulos, N. K., Helium and hydrogen selective carbon hollow fiber membranes: The effect of pyrolysis isothermal time. *Sep. Purif. Technol.* **2015,** *142*, 176-181.
(35) Chatzidaki, E. K.; Favvas, E. P.; Papageorgiou, S. K.; Kanellopoulos, N. K.; Theophilou, N. V., New polyimide–polyaniline hollow fibers: Synthesis, characterization and behavior in gas separation. *Eur. Polym. J.* **2007,** *43* (12), 5010-5016.
(36) Lively, R. P.; Dose, M. E.; Xu, L.; Vaughn, J. T.; Johnson, J. R.; Thompson, J. A.; Zhang, K.; Lydon, M. E.; Lee, J.-S.; Liu, L.; Hu, Z.; Karvan, O.; Realff, M. J.; Koros, W. J., A high-flux polyimide hollow fiber membrane to minimize footprint and energy penalty for CO2 recovery from flue gas. *J. Membr. Sci.* **2012,** *423-424*, 302-313.
(37) Li, Z.; Han, T.; Lai, W.; Ma, J.; Zhang, Y.; Wu, Q.; Wang, C.; Liao, C.; Luo, S., Enhanced plasticization resistance of hollow fiber membranes for helium recovery from natural gas based on a novel thermally crosslinkable polyimide. *J. Membr. Sci.* **2023,** *688*, 122126.
(38) Li, Z.; Lai, W.; Sun, Y.; Han, T.; Liu, X.; Liao, C.; Luo, S., Enhanced plasticization resistance of hollow fiber membranes via metal ion coordination for advanced helium recovery. *J. Membr. Sci.* **2025,** *715*, 123480.
(39) Li, Z.; Liu, X.; Sun, Y.; Gong, L.; Liao, C.; Luo, S., Pressure-resistant polyimide hollow fiber membranes for high-performance helium recovery from natural gas. *Polymer* **2025,** *323*, 128164.
(40) Wu, Q.; Liu, L.; Jiao, Y.; Li, Z.; Bai, J.; Ma, X.; Luo, S.; Zhang, S., Precise Helium Sieving from Hydrogen Using Fluorine-Decorated Carbon Hollow Fiber Membranes. *Angew. Chem. Int. Ed.* **2024,** *63* (33), e202400688.
(41) Wang, C.; Li, Z.; Bai, J.; Liu, H.; Wang, X.; Luo, S., Dual thermally crosslinked hollow fiber membranes for plasticization-resistant helium recovery from natural gas. *Sep. Purif. Technol.* **2025,** *354*, 129019.
(42) Wang, C.; Chen, X.; Liu, X.; Li, Z.; Liu, R.; Luo, S.; Zhang, S., Plasma-Engineered Sub-10 nm Surface Fluorination Enables Ultraselective Hollow Fiber Membranes.

Angew. Chem. Int. Ed. **2025,** *64* (37), e202512119.
(43) Kumakiri, I.; Tamura, K.; Sasaki, Y.; Tanaka, K.; Kita, H., Influence of Iron Additive on the Hydrogen Separation Properties of Carbon Molecular Sieve Membranes. *Ind. Eng. Chem. Res.* **2018,** *57* (15), 5370-5377.
(44) Ogieglo, W.; Puspasari, T.; Ma, X.; Pinnau, I., Sub-100 nm carbon molecular sieve membranes from a polymer of intrinsic microporosity precursor: Physical aging and near-equilibrium gas separation properties. *J. Membr. Sci.* **2020,** *597*, 117752.
(45) Centeno, T. A.; Fuertes, A. B., Carbon molecular sieve gas separation membranes based on poly(vinylidene chloride-co-vinyl chloride). *Carbon* **2000,** *38* (7), 1067-1073.
(46) Lee, H.-J.; Suda, H.; Haraya, K.; Moon, S.-H., Gas permeation properties of carbon molecular sieving membranes derived from the polymer blend of polyphenylene oxide (PPO)/polyvinylpyrrolidone (PVP). *J. Membr. Sci.* **2007,** *296* (1), 139-146.
(47) Ogieglo, W.; Puspasari, T.; Hota, M. K.; Wehbe, N.; Alshareef, H. N.; Pinnau, I., Nanohybrid thin-film composite carbon molecular sieve membranes. *Mater. Today Nano* **2020,** *9*, 100065.
(48) Khan, S.; Wang, K.; Feng, X.; Elkamel, A., Carbon molecular sieve membranes for natural gas purification: Role of surface flow. *Can. J. Chem. Eng.* **2020,** *98* (3), 775-784.
(49) Feng, C.; Guo, H.; Deng, M.; Wei, J.; Ma, Y.; Qin, Z.; Ma, X.; Liu, J.; Deng, L.; Jiang, W.; Yang, L.; Yao, L.; Dai, Z., Thin-Film-Composite Carbon Molecular Sieve Membranes for Efficient Helium and Hydrogen Separation. *Ind. Eng. Chem. Res.* **2024,** *63* (1), 594-606.
(50) Zhang, C.; Zhao, G.; Li, Z.; Lei, L.; Xu, Z., Hierarchical porous carbon membranes for lean-helium extraction from high-pressure natural gas. *AlChE J.* **2025,** *71* (6), e18790.
(51) Liu, L.; Li, Q.; Sun, L.; Riaz, A.; Li, J.; Wang, Y.; Pinnau, I.; Ma, X., Ultrahigh He enrichment property of carbon molecular sieve membranes by direct fluorination. *J. Membr. Sci.* **2025,** *717*, 123647.
(52) Denning, S.; Lucero, J.; Koh, C. A.; Carreon, M. A., Chabazite Zeolite SAPO-34 Membranes for He/CH_4 Separation. *ACS Mater. Lett.* **2019,** *1* (6), 655-659.
(53) Funke, H. H.; Chen, M. Z.; Prakash, A. N.; Falconer, J. L.; Noble, R. D., Separating molecules by size in SAPO-34 membranes. *J. Membr. Sci.* **2014,** *456*, 185-191.
(54) Poshusta, J. C.; Tuan, V. A.; Pape, E. A.; Noble, R. D.; Falconer, J. L., Separation of light gas mixtures using SAPO-34 membranes. *AlChE J.* **2000,** *46* (4), 779-789.
(55) Karakiliç, P.; Wang, X.; Kapteijn, F.; Nijmeijer, A.; Winnubst, L., Defect-free high-silica CHA zeolite membranes with high selectivity for light gas separation. *J. Membr. Sci.* **2019,** *586*, 34-43.
(56) Deng, Y.; Qiu, F.; Wu, J.; Li, J.; Li, Z.; Huang, Q.; Zhu, G.; Zou, X., Flexible ZSM-5 Zeolite Membrane for High-Performance Helium Separation. *Angew. Chem. Int. Ed.* **2025,** *64* (6), e202421285.
(57) Gong, C.; Peng, X.; Zhu, M.; Zhou, T.; You, L.; Ren, S.; Wang, X.; Gu, X., Synthesis and performance of STT zeolite membranes for He/N2 and He/CH4 separation. *Sep. Purif. Technol.* **2022,** *301*, 121927.
(58) Li, Y.; Zhou, T.; Pu, L.; Dai, Y.; Yang, M.; Zhu, M.; Wang, X.; Gu, X., Preparation

of STT zeolite membranes with outstanding helium extraction performance by gradient induction method. *J. Membr. Sci.* **2025,** *717*, 123625.
(59) Liu, Y.; Qiang, W.; Lu, J.; Liu, Y., Fabrication of highly a-oriented ultrathin FER zeolite membrane from nanosheets towards small-sized gas separation. *J. Membr. Sci.* **2025,** *734*, 124437.
(60) Hosseini, S. S.; Li, Y.; Chung, T.-S.; Liu, Y., Enhanced gas separation performance of nanocomposite membranes using MgO nanoparticles. *J. Membr. Sci.* **2007,** *302* (1), 207-217.
(61) Molavi, H.; Shojaei, A.; Mousavi, S. A., Improving mixed-matrix membrane performance via PMMA grafting from functionalized NH2–UiO-66. *J. Mater. Chem. A* **2018,** *6* (6), 2775-2791.
(62) Kim, S.; Jinschek, J. R.; Chen, H.; Sholl, D. S.; Marand, E., Scalable Fabrication of Carbon Nanotube/Polymer Nanocomposite Membranes for High Flux Gas Transport. *Nano Lett.* **2007,** *7* (9), 2806-2811.
(63) Zhang, Z.; Yang, Z.; Li, S.; Qian, L.; Chen, X.; Chen, G.; Liu, G.; Jin, W., Hot solution strategy to prepare Zr-MOF/polyimide mixed matrix membranes for high-performance helium separation. *J. Membr. Sci.* **2025,** *729*, 124137.
(64) Komal, A.; Calderón-Rodríguez, L.; Smirnova, O.; Grossmann, E.; Varghese, A. B.; Garcia Alvarez, K. M.; Schneemann, A.; Hoyer, T.; Wyrwa, R.; Schacher, F. H.; Knebel, A., Ideal Molecular Sieving with a Dense MOF for Helium Upgrading with Highly Diffusion Selective Mixed Matrix Membranes. *Adv. Funct. Mater.* **2025,** *n/a* (n/a), 2423999.
(65) Han, J.; Wu, H.; Fan, H.; Ding, L.; Hai, G.; Caro, J.; Wang, H., Tuning the Phase Composition of Metal–Organic Framework Membranes for Helium Separation through Incorporation of Fullerenes. *J. Am. Chem. Soc.* **2023,** *145* (27), 14793-14801.
(66) Cao, F.; Zhang, C.; Xiao, Y.; Huang, H.; Zhang, W.; Liu, D.; Zhong, C.; Yang, Q.; Yang, Z.; Lu, X., Helium Recovery by a Cu-BTC Metal–Organic-Framework Membrane. *Ind. Eng. Chem. Res.* **2012,** *51* (34), 11274-11278.
(67) Yoo, Y.; Varela-Guerrero, V.; Jeong, H.-K., Isoreticular Metal−Organic Frameworks and Their Membranes with Enhanced Crack Resistance and Moisture Stability by Surfactant-Assisted Drying. *Langmuir* **2011,** *27* (6), 2652-2657.
(68) Takamizawa, S.; Takasaki, Y.; Miyake, R., Single-Crystal Membrane for Anisotropic and Efficient Gas Permeation. *J. Am. Chem. Soc.* **2010,** *132* (9), 2862-2863.
(69) Liu, D.; Ma, X.; Xi, H.; Lin, Y. S., Gas transport properties and propylene/propane separation characteristics of ZIF-8 membranes. *J. Membr. Sci.* **2014,** *451*, 85-93.
(70) Zhao, Z.; Ding, L.; Hinterding, R.; Mundstock, A.; Belke, C.; Haug, R. J.; Wang, H.; Feldhoff, A., MXene assisted preparation of well-intergrown ZIF-67 membrane for helium separation. *J. Membr. Sci.* **2022,** *652*, 120432.
(71) Liu, Z.; Liu, Y.; Wang, H.; Qu, Z.; Hou, J.; Meng, H.; Fan, H., Orifice-Modified MOF-on-MOF Bilayer Membrane for Helium Separation. *Ind. Eng. Chem. Res.* **2025,** *64* (11), 6106-6113.
(72) Zhao, Z.; Ding, L.; Mundstock, A.; Stölting, O.; Polarz, S.; Wang, H.; Feldhoff, A., Preparation of ZIF-62 polycrystalline and glass membranes for helium separation. *J. Membr. Sci.* **2024,** *700*, 122677.

(73) Bai, J.; Xiao, L.; Zhang, X.; Liu, H.; Wang, C.; Gong, L.; Luo, S.; Zhu, Y.; Shan, L.; Fan, H.; Zhang, S., Metallocene-anchor inducing oriented MOF membrane for helium separation. *Nat. Commun.* **2025,** *16* (1), 9451.
